# Highly Selective Synthesis of Seven-Membered Azaspiro Compounds by a Rh(I)-Catalyzed Cycloisomerization/Diels–Alder Cascade of 1,5-Bisallenes

**DOI:** 10.1021/acs.joc.2c00065

**Published:** 2022-03-24

**Authors:** Jordi Vila, Miquel Solà, Anna Pla-Quintana, Anna Roglans

**Affiliations:** Institut de Química Computacional i Catàlisi (IQCC) and Departament de Química, Facultat de Ciències, Universitat de Girona (UdG), C/ Maria Aurèlia Capmany, 69, Girona 17003, Catalonia, Spain

## Abstract

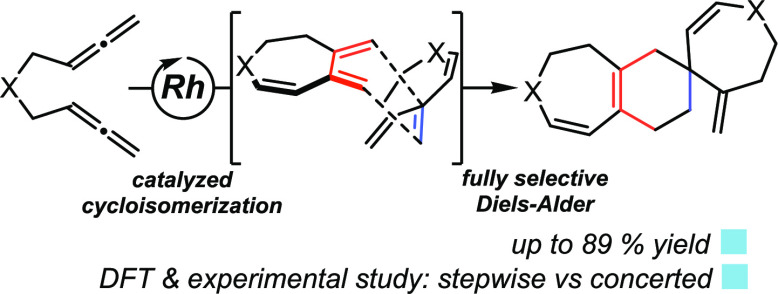

The synthesis of spiro compounds featuring seven- and six-membered rings in the spirobicyclic motif is successfully achieved through a cascade process encompassing a rhodium(I)-catalyzed cycloisomerization followed by a highly selective Diels–Alder homodimerization. The scope of the reaction is analyzed based on a series of synthetic substrates, and control experiments and DFT calculations led us to justify the exquisite degree of selectivity observed.

## Introduction

The synthesis of spiro compounds, structures containing two rings connected through a single carbon atom (referred to as spiro atoms), has received significant attention. This can be explained by their broad spectrum of biological and pharmaceutical activity^1,2^ as well as their applications in organic optoelectronics^3^ and catalysis.^4^ The presence of a spirobicyclic scaffold in a molecule imposes an inherent rigidity that greatly influences its chemical and physical properties. In drug discovery, for example, the rigidity of the spirobicyclic motif has been used to design the 3D orientation of the pharmacophore more efficiently. This maximizes intermolecular interactions and thus improves the recognition process.^5^ However, the presence in a molecule of a quaternary spiro atom, which can present central or axial chirality, poses major challenges for its synthesis.^6^

**Figure 1:**
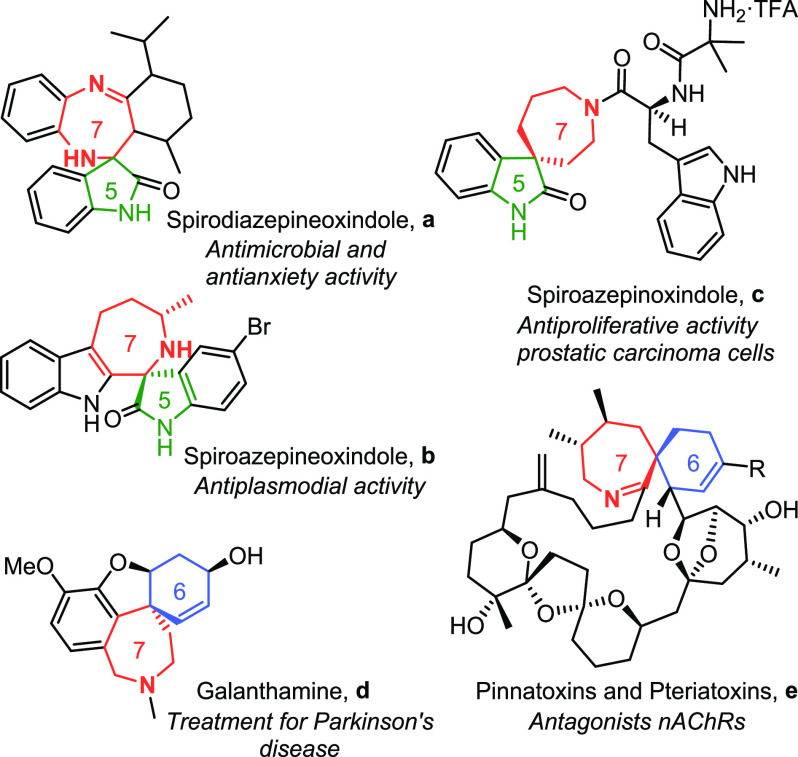
Bioactive molecules that contain seven-membered azaspiro scaffolds.

Plethoras of natural products contain spirobicyclic moieties, but spiro compounds containing a seven-membered ring in the spirobicyclic motif are not particularly common. However, those that contain a nitrogen atom in the seven-membered ring present very interesting pharmaceutical properties (Figure 1). Regarding derivatives of the spirooxindole family,^6c^ spirodiazepineoxindole **a** has been evaluated for its antimicrobial and antianxiety activity, providing notable results,^7^ and spiroazepineindole **b** shows antiplasmodial activity toward *Plasmodium falciparum*, the most relevant malaria parasite.^8^ A peptidomimetic containing the spiroazepinoxindole moiety (**c**, Figure 1) has also shown antiproliferative activity on human prostatic carcinoma cell lines.^9^ On the other hand, if we focus on spirobicyclic motifs containing six and seven cyclic rings, then galanthamine (**d**) is a natural product that is used to slow down the process of neurological degeneration in Alzheimer’s disease.^10^ Furthermore, spirolide derivatives^11^ such as pinnatoxins A–D and pteriatoxins A–C (**e**), isolated from *Pinna muricata*(12) and *Pteria penguin*(13) shellfish, respectively, are potent marine toxins containing a spirocyclic seven-membered imine that is key for its toxic activity.^14,15^

**Scheme 1:**
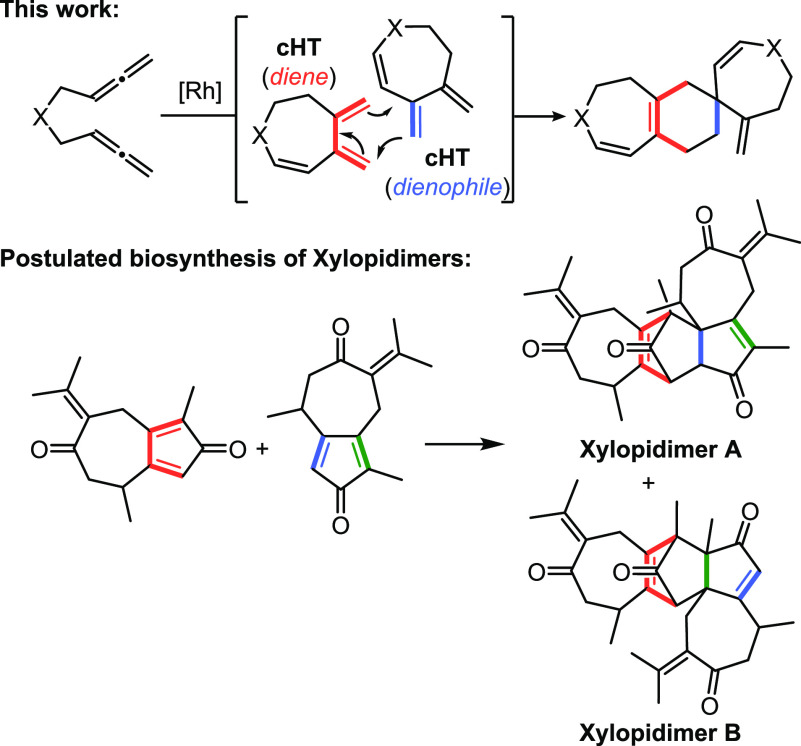
Synthesis of Spiro Derivatives through Diels–Alder Reactions

A straightforward strategy to construct a spirocyclic moiety containing a six-membered ring is based on the Diels–Alder cycloaddition between an exocyclic dienophile and a diene. In an ongoing project of our research group aimed at developing rhodium-catalyzed cyclization reactions involving allenes,^16^ we recently described^17^ a rhodium-catalyzed cycloisomerization/Diels–Alder cascade reaction of bisallenes and alkenes that afforded dihydroazepine- and dihydrooxepine-fused ring systems in good yields. In a control experiment excluding the alkene (that was designed to obtain mechanistic information), we observed that cycloheptatriene (**cHT**), obtained as a non-isolable intermediate in the reaction, dimerized through a Diels–Alder reaction to afford spirocyclic derivatives in a highly selective manner (Scheme 1). This dimerization is remarkably analogous to the postulated biosynthesis of xylopidimers A and B through [4 + 2] Diels–Alder cycloaddition of two guaiane moieties (Scheme 1), from which different orientations explain the formation of the various regioisomers that are isolated.^18^ Due to the interest both in the process and the properties of the products obtained, here we report the preparation of seven-membered azaspiro compounds and a full analysis of the reasons behind the exquisite degree of selectivity in their formation.

## Results and Discussion

**Figure 2:**
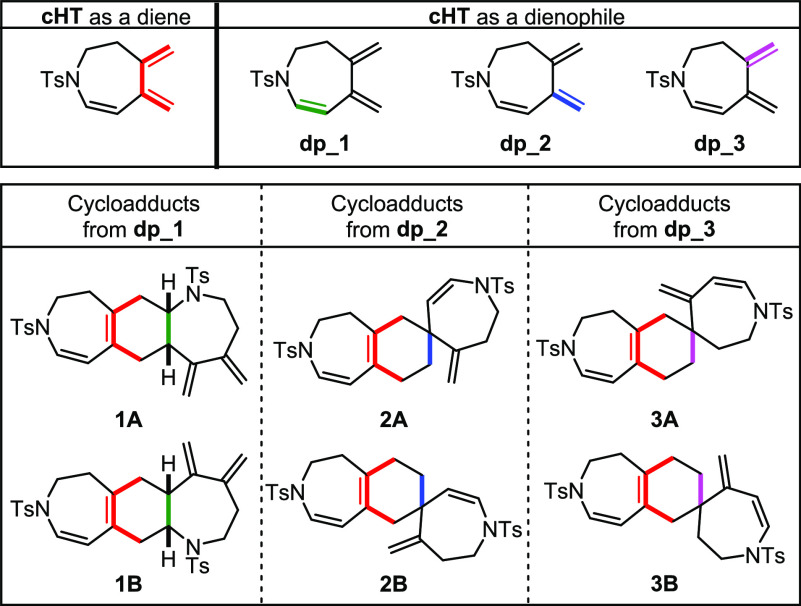
Possible cycloadducts that can be formed in the homo-Diels–Alder of cycloheptatriene **cHT**.

We started our study with *N*-tosyl-tethered bisallene **1a** (Table 1). Based on our earlier study,^17^ we carried out the dimerization of **1a** using the previously optimized reaction conditions (entry 1, Table 1). Employing 10 mol % of cationic rhodium complex [Rh(cod)_2_]BF_4_ with DTBM-Segphos in THF/CH_2_Cl_2_ (4:1), two different compounds were obtained: compound **2a** (47% yield) resulting from a dimerization reaction of intermediate **cHT**, isolated as a single regioisomer, and **3a** (14% yield) derived from a [2 + 2] cyclization of the two internal double bonds of the two allene moieties of **1a**.^19^ Since the *in situ*-generated cycloheptatriene **cHT** has three potential double bonds to be involved in the Diels–Alder reaction as dienophiles, we first proceeded to fully characterize the compound obtained. Figure 2 shows all the possible cycloadducts that can be formed in the homo-Diels–Alder cycloaddition of **cHT** ordered according to the double bond that acts as a dienophile (**dp**).

**Table 1:**
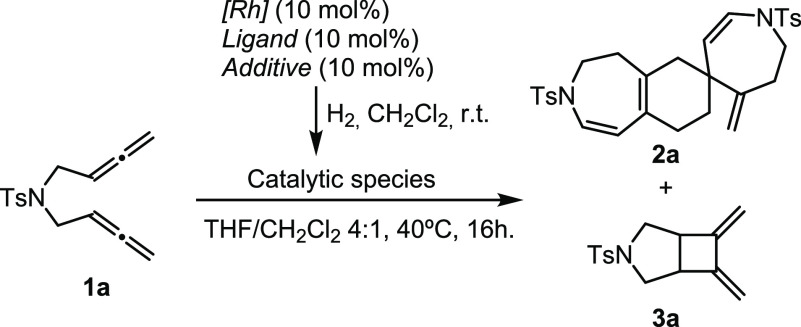
Optimization of the Rhodium(I)-Catalyzed Dimerization of Bisallene **1a**

entry	[Rh]	ligand^a^	[**1a**] mM	additive	yield (%) **2a**/**3a**
1	[Rh(cod)_2_]BF_4_	L1	9		47/14
2	[Rh(cod)_2_]BF_4_	L2	9		6/0
3	[Rh(cod)_2_]BF_4_	L3	9		10/0
4	[Rh(cod)_2_]BF_4_	L4	9		7/0
5	[Rh(cod)_2_]BF_4_	L1	4.5		44/17
6	[Rh(cod)_2_]BF_4_	L1	36		53/14
7	[Rh(cod)Cl]_2_	L1	36	AgSbF_6_	32/6
8	[Rh(cod)Cl]_2_	L1	36	NaBArF	46/6
9^b^	[Rh(cod)Cl]_2_	L1	36	NaBArF	89/11

a L1 = (*R*)-DTBM-Segphos/L2 = BINAP/L3 = Tol-BINAP/L4 = BIPHEP.

b The solvent mixture THF/DCM was non-anhydrous and degassed.

**Figure 3:**
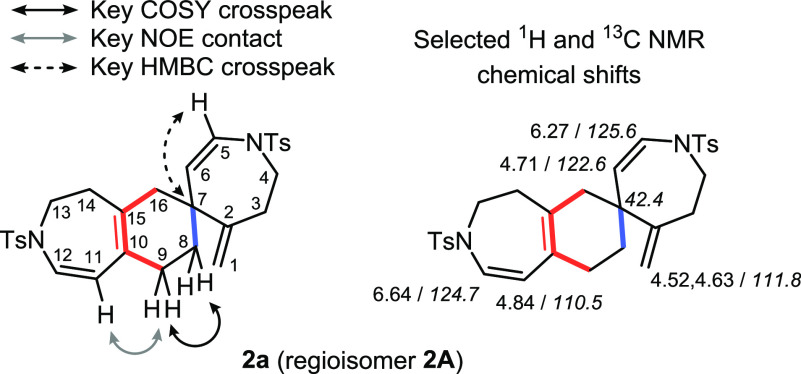
COSY and HMBC crosspeaks and NOE contacts observed in **2a** confirming the formation of regioisomer **2A** and selected ^1^H and ^13^C (in italics) NMR shifts of **2a**.

The molecular formula of dimeric compound **2a** was determined by HRMS, showing a peak at *m*/*z* = 573.1836 corresponding to the [M + Na]^+^ adduct. 1D and 2D NMR spectroscopic experiments clearly showed the formation of only one cycloadduct, and its complete analysis was carried out to ascertain the structure of the product formed. The ^1^H NMR chemical shift of the six olefinic protons and their multiplicity were first analyzed. Two pairs of doublets, at δ = 4.71 and 6.27 ppm and δ = 4.84 and 6.64 ppm, are characteristic of a structure that has two cis endocyclic double bonds. In addition, only two singlets at δ = 4.52 and 4.63 ppm are observed, corresponding to the two geminal protons of a single exocyclic double bond. This allowed us to discard cycloadducts **1A** and **1B**. In order to distinguish between cycloadducts formed by the reaction of **dp_2** and **dp_3**, 2D NMR experiments were conducted. The HMBC spectrum displays a three-bond correlation between the spiro carbon atom and the proton closest to the nitrogen of one of the cis endocyclic olefins (C7-H5, in Figure 3). This is, in fact, a clear demonstration that it is the exocyclic double bond conjugated to the endocyclic double bond that is involved in the Diels–Alder reaction (**dp_2**), and thus cycloadducts **3A** and **3B** can be discarded. Finally, the analysis of the HMBC, COSY, and NOESY experiments allowed us to distinguish between regioisomers **2A** and **2B** (Figure 3). NOE contacts between H9 (H8 and H9 identified as the two contiguous methylenic groups by COSY) and H11 protons led us to assign **2A** as the single cycloadduct that formed. The HMBC crosspeak between H11 and C9 also supports the formation of the cycloadduct **2A**. Selected signals in the ^1^H and ^13^C NMR spectra of **2a** are shown in Figure 3.

Since a highly chemo- and regioselective cascade process was found, we searched for the best reaction conditions to allow the efficient dimerization of bisallene **1a** while avoiding the formation of byproduct **3a** (Table 1).

First, the effect of the ligand was examined. BINAP, Tol-BINAP, and BIPHEP bisphosphines were tested, but spiro derivative **2a** was obtained in low yields (entries 2–4, Table 1). DPEphos, xantphos, and Xphos did not promote the reaction. Therefore, the bulky phosphine, DTBM-Segphos, was established as the ligand of choice. The reaction was then tested at both a lower and higher concentration of **1a** (entries 5 and 6, Table 1), and the yield rose up to 53% when the concentration was increased to 36 mM. The effect of an additive using the dimeric rhodium complex [Rh(cod)Cl]_2_ was next examined (entries 7 and 8, Table 1). The mixture of [Rh(cod)Cl]_2_/NaBArF maintained the yield of **2a** at 46% but decreased the yield of byproduct **3a** (6%) considerably. We then evaluated the effect of water on the reaction mixture. Using non-anhydrous solvents taken directly from the bottles but degassed, an 89% yield of **2a** was obtained (entry 9, Table 1), resulting in our set of optimized conditions. Since a chiral ligand was used, we checked the optical purity of **2a**; however, no enantioinduction was observed. This result points to a mechanism involving a rhodium-catalyzed cycloisomerization coupled to a Diels–Alder cycloaddition in which the chiral rhodium complex does not participate (*vide infra*), in line with our previous study.^17^

**Figure 4:**
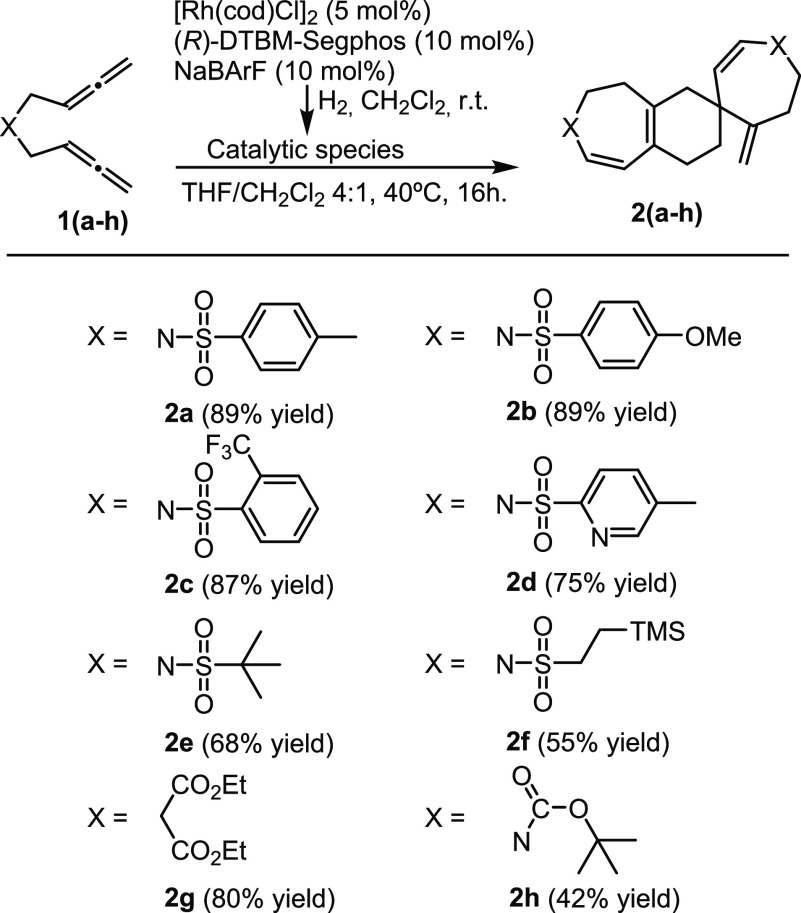
Scope of the cascade process. Byproducts **3a**–**3f** (5–13% yields) and **3g** (20% yield) were observed in the ^1^H NMR of the reaction crude.

The scope of the reaction was then evaluated (Figure 4). The nature of the substituents at the phenyl ring of the sulfonamide tether in bisallene **1a** was explored. The reaction proceeded efficiently with both electron-donating (**2b**) and electron-withdrawing groups (**2c**), and the substitution at the ortho position of the phenyl ring (**2c**) did not hamper the reaction. A bisallene bearing the 5-methyl-2-pyridinesulfonyl group provided **2d** in 75% yield, indicating that the presence of a potentially coordinating nitrogen atom did not poison the catalyst. Sulfonamide tethers with aliphatic substitution (*tert*-butyl and trimethylsilylethyl) were also efficient, delivering spiro derivatives **2e** and **2f** in 68 and 55% yields, respectively. Tethers other than sulfonamides were also tested. Carbon-tethered bisallene **1g** and *N*-Boc bisallene **1h** also participate in the cascade process, affording **2g** and **2h** with 80 and 42% yields, respectively.

In addition, 1 mmol-scale reactions using NTs-tethered bisallene **1a** and *N*-Boc-tethered bisallene **1h** were performed, affording 84 and 42% yields of **2a** and **2h**, respectively. In all the reactions, byproducts **3** were formed in low yields. Unfortunately, when we attempted heterodimerization reactions, a complex mixture was observed by NMR.

**Figure 5:**
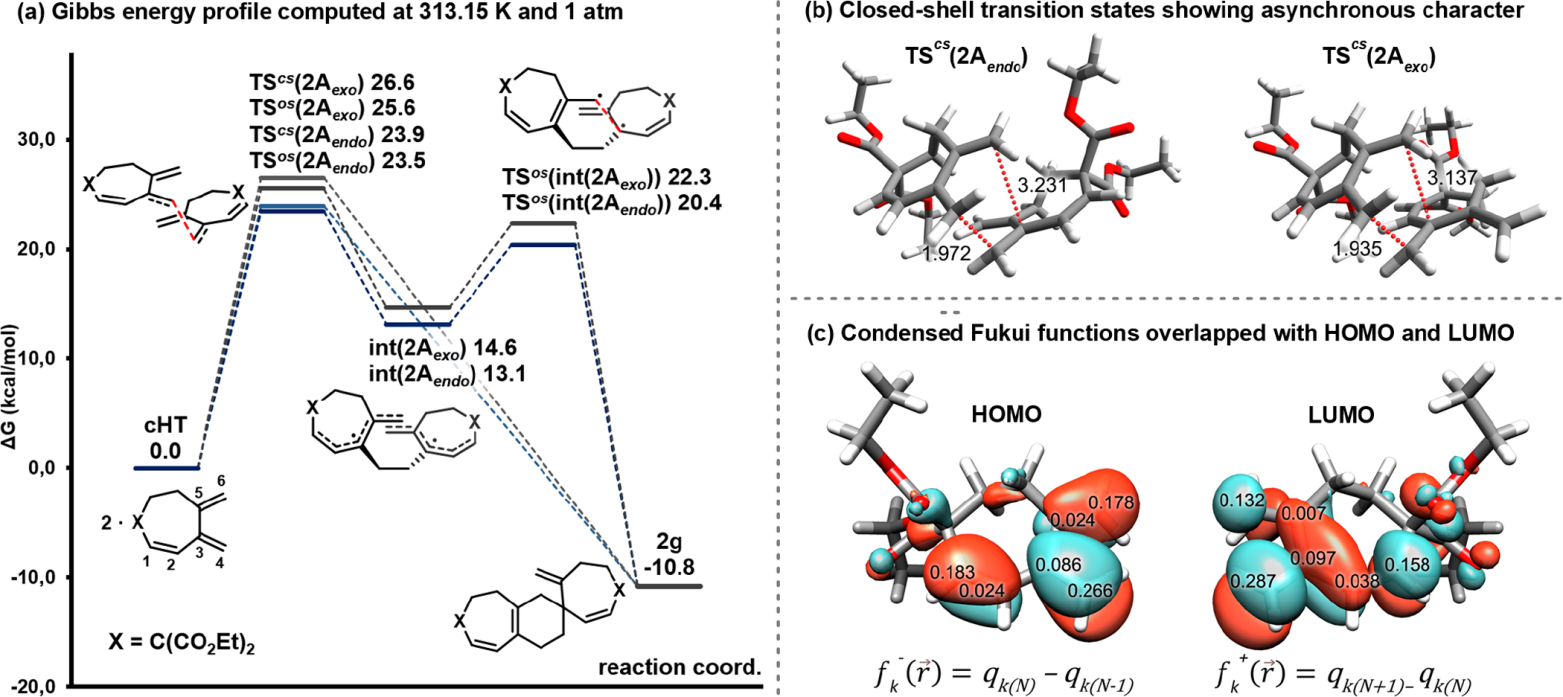
(a) Gibbs energy profile in kcal·mol^–1^ for the transformation of **cHT** (from **1g**) into **2g**. (b) Molecular structures of **TS^*cs*^(2A_*endo*_)** and **TS^*cs*^(2A_*exo*_)**. (c) Values of the condensed Fukui functions *f^–^* and *f^+^* (units are electrons) on the HOMO and LUMO orbitals.

To gain further understanding on the chemo- and regioselectivity on the formation of cycloadducts **2**, we completed our study by performing DFT calculations of the DA reaction of the **cHT**-**1g** (for the formation of cycloheptatriene **cHT**, see ref (17)). The Gibbs energy profile computed at 313.15 K and 1 atm with the (U)B3LYP-D3/cc-pVTZ/SMD(76% THF and 24% CH_2_Cl_2_)^20^//(U)B3LYP-D3/cc-pVDZ method is depicted in Figure 5; the Gibbs energy barriers and the molecular structures of all TSs can be found in Figures S7 and S8, respectively, and the closed-shell and open-shell singlet/triplet energies are in Table S1.

We first evaluated the formation of all the Diels–Alder cycloadducts shown in Figure 2, considering both the endo and exo approximations for each of them (see Figure S7 in the SI) using closed-shell (*cs*) calculations. We found that the lowest energy transition states are those that lead to the formation of our experimentally observed product, **TS^*cs*^(2A_*endo*_)** and **TS^*cs*^(2A_*exo*_)**, having barriers of 23.9 and 26.6 kcal·mol^–1^, respectively (see Figure S7 for detailed Gibbs energy barriers of all reaction paths). The geometries of these transition states present a high asynchronous character as shown in Figure 5b, suggesting the possibility of a two-step process. However, these transition states lead to product **2g** releasing 10.8 kcal·mol^–1^, and an intermediate could not be localized with the closed-shell calculations. In contrast to this, when performing open-shell (*os*) calculations, the reaction was found to occur in a two-step manner through a biradical intermediate. The transition states for the **2A_*endo*_** and **2A_*exo*_** approximations are slightly lower in energy than their closed-shell counterparts, with **TS^*os*^(2A_*endo*_)** (⟨*S*⟩^2^ = 0.05) being the lowest one by 0.4 kcal·mol^–1^ and preferred over **TS^*os*^(2A_*exo*_)** (⟨*S*⟩^2^ = 0.19) by 2.1 kcal·mol^–1^. This step generates **int(2A_*endo*_)** in an endergonic process by 13.1 kcal·mol^–1^. High delocalization of the unpaired electrons α and β of **int(2A_*endo/exo*_)** over the conjugated system has been observed for these intermediates (Figure S9). From this point, the collapse of the biradical intermediate **int(2A_*endo*_)** leads to **2g** through **TS^*os*^(int(2A_*endo*_))**, surpassing a Gibbs energy barrier of 7.3 kcal·mol^–1^ and releasing 23.9 kcal·mol^–1^. Although the two-step biradical pathway through the endo approximation is the lowest in energy, the difference of 0.4 kcal·mol^–1^ as compared to the concerted asynchronous mechanism for the endo approach is not sufficient to be able to distinguish between the two pathways and they are probably competing to generate the final product **2g**. However, it should be noted that the biradical pathway clearly helps in rationalizing the regio- and chemoselectivity observed, which is clearly governed by the stability of the biradical intermediate and has its two radicals delocalized in a double allylic position.

The condensed Fukui functions^21^ calculated from the natural population analysis (NPA) charges (*f^–^*_4_ = 0.266; *f^+^*_4_ = 0.287, Figure 5c) support the formation of the first bond between the unsubstituted terminus of the doubly conjugated exocyclic double bond of the two **cHT** units (C4–C4, atom labels in Figure 5a), leading to the selective formation of cycloadduct **2g** (Figure 2). The same conclusion can be extracted by looking at the HOMO and LUMO orbitals, as the best molecular orbital overlap is the one that comes from the coupling of the same methylene positions.

**Scheme 2:**
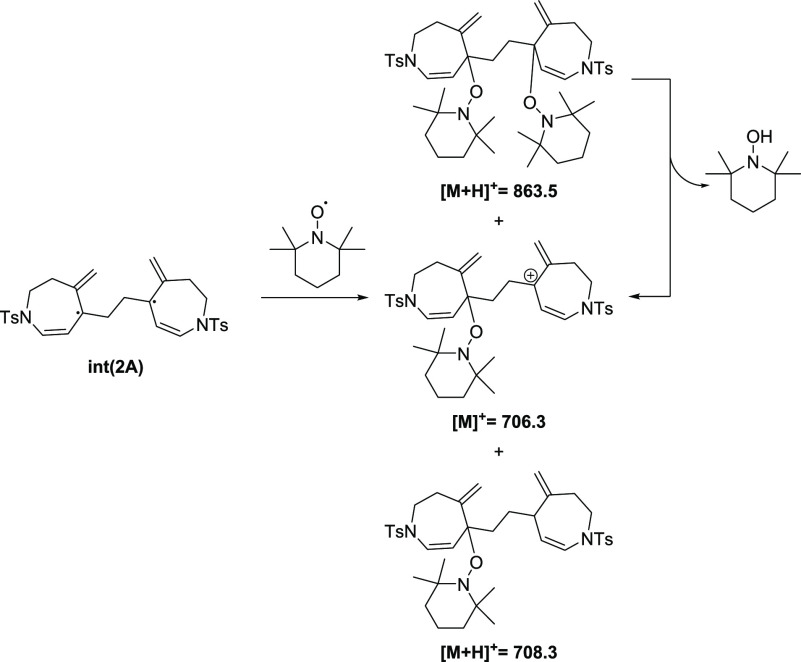
Species Detected by ESI(+)-MS when the Reaction Is Run in the Presence of TEMPO

To obtain experimental evidence of the proposed two-step biradical process, we performed the reaction in the presence of 1.5 equiv of TEMPO as a radical trapping agent.^22^ The reaction yield toward the formation of **2a** was drastically reduced from 89 to 44%, although the yield of **3a** was not significantly reduced. Furthermore, a fraction could be isolated from the reaction mixture that, when analyzed by ESI-MS, showed the incorporation into **int(2A)** of either two TEMPO moieties (giving rise to an adduct at *m*/*z* = 863.5 for [**int(2A)** + 2TEMPO + H]^+^ that fragmented to a species at *m*/*z* = 706.3 by the loss of neutral TEMPO-H as confirmed by MS/MS) or a hydrogen radical and a TEMPO (giving rise to an adduct at *m*/*z* = 708.3 for [**int(2A)** + TEMPO+H· + H]^+^) (Scheme 2). This experiment gives experimental evidence of the intermediacy of biradical intermediates in the reaction under study.

## Conclusions

In summary, a method that enables rapid access to seven- and six-membered spirocyclic compounds in a complete chemo- and regioselective manner from 1,5-bisallenes has been developed. The whole process involves an initial rhodium-catalyzed cycloisomerization of 1,5-bisallenes leading to the non-isolable cycloheptatrienes followed by Diels–Alder homodimerization. A set of aromatic and aliphatic sulfonamide-tethered 1,5-bisallenes, as well as a carbon-tethered 1,5-bisallene, gave the final spirocyclic product with complete chemo- and regioselectivity. The DFT calculations have demonstrated the selectivity observed, arising from the highly favored homocoupling of the unsubstituted terminus of the doubly conjugated double bond. Additionally, although experimental evidence has been found for the formation of the proposed biradical intermediate, the computational study suggests that both mechanisms, the two-step biradical and the concerted asynchronous, compete to generate the spirocyclic products.

## Experimental Section

### General Information

Unless otherwise noted, materials were obtained from commercial suppliers and used without further purification. Bisallenes **1a** (X = NTs), **1b** (X = p-MeO-PhSO_2_N), **1c** (X = *o*-CF_3_-PhSO_2_N), **1d** (X = 5-Me-2-PySO_2_N), **1e** (X = ^*t*^Bu-SO_2_N), **1f** (X = TMS-CH_2_CH_2_-SO_2_N), **1g** (X = C(COOEt)_2_), and **1h** (X = *N*-Boc) were prepared from the corresponding bisalkynes using Crabbé homologation reaction. Experimental procedures and full characterization have been described previously by us.^16f^

CH_2_Cl_2_ and THF were dried under nitrogen by passing through solvent purification columns (MBraun, SPS-800). Reaction progress during the preparation of all compounds was monitored using thin-layer chromatography on Macherey-Nagel Xtra SIL G/UV254 silica gel plates. Solvents were removed under reduced pressure with a rotary evaporator. Reaction mixtures were chromatographed on silica gel using an automated purification instrument Interchim PuriFlash XS 520 Plus equipped with a quaternary gradient pump (up to 300 mL/min, 20 bar) and a UV–vis 200–800 nm diode array detector. All ^1^H and ^13^C NMR spectra were recorded on a Bruker ASCEND 400 spectrometer equipped with a 5 mm BBFO probe using CDCl_3_ as a deuterated solvent. Chemical shifts for ^1^H and ^13^C NMR are reported in ppm (δ) relative to residual solvent signals (7.26 ppm for 1H and 77.16 ppm for ^13^C). Coupling constants are given in hertz (Hz). ^1^H and ^13^C NMR signals were assigned based on 2D-NMR HSQC, HMBC, COSY, and NOESY experiments. Electrospray mass spectrometry analyses were recorded on an Esquire 6000 ion trap mass spectrometer (Bruker) equipped with an electrospray ion source. Electrospray ionization high-resolution mass spectrometry was performed using a Bruker microTOF-Q II instrument. Both mass instruments were operated in the positive ESI(+) ion mode. IR spectra were recorded on an Agilent Cary 630 FT-IR spectrometer equipped with an ATR sampling accessory. Melting points were measured in an SMP10 apparatus from Stuart without any correction.

### Computational Details

Geometries of all stationary points were optimized without symmetry constraint with the Gaussian 16 program^23^ using the DFT B3LYP hybrid exchange-correlation functional^24^ employing the all-electron cc-pVDZ basis set,^25^ and **TS^*os*^(2A**_exo_) and **TS^*os*^(2A**_endo_) were optimized again using UB3LYP/cc-pVDZ. The electronic energies were improved (singlet and triplet states) by performing single-point energy calculations with the cc-pVTZ basis set and the (U)B3LYP hybrid exchange-correlation functional including solvent effect corrections computed with the solvent model based on density (SMD) continuum solvation.^25^ To mimic the experimental solvent mixture with a molar fraction ratio of 76:24 of THF:CH_2_Cl_2_, the values of the solvent descriptors used in the SMD solvation model were redefined on the basis of a linear behavior with the molar fraction. Using the “Solvent = (Generic,Read)” options of the Gaussian16 SCRF keyword, the solvent mixture was defined employing the following solvent descriptors: dynamic dielectric constant = 1.410; static dielectric constant = 1.4085; Abraham’s hydrogen bond acidity = 0.02424; Abraham’s hydrogen bond basicity = 0.3758; surface tension = 39.3697; carbon aromaticity = 0; electronegativity halogenicity = 0.1617. The D3 Grimme energy corrections for dispersion^26^ with the original damping function were added in all (U)B3LYP/cc-pVDZ and (U)B3LYP/cc-pVTZ calculations. Analytical Hessians were computed to determine the nature of stationary points (one and zero imaginary frequencies for TSs and minima, respectively) and to calculate unscaled zero-point energies as well as thermal corrections and entropy effects using the standard statistical-mechanics relationships for an ideal gas.^27^ These two latter terms were computed at 313.15 K and 1 atm to provide the reported relative Gibbs energies. As a summary, the reported Gibbs energies contain electronic energies including solvent effects calculated at the (U)B3LYP-D3/cc-pVTZ//(U)B3LYP-D3/cc-pVDZ level together with gas-phase thermal and entropic contributions computed at 313.15 K and 1 atm with the (U)B3LYP-D3/cc-pVDZ method. All stationary points were unambiguously confirmed by IRC calculations. For the condensed Fukui functions, the natural charges were obtained by performing the natural population analysis of the neutral, cationic, and anionic **cHT** at the (U)B3LYP-D3/cc-pVTZ//(U)B3LYP-D3/cc-pVDZ theory level.

### General Procedure for the Synthesis of Spiro Derivatives **2**

In a 10 mL capped vial, a mixture of [Rh(cod)Cl]_2_ (2.2 mg, 0.004 mmol, 0.05 equiv), (*R*)-DTBM-Segphos (11.3 mg, 0.01 mmol, 0.10 equiv), and NaBArF (8.5 mg, 0.01 mmol, 0.10 equiv) was purged with nitrogen and dissolved in anhydrous CH_2_Cl_2_ (4 mL). Hydrogen gas was bubbled into the catalyst solution, and the mixture was stirred for 30 min. The mixture was then concentrated to dryness under a stream of hydrogen, dissolved again in degassed CH_2_Cl_2_ (0.5 mL), and transferred via a syringe into a solution of bisallene **1a**–**g** (0.09 mmol, 1 equiv) in degassed THF (2 mL) under an inert atmosphere at 40 °C (aluminum heating block). The resulting mixture was stirred for 16 h at 40 °C. The solvent was removed under reduced pressure, and the crude reaction mixture was purified by column chromatography on silica gel using mixtures of hexane/EtOAc as the eluent (90:10 to 60:40 v/v).

### Spiro Derivative **2a**

Compound **2a** was obtained from bisallene **1a** (25 mg, 0.09 mmol) following the general procedure. Purification by column chromatography (silica gel, 40–63 μm, hexanes/EtOAc: 90:10 to 60:40 v/v) provided **2a** (22.3 mg, 89% yield) as a colorless solid. MP (°C): 87–92 (dec); IR (ATR) ν (cm^–1^): 2921, 1336, 1156. ^1^H NMR (CDCl_3_, 400 MHz): δ 7.66 (m, 4H), 7.31 (d, 2H, ^3^*J_ortho_* = 8.2 Hz), 7.28 (d, 2H, ^3^*J_ortho_* = 8.2 Hz), 6.64 (d, 1H, ^3^*J_cis_* = 10.3 Hz), 6.27 (d, 1H, ^3^*J_cis_* = 9.5 Hz), 4.84 (d, 1H, ^3^*J_cis_* = 10.3 Hz), 4.71 (d, 1H, ^3^*J_cis_* = 9.5 Hz), 4.63 (s, 1H), 4.52 (s, 1H), 3.66–3.49 (m, 3H), 3.48–3.38 (m, 1H), 2.55–2.43 (m, 2H), 2.42 (s, 3H), 2.41 (s, 3H), 2.20–1.93 (m, 6H), 1.66–1.56 (m, 1H), 1.55–1.46 (m, 1H). ^13^C{^1^H} NMR (CDCl_3_, 101 MHz): δ 149.3, 143.9, 143.7, 136.2, 135.8, 132.9, 130.0, 129.8, 127.2, 127.1, 125.8, 125.7, 124.7, 122.6, 111.8, 110.6, 50.1, 47.0, 44.6, 42.5, 36.7, 34.7, 32.7, 29.1, 21.7, 21.6. HRMS (ESI) *m*/*z*: [M + Na]^+^ calcd. for C_30_H_34_N_2_O_4_S_2_Na: 573.1852; found: 573.1836; 1 mmol scale: compound **2a** was obtained from bisallene **1a** (275 mg, 1 mmol) following the general procedure. Purification by column chromatography (silica gel, 40–63 μm, hexanes/EtOAc 90:10 to 60:40 v/v) provided **2a** (230 mg, 84% yield) as a colorless solid.

### Spiro Derivative **2b**

Compound **2b** was obtained from bisallene **1b** (26.5 mg, 0.09 mmol) following the general procedure. Purification by column chromatography (silica gel, 40–63 μm, hexanes/EtOAc: 90:10 to 60:40 v/v) provided **2b** (23.4 mg, 89% yield) as a colorless oil. IR (ATR) ν (cm^–1^): 2921, 1339, 1153. ^1^H NMR (CDCl_3_, 400 MHz): δ 7.74–7.68 (m, 4H), 7.02–6.91 (m, 4H), 6.64 (d, 1H, ^3^*J_cis_* = 10.3 Hz), 6.27 (d, 1H, ^3^*J_cis_* = 9.5 Hz), 4.83 (d, 1H, ^3^*J_cis_* = 10.3 Hz), 4.70 (d, 1H, ^3^*J_cis_* = 9.5 Hz), 4.64 (s, 1H), 4.53 (s, 1H), 3.87 (s, 3H), 3.86 (s, 3H), 3.66–3.49 (m, 3H), 3.47–3.39 (m, 1H), 2.55–2.38 (m, 2H), 2.20–1.94 (m, 6H), 1.66–1.58 (m, 1H), 1.55–1.49 (m, 1H). ^13^C{^1^H} NMR (CDCl_3_, 101 MHz): δ 163.2, 163.1, 149.4, 132.9, 130.9, 130.4, 129.3, 129.2, 125.9, 125.7, 124.8, 122.5, 114.5, 114.3, 111.8, 110.5, 55.8 (×2), 50.0, 47.0, 44.6, 42.5, 36.7, 34.8, 32.7, 29.1. HRMS (ESI) *m*/*z*: [M + Na]^+^ calcd. for C_30_H_34_N_2_O_6_S_2_Na: 605.1750; found: 605.1767.

### Spiro Derivative **2c**

Compound **2c** was obtained from bisallene **1c** (29.8 mg, 0.09 mmol) following the general procedure. Purification by column chromatography (silica gel, 40–63 μm, hexanes/EtOAc: 90:10 to 60:40 v/v) provided **2c** (26.0 mg, 87% yield) as colorless oil. IR (ATR) ν (cm^–1^): 2922, 1351, 1305, 1162. ^1^H NMR (CDCl_3_, 400 MHz): δ 8.06–8.00 (m, 2H), 7.94–7.86 (m, 2H), 7.75–7.64 (m, 4H), 6.60 (d, 1H, ^3^*J_cis_* = 10.2 Hz), 6.32 (d, 1H, ^3^*J_cis_* = 9.3 Hz), 4.90 (d, 1H, ^3^*J_cis_* = 10.2 Hz), 4.84 (d, 1H, ^3^*J_cis_* = 9.3 Hz), 4.66 (s, 1H), 4.62 (s, 1H), 3.79–3.69 (m, 1H), 3.68–3.57 (m, 3H), 2.59–2.41 (m, 2H), 2.30–2.01 (m, 6H), 1.73–1.65 (m, 1H), 1.65–1.57 (m, 1H). ^13^C{^1^H} NMR (CDCl_3_, 101 MHz): δ 149.0, 138.8 (q, ^4^*J_C-F_* = 1.2 Hz), 138.2 (q, ^4^*J_C-F_* = 1.2 Hz), 133.1, 133.0, 132.8, 132.5 (q, ^4^*J_C-F_* = 0.9 Hz), 132.3 (q, ^4^*J_C-F_* = 0.9 Hz), 131.3, 131.2, 128.8 (q, ^3^*J_C-F_* = 6.4 Hz), 128.6 (q, ^3^*J_C-F_* = 6.3 Hz), 128.3 (q, ^2^*J_C-F_* = 33.4 Hz), 128.1 (q, ^2^*J_C-F_* = 33.4 Hz), 125.7, 125.6, 124.5, 123.7, 122.5 (q, ^1^*J_C-F_* = 274.2 Hz), 122.4 (q, ^1^*J_C-F_* = 274.3 Hz), 112.3, 110.8, 50.4, 47.3, 44.5, 42.6, 37.2, 34.7, 32.6, 29.2. HRMS (ESI) *m*/*z*: [M + Na]^+^ calcd. for C_30_H_28_F_6_N_2_O_4_S_2_Na: 681.1287; found: 681.1282.

### Spiro Derivative **2d**

Compound **2d** was obtained from bisallene **1d** (25.3 mg, 0.09 mmol) following the general procedure. Purification by column chromatography (silica gel, 40–63 μm, hexanes/EtOAc: 90:10 to 60:40 v/v) provided **2d** (19.0 mg, 75% yield) as colorless oil. IR (ATR) ν (cm^–1^): 2921, 1341, 1168. ^1^H NMR (CDCl_3_, 400 MHz): δ 8.55–8.48 (m, 2H), 7.88–7.79 (m, 2H), 7.72–7.64 (m, 2H), 6.59 (d, 1H, ^3^*J_cis_* = 10.3 Hz), 6.34 (d, 1H, ^3^*J_cis_* = 9.4 Hz), 4.83 (d, 1H, ^3^*J_cis_* = 10.3 Hz), 4.76 (d, 1H, ^3^*J_cis_* = 9.4 Hz), 4.69 (s, 1H), 4.60 (s, 1H), 3.82–3.64 (m, 3H), 3.64–3.54 (m, 1H), 2.64–2.48 (m, 2H), 2.43 (s, 6H), 2.30–2.20 (m, 3H), 2.15–1.93 (m, 3H), 1.74–1.65 (m, 1H), 1.62–1.52 (m, 1H). ^13^C{^1^H} NMR (CDCl_3_, 101 MHz): δ 154.3, 154.0, 150.9, 150.7, 149.2, 138.3, 138.1, 137.6, 137.4, 133.1, 126.6, 125.5, 124.9, 123.1, 122.3, 122.2, 111.9, 110.8, 50.9, 47.8, 44.5, 42.4, 37.4, 35.2, 32.5, 29.2, 18.7, 18.6. HRMS (ESI) *m*/*z*: [M + Na]^+^ calcd. for C_28_H_32_N_4_O_4_S_2_Na: 575.1757; found: 575.1766.

### Spiro Derivative **2e**

Compound **2e** was obtained from bisallene **1e** (21.8 mg, 0.09 mmol) following the general procedure. Purification by column chromatography (silica gel, 40–63 μm, hexanes/EtOAc: 90:10 to 60:40 v/v) provided **2e** (14.8 mg, 68% yield) as colorless oil. IR (ATR) ν (cm^–1^): 2921, 1319, 1128. ^1^H NMR (CDCl_3_, 400 MHz): δ 6.49 (d, 1H, ^3^*J_cis_* = 10.3 Hz), 6.41 (d, 1H, ^3^*J_cis_* = 9.7 Hz), 4.91 (s, 1H), 4.80 (s, 1H), 4.76 (d, 1H, ^3^*J_cis_* = 10.3 Hz), 4.63 (d, 1H, ^3^*J_cis_* = 9.7 Hz), 3.85–3.72 (m, 4H), 2.69–2.54 (m, 2H), 2.50–2.43 (m, 2H), 2.41–2.32 (m, 1H), 2.25–2.06 (m, 3H), 1.82–1.66 (m, 2H), 1.41 (s, 9H), 1.39 (s, 9H). ^13^C{^1^H} NMR (CDCl_3_, 101 MHz): δ 149.8, 132.2, 128.4, 126.9, 125.9, 118.9, 112.3, 108.1, 62.9, 62.4, 51.0, 49.1, 44.9, 42.7, 38.4, 35.3, 33.4, 29.1, 24.9, 24.7. HRMS (ESI) *m*/*z*: [M + Na]^+^ calcd. for C_24_H_38_N_2_O_4_S_2_Na: 505.2165; found: 505.2177.

### Spiro Derivative **2f**

Compound **2f** was obtained from bisallene **1f** (25.9 mg, 0.09 mmol) following the general procedure. Purification by column chromatography (silica gel, 40–63 μm, hexanes/EtOAc: 90:10 to 60:40 v/v) provided **2f** (14.2 mg, 55% yield) as colorless oil. IR (ATR) ν (cm^–1^): 2949, 1335, 1142. ^1^H NMR (CDCl_3_, 400 MHz): δ 6.49 (d, 1H, ^3^*J_cis_* = 10.2 Hz), 6.29 (d, 1H, ^3^*J_cis_* = 9.4 Hz), 4.91 (s, 1H), 4.83 (d, 1H, ^3^*J_cis_* = 10.2 Hz), 4.79 (s, 1H), 4.75 (d, 1H, ^3^*J_cis_* = 9.4 Hz), 3.80–3.63 (m, 4H), 3.01–2.89 (m, 4H), 2.70–2.54 (m, 2H), 2.49–2.33 (m, 3H), 2.27–2.07 (m, 3H), 1.85–1.67 (m, 2H), 1.06–0.98 (m, 4H), 0.05 (s, 9H), 0.04 (s, 9H). ^13^C{^1^H} NMR (CDCl_3_, 101 MHz): δ 149.6, 132.2, 126.6, 125.9, 125.5, 121.4, 112.3, 109.4, 49.9, 49.1, 49.0, 47.2, 44.7, 42.7, 38.1, 35.4, 33.0, 29.2, 10.4 (×2), −1.8, −1.9. HRMS (ESI) *m*/*z*: [M + Na]^+^ calcd. for C_26_H_46_N_2_O_4_S_2_Si_2_Na: 593.2330; found: 593.2340.

### Spiro Derivative **2g**

Compound **2 g** was obtained from bisallene **1g** (23.8 mg, 0.09 mmol) following the general procedure. Purification by column chromatography (silica gel, 40–63 μm, hexanes/EtOAc: 90:10 to 60:40 v/v) provided **2g** (19.0 mg, 80% yield) as colorless oil. IR (ATR) ν (cm^–1^): 2929, 1725, 1224. ^1^H NMR (CDCl_3_, 400 MHz): δ 5.85 (d, 1H, ^3^*J_cis_* = 12.4 Hz), 5.80 (d, 1H, ^3^*J_cis_* = 12.4 Hz), 5.70 (d, 1H, ^3^*J_cis_* = 12.1 Hz), 5.57 (d, 1H, ^3^*J_cis_* = 12.1 Hz), 4.81 (s, 1H), 4.69 (s, 1H), 4.23–4.11 (m, 8H), 2.62–2.44 (m, 2H), 2.44–2.25 (m, 5H), 2.25–1.98 (m, 5H), 1.77–1.59 (m, 2H), 1.30–1.20 (m, 12H). ^13^C{^1^H} NMR (CDCl_3_, 101 MHz): δ 171.5, 171.2, 171.1, 171.0, 149.9, 139.8, 138.0, 132.8, 125.8, 125.7, 125.5, 112.5, 61.8 (×2), 61.7 (×2), 61.5, 58.6, 43.9, 43.8, 33.5, 32.7, 32.0, 31.7, 30.8, 28.8, 14.2 (×2), 14.1 (×2). HRMS (ESI) *m*/*z*: [M + Na]^+^ calcd. for C_30_H_40_O_8_Na: 551.2615; found: 551.2615.

### Spiro Derivative **2h**

Compound **2h** was obtained from bisallene **1h** (19.7 mg, 0.09 mmol) following the general procedure. Purification by column chromatography (silica gel, 40–63 μm, hexanes/EtOAc: 90:10 to 60:40 v/v) provided **2h** (8.3 mg, 42% yield) as colorless oil. IR (ATR) ν (cm^–1^): 2973, 1697, 1649, 1451, 1431. ^1^H NMR (CDCl_3_, 400 MHz): 6.89–6.56 (bs, 1H), 6.55–6.24 (bs, 1H), 4.86 (s, 1H), 4.84–4.71 (bs, 1H), 4.73 (s, 1H), 4.72–4.59 (bs, 1H), 3.81–13.59 (m, 4H), 2.55 (bs, 2H), 2.42–2.29 (m, 3H), 2.25–2.05 (m, 3H), 1.85–1.71 (bs, 1H), 1.71–1.61 (m, 1H), 1.48 (s, 9H), 1.46 (s, 9H). ^13^C{^1^H} NMR (CDCl_3_, 101 MHz): δ 153.7, 152.5, 150.4, 132.9, 127.6, 126.1, 125.4, 120.5, 111.3, 109.3, 81.3, 80.7, 47.6, 44.7, 44.4, 42.5, 37.7, 34.7, 33.0, 29.5, 28.4 (×2). HRMS (ESI) *m*/*z*: [M + Na]^+^ calcd. for C_26_H_38_N_2_O_4_Na: 465.2724; found: 465.2718; 1 mmol scale: compound **2h** was obtained from bisallene **1h** (221 mg, 1 mmol) following the general procedure. Purification by column chromatography (silica gel, 40–63 μm, hexanes/EtOAc: 90:10 to 60:40 v/v) provided **2h** (92.4 mg, 42% yield) as a colorless solid.

## Supporting Information Available

Experimental procedures, compound characterization data including NMR spectra for all new compounds, and computational data (PDF)
