# Supplementary material for: Highly Selective Synthesis of Seven-Membered Azaspiro Compounds by a Rh(I)-Catalyzed Cycloisomerization/Diels–Alder Cascade of 1,5-Bisallenes
Source: J Org Chem. 2022 Mar 24;87(8):5279–86. doi: 10.1021/acs.joc.2c00065 (PMC9016767; doi:10.1021/acs.joc.2c00065)
Supplement: Supplementary file 1 — jo2c00065_si_001.pdf [file jo2c00065_si_001.pdf]

SUPPORTING INFORMATION

Highly selective synthesis of 7-membered azaspiro compounds by a Rh(I)-catalyzed cycloisomerization / Diels-Alder cascade of 1,5-bisallenes.

Jordi Vila, Miquel Solà, Anna Pla-Quintana, Anna Roglans*.*

Institut de Química Computacional i Catàlisi (IQCC) and Departament de Química,

Universitat de Girona (UdG), Facultat de Ciències, C/ Maria Aurèlia Capmany, 69, 17003-

Girona, Catalunya, Spain.

TABLE OF CONTENTS

- General materials and methods....S3
- Synthesis of bisallene 1h....S4

Scheme S1. Synthesis of N-Boc bisalkyne S1h and bisallene 1h....S4

- Synthesis of compounds 2a-2h....S5

Scheme S2. General procedure GP1 for the synthesis of products 2a-2h....S5

- Identification of the single regioisomer 2A (product 2a)....S10

Figure S1. All possible chemo- and regioisomers of 2a....S10

Figure S2. COSY and HMBC crosspeaks, and NOE contacts observed in 2a confirming the formation of cycloadduct 2A....S10

Figure S3. HSQC (blue/red) + HMBC (green) combined spectra....S11

Figure S4. COSY (red) + NOESY (blue) combined spectra....S11

- Detection of the biradical intermediate using ESI(+)-MS....S12

Scheme S3. Paths leading to the detected species containing TEMPO....S12

Figure S5. ESI-MS detection of $[int(2A)+2TEMPO+H]^+$ (A)....S12

Figure S6. ESI-MS detection of $[int(2A)+TEMPO]^+$ (B) and $[int(2A)H+TEMPO+H]^+$ (C)....S13

- Computational methods....S14
- Figure S7. Transition state Gibbs energy barrier of all reaction paths computed at 313.15K and 1 atm with the (U)B3LYP-D3/cc-pVTZ/SMD(76% THF, 24% CH_2Cl_2)//(U)B3LYP-D3/cc-pVDZ....S15
- Figure S8. Molecular structures of (a) $TS^{cs}(1A_{exo})$, (b) $TS^{cs}(1A_{endo})$, (c) $TS^{cs}(1B_{exo})$, (d) $TS^{cs}(1B_{endo})$, (e) $TS^{cs}(2A_{exo})$, (f) $TS^{cs}(2A_{endo})$, (g) $TS^{cs}(2B_{exo})$, (h) $TS^{cs}(2B_{endo})$, (i) $TS^{cs}(3A_{exo})$, (j) $TS^{cs}(3A_{endo})$, (k) $TS^{cs}(3B_{exo})$, (l) $TS^{cs}(3B_{endo})$, (m) $TS^{os}(2A_{exo})$, (n) $TS^{os}(2A_{endo})$, (o) $TS^{os}(int(2A_{exo})$, (p) $TS^{os}(int(2A_{endo}))$. Distances are given in Angstroms (Å)....S16
- Table S1. Closed-shell singlet, Open-shell singlet and Open-shell triplet energy comparisson of all reaction pathways computed at 313.15K and 1 atm with the (U)B3LYP-D3/cc-pVTZ/SMD(76% THF, 24% CH_2Cl_2)//(U)B3LYP-D3/cc-pVDZ....S17
- Figure S9. Frontier orbitals HOMO for unpaired electrons α and β of (a) $int(2A_{exo})$, and (b) $int(2A_{endo})$. Rendered at 0.026 isosurface contour values....S17
- Computational data....S18
- ^{1}H and ^{13}C NMR spectra....S19

Bisallene 1h....S19

Product 2a....S21

Product 2b....S30

Product 2c....S33

Product 2d....S37

Product 2e....S40

Product 2f....S43

Product 2g....S46

Product 2h....S49

- References....S55

General materials and methods

Unless otherwise noted, materials were obtained from commercial suppliers and used without further purification. Bisallenes **1a** (X = NTs), **1b** (X = *p*-MeO-$PhSO_2N$), **1c** (X = *o*-CF_3-$PhSO_2N$), **1d** (X = 5-methyl-2-pyridinesulfonamidil), **1e** (X = ^{t}Bu-SO_2N), **1f** (X = TMS-CH_2CH_2-SO_2N) and **1g** (X = $C(COOEt)_2$) were prepared from the corresponding bisalkynes using Crabbé homologation reaction. Experimental procedures and full characterization has been described by us in a previous publication.[1] Bisallene **1h** (X = NBoc) was prepared from the corresponding bisalkyne[2] using Crabbé homologation reaction.

CH_2Cl_2 and THF were dried under nitrogen by passing through solvent purification columns (MBraun, SPS-800). Reaction progress during the preparation of all compounds was monitored using thin layer chromatography on Macherey-Nagel Xtra SIL G/UV254 silica gel plates. Solvents were removed under reduced pressure with a rotary evaporator. Reaction mixtures were chromatographed on silica gel using an automated purification instrument Interchim PuriFlash XS 520 Plus equipped with a quaternary gradient pump (up to 300 ml/min, 20 bar) and an UV-Vis 200-800 nm díode array detector. All ^{1}H and ^{13}C NMR spectra were recorded on a Bruker ASCEND 400 spectrometer equipped with a 5 mm BBFO probe using $CDCl_3$ as a deuterated solvent. Chemical shifts for ^{1}H and ^{13}C NMR are reported in ppm (δ) relative to residual solvent signals (7.26 ppm for ^{1}H, 77,16 ppm for ^{13}C). Coupling constants are given in Hertz (Hz). ^{1}H and ^{13}C NMR signals were assigned based on 2D-NMR HSQC, HMBC, COSY and NOESY experiments. Electrospray mass spectrometry analyses were recorded on an Esquire 6000 ion trap mass spectrometer (Bruker) equipped with an electrospray ion source. Electrospray ionisation high-resolution mass spectrometry was performed using a Bruker microTOF-Q II instrument. Both mass instruments were operated in the positive ESI(+) ion mode. IR spectra were recorded on an Agilent Cary 630 FT-IR spectrometer equipped with an ATR sampling accessory. Melting points were measured in a SMP10 apparatus from Stuart without any correction.

Synthesis of bisallene 1h

Scheme S1. Synthesis of N-Boc bisalkyne **S1h** and bisallene **1h**

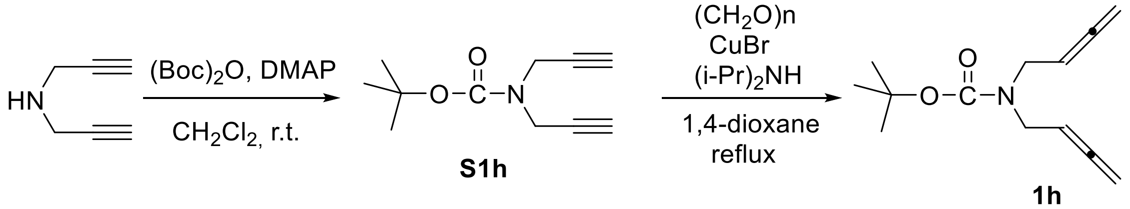

In a 100 mL round-bottom flask equipped with a magnetic stirrer, a mixture of dipropargylamine (0.97 g, 10.41 mmol,1 equiv.) and DMAP (0.13 g, 1.06 mmol, 0.1 equiv.) in CH_2Cl_2 (30 mL) was stirred at 0°C. A solution of $(Boc)_2O$ (3.02 g, 13.84 mmol, 1.3 equiv.) in CH_2Cl_2 was then added at 0ºC for 15 minutes and the resulting mixture was stirred at room temperature overnight. The reaction mixture was concentrated under reduced pressure and the resulting crude was purified by column chromatography on silica gel (silica gel, 40–63 µm) using mixtures of hexane/EtOAc as the eluent (100:0 to 80:20 v/v) to afford diyne **S1h** (2.02 g, quantitative yield) as a yellow oil.[2]

MW ($C_{11}H_{15}NO_2$): 193.25 g/mol; **Rf**: 0.72 (Hexane/EtOAc 8:2); **^{1}H NMR ($CDCl_3$, 400 MHz):** δ 4.17 (bs, 4H), 2.22 (t, 2H, ^4J = 2.5 Hz), 1.48 (s, 9H). **^{13}C{^{1}H} NMR ($CDCl_3$, 101 MHz):** δ 154.4, 81.3, 78.9, 72.0, 35.3, 28.4. **ESI-MS (m/z):** 216.1 $[M+Na]^+$.

In a round-bottom flask equipped with a reflux condenser and a magnetic stirrer, a suspension of the diyine **S1h** (2.02 g, 10.45 mmol, 1 equiv.), paraformaldehyde (1.65 g, 54.94 mmol, 5.2 equiv.) and CuBr (1.5 g, 10.45 mmol, 1 equiv.) in 1,4-dioxane (35 mL) was stirred and heated to reflux. Diisopropylamine (6 mL, 42.81 mmol, 4 equiv.) was then added and the resulting mixture was stirred at reflux for 3 h until completion (TLC monitoring). The reaction mixture was allowed to cool to room temperature, filtered through a Celite pad (on top) and silica gel (on bottom) and concentrated under reduced pressure. The resulting crude was purified by column chromatography (SiO_2, 40–60 µm) using mixtures of hexane/EtOAc as the eluent (100:0 to 80:20 v/v) to afford bisallene **1h** (1.59 g, 69% yield) as a yellowish oil.

MW ($C_{13}H_{19}NO_2$): 221.30 g/mol; **Rf**: 0.81 (Hexane/EtOAc 8:2); **IR (ATR) ν (cm^{-1})**: 2974, 1953, 1691, 1454. **^{1}H NMR ($CDCl_3$, 400 MHz):** δ 5.11 (quint, 2H, ^3J = ^4J = 6.5 Hz), 4.76 (dt, 4H, ^4J = 6.5 Hz, ^5J = 2.8 Hz), 3.85 (bs, 4H), 1.46 (s, 9H). **^{13}C{^{1}H} NMR ($CDCl_3$, 101 MHz):** δ 209.1, 155.3, 87.3, 79.9, 76.2, 45.4, 28.6. **HRMS (ESI) m/z:** $[M+Na]^+$ Calcd. for $C_{13}H_{19}NO_2Na$ 244.1308; Found 244.1314.

Synthesis of compounds 2a-2h

Scheme S2. General procedure **GP1** for the synthesis of products **2a-2h**

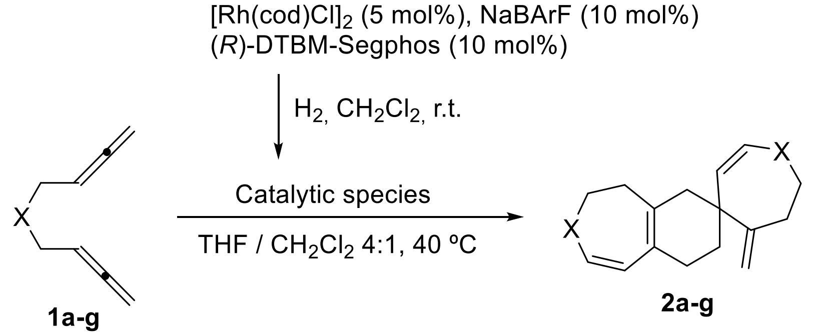

1a-g **2a-g**

In a 10 mL capped vial, a mixture of $[Rh(cod)Cl]_2$ (2.2 mg, 0.004 mmol, 0.05 equiv.), (*R*)-DTBM-Segphos (11.3 mg, 0.01 mmol, 0.10 equiv.) and NaBArF (8.5 mg, 0.01 mmol, 0.10 equiv.) was purged with nitrogen and dissolved in anhydrous CH_2Cl_2 (4 mL). Hydrogen gas was bubbled into the catalyst solution and the mixture was stirred for 30 min. The mixture was then concentrated to dryness under a stream of hydrogen, dissolved again in anhydrous CH_2Cl_2 (0.5 mL) and transferred via syringe into a solution of bisallene **1a-h** (0.09 mmol, 1 equiv.) in anhydrous THF (2 mL) under inert atmosphere at 40 °C (aluminium heating block). The resulting mixture was stirred for 16h at 40 °C. The solvent was removed under reduced pressure and the crude reaction mixture was purified by column chromatography on silica gel using mixtures of hexane/EtOAc as the eluent (90:10 to 60:40 v/v).

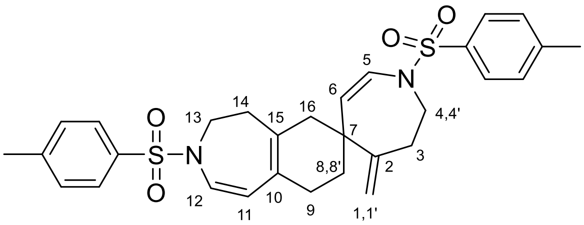

2a

Compound **2a** was obtained from bisallene **1a** (25 mg, 0.09 mmol) following the general procedure **GP1**. Purification by column chromatography (silica gel, 40–63 µm, Hexanes/EtOAc 90:10 to 60:40 v/v) provided **2a** (22.3 mg, 89% yield) as a colourless solid.

MW ($C_{30}H_{34}N_2O_4S_2$): 550.73 g/mol; **Rf**: 0.26 (Hexane/EtOAc 8:2); **MP (°C):** 87-92 (dec); **IR (ATR) *ν* (cm^{-1})**: 2921, 1336, 1156. **^{1}H NMR ($CDCl_3$, 400 MHz):** δ 7.66 (m, 4H, CH-**Ar**), 7.31 (d, 2H, $^3J_{ortho}$ = 8.2 Hz, CH-**Ar**), 7.28 (d, 2H, $^3J_{ortho}$ = 8.2 Hz, CH-**Ar**), 6.64 (d, 1H, $^3J_{cis}$ = 10.3 Hz, H**12**), 6.27 (d, 1H, $^3J_{cis}$ = 9.5 Hz, H**5**), 4.84 (d, 1H, $^3J_{cis}$ = 10.3 Hz, H**11**), 4.71 (d, 1H, $^3J_{cis}$ = 9.5 Hz, H**6**), 4.63 (s, 1H, H**1**/H**1'**), 4.52 (s, 1H, H**1**/H**1'**), 3.66 - 3.49 (m, 3H, H**4**/H**4'**, H**13**), 3.48 - 3.38 (m, 1H, H**4**/H**4'**), 2.55 - 2.43 (m, 2H, H**3**), 2.42 (s, 3H, CH_3-**Ar**), 2.41 (s, 3H, CH_3-**Ar**), 2.20 – 1.93 (m, 6H, H**9**, H**14**, H**16**), 1.66 - 1.56 (m, 1H, H**8**/H**8'**), 1.55 - 1.46 (m, 1H, H**8**/H**8'**). **^{13}C{^{1}H} NMR ($CDCl_3$, 101 MHz):** δ 149.3 (C**2**), 143.9 (C-**Ar**), 143.7 (C-**Ar**), 136.2 (C-**Ar**), 135.8 (C-**Ar**), 132.9 (C**15**), 130.0 (CH-**Ar**), 129.8 (CH-**Ar**), 127.2 (CH-**Ar**), 127.1 (CH-**Ar**), 125.8 (C**10**), 125.7 (C**5**), 124.7 (C**12**), 122.6

(C**6**), 111.8 (C**1**), 110.6 (C**11**), 50.1 (C**4**), 47.0 (C**13**), 44.6 (C**16**), 42.5 (C**7**), 36.7 (C**14**), 34.7 (C**3**), 32.7 (C**8**), 29.1 (C**9**), 21.7 (CH_3-**Ar**), 21.6 (CH_3-**Ar**). **HRMS (ESI) m/z:** $[M+Na]^+$ Calcd. for $C_{30}H_{34}N_2O_4S_2Na$ 573.1852; Found 573.1836.

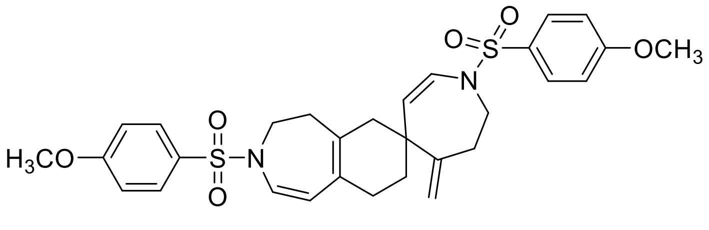

2b

Compound **2b** was obtained from bisallene **1b** (26.5 mg, 0.09 mmol) following the general procedure **GP1**. Purification by column chromatography (silica gel, 40–63 μm, Hexanes/EtOAc 90:10 to 60:40 v/v) provided **2b** (23.4 mg, 89% yield) as a colourless oil.

MW ($C_{30}H_{34}N_2O_6S_2$): 582.73 g/mol; **Rf**: 0.28 (Hexane/EtOAc 7:3); **IR (ATR) ν (cm⁻¹)**: 2921, 1339, 1153. **^{1}H NMR ($CDCl_3$, 400 MHz):** δ 7.74 – 7.68 (m, 4H), 7.02 – 6.91 (m, 4H), 6.64 (d, 1H, $^3J_{cis}$ = 10.3 Hz), 6.27 (d, 1H, $^3J_{cis}$ = 9.5 Hz), 4.83 (d, 1H, $^3J_{cis}$ = 10.3 Hz), 4.70 (d, 1H, $^3J_{cis}$ = 9.5 Hz), 4.64 (s, 1H), 4.53 (s, 1H), 3.87 (s, 3H), 3.86 (s, 3H), 3.66 - 3.49 (m, 3H), 3.47 - 3.39 (m, 1H), 2.55 - 2.38 (m, 2H), 2.20 – 1.94 (m, 6H), 1.66 – 1.58 (m, 1H), 1.55 – 1.49 (m, 1H). **^{13}C{^{1}H} NMR ($CDCl_3$, 101 MHz):** δ 163.2, 163.1, 149.4, 132.9, 130.9, 130.4, 129.3, 129.2, 125.9, 125.7, 124.8, 122.5, 114.5, 114.3, 111.8, 110.5, 55.8 (2x), 50.0, 47.0, 44.6, 42.5, 36.7, 34.8, 32.7, 29.1. **HRMS (ESI) m/z:** $[M+Na]^+$ Calcd. for $C_{30}H_{34}N_2O_6S_2Na$ 605.1750; Found 605.1767.

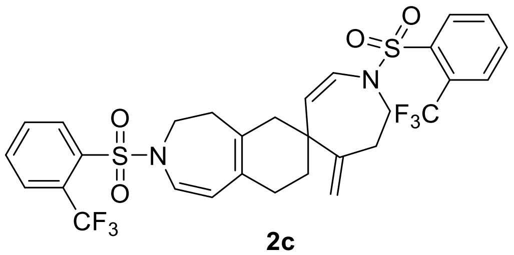

2c

Compound **2c** was obtained from bisallene **1c** (29.8 mg, 0.09 mmol) following the general procedure **GP1**. Purification by column chromatography (silica gel, 40–63 μm, Hexanes/EtOAc 90:10 to 60:40 v/v) provided **2c** (26.0 mg, 87% yield) as a colourless oil.

MW ($C_{30}H_{28}F_6N_2O_4S_2$): 658.67 g/mol; **Rf**: 0.32 (Hexane/EtOAc 7:3); **IR (ATR) ν (cm⁻¹)**: 2922, 1351, 1305, 1162. **^{1}H NMR ($CDCl_3$, 400 MHz):** δ 8.06 – 8.00 (m, 2H), 7.94 – 7.86 (m, 2H), 7.75 – 7.64 (m, 4H), 6.60 (d, 1H, $^3J_{cis}$ = 10.2 Hz), 6.32 (d, 1H, $^3J_{cis}$ = 9.3 Hz), 4.90 (d, 1H, $^3J_{cis}$ = 10.2 Hz), 4.84 (d, 1H, $^3J_{cis}$ = 9.3 Hz), 4.66 (s, 1H), 4.62 (s, 1H), 3.79 - 3.69 (m, 1H), 3.68 - 3.57 (m, 3H), 2.59 - 2.41 (m, 2H), 2.30 – 2.01 (m, 6H), 1.73 – 1.65 (m, 1H), 1.65 – 1.57 (m, 1H). **^{13}C{^{1}H} NMR ($CDCl_3$, 101 MHz):** δ 149.0, 138.8 (q, $^4J_{C-F}$ = 1.2 Hz), 138.2 (q, $^4J_{C-F}$ = 1.2 Hz), 133.1, 133.0, 132.8, 132.5 (q, $^4J_{C-F}$ = 0.9 Hz), 132.3 (q, $^4J_{C-F}$ = 0.9 Hz), 131.3, 131.2, 128.8 (q, $^3J_{C-F}$ = 6.4 Hz), 128.6 (q, $^3J_{C-F}$ = 6.3 Hz), 128.3 (q, $^2J_{C-F}$ = 33.4 Hz), 128.1 (q, $^2J_{C-F}$ = 33.4 Hz), 125.7, 125.6, 124.5, 123.7, 122.5

(q, $^{1}J_{C\text{-}F}$ = 274.2 Hz), 122.4 (q, $^{1}J_{C\text{-}F}$ = 274.3 Hz), 112.3, 110.8, 50.4, 47.3, 44.5, 42.6, 37.2, 34.7, 32.6, 29.2. **HRMS (ESI) m/z:** $[M+Na]^+$ Calcd. for $C_{30}H_{28}F_6N_2O_4S_2Na$ 681.1287; Found 681.1282.

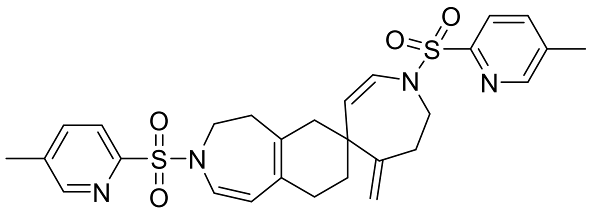

2d

Compound **2d** was obtained from bisallene **1d** (25.3 mg, 0.09 mmol) following the general procedure **GP1**. Purification by column chromatography (silica gel, 40–63 μm, Hexanes/EtOAc 90:10 to 60:40 v/v) provided **2d** (19.0 mg, 75% yield) as a colourless oil.

MW ($C_{28}H_{32}N_4O_4S_2$): 552.71 g/mol; **Rf**: 0.15 (Hexane/EtOAc 7:3); **IR (ATR) *ν* (cm^{-1})**: 2921, 1341, 1168. **^{1}H NMR (CDCl$_3$, 400 MHz):** δ 8.55 – 8.48 (m, 2H), 7.88 – 7.79 (m, 2H), 7.72 -7.64 (m, 2H), 6.59 (d, 1H, $^{3}J_{cis}$ = 10.3 Hz), 6.34 (d, 1H, $^{3}J_{cis}$ = 9.4 Hz), 4.83 (d, 1H, $^{3}J_{cis}$ = 10.3 Hz), 4.76 (d, 1H, $^{3}J_{cis}$ = 9.4 Hz), 4.69 (s, 1H), 4.60 (s, 1H), 3.82 – 3.64 (m, 3H), 3.64 – 3.54 (m, 1H), 2.64 – 2.48 (m, 2H), 2.43 (s, 6H), 2.30 – 2.20 (m, 3H), 2.15 – 1.93 (m, 3H), 1.74 – 1.65 (m, 1H), 1.62 – 1.52 (m, 1H). **^{13}C{^{1}H} NMR (CDCl$_3$, 101 MHz):** δ 154.3, 154.0, 150.9, 150.7, 149.2, 138.3, 138.1, 137.6, 137.4, 133.1, 126.6, 125.5, 124.9, 123.1, 122.3, 122.2, 111.9, 110.8, 50.9, 47.8, 44.5, 42.4, 37.4, 35.2, 32.5, 29.2, 18.7, 18.6. **HRMS (ESI) m/z:** $[M+Na]^+$ Calcd. for $C_{28}H_{32}N_4O_4S_2Na$ 575.1757; Found 575.1766.

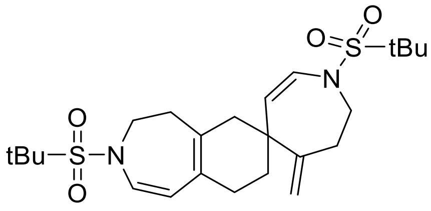

2e

Compound **2e** was obtained from bisallene **1e** (21.8 mg, 0.09 mmol) following the general procedure **GP1**. Purification by column chromatography (silica gel, 40–63 μm, Hexanes/EtOAc 90:10 to 60:40 v/v) provided **2e** (14.8 mg, 68% yield) as a colourless oil.

MW ($C_{24}H_{38}N_2O_4S_2$): 482.70 g/mol; **Rf**: 0.32 (Hexane/EtOAc 8:2); **IR (ATR) *ν* (cm^{-1})**: 2921, 1319, 1128. **^{1}H NMR (CDCl$_3$, 400 MHz):** δ 6.49 (d, 1H, $^{3}J_{cis}$ = 10.3 Hz), 6.41 (d, 1H, $^{3}J_{cis}$ = 9.7 Hz), 4.91 (s, 1H), 4.80 (s, 1H), 4.76 (d, 1H, $^{3}J_{cis}$ = 10.3 Hz), 4.63 (d, 1H, $^{3}J_{cis}$ = 9.7 Hz), 3.85 – 3.72 (m, 4H), 2.69 – 2.54 (m, 2H), 2.50 – 2.43 (m, 2H), 2.41 – 2.32 (m, 1H), 2.25 – 2.06 (m, 3H), 1.82 – 1.66 (m, 2H), 1.41 (s, 9H), 1.39 (s, 9H). **^{13}C{^{1}H} NMR (CDCl$_3$, 101 MHz):** δ 149.8, 132.2, 128.4, 126.9, 125.9, 118.9, 112.3, 108.1, 62.9, 62.4, 51.0, 49.1, 44.9, 42.7, 38.4, 35.3, 33.4, 29.1, 24.9, 24.7. **HRMS (ESI) m/z:** $[M+Na]^+$ Calcd. for $C_{24}H_{38}N_2O_4S_2Na$ 505.2165; Found 505.2177.

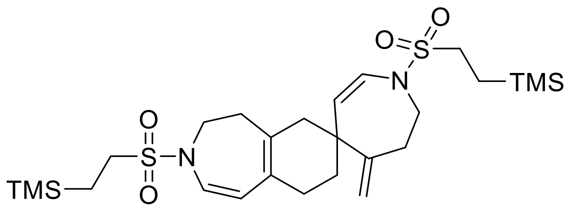

2f

Compound **2f** was obtained from bisallene **1f** (25.9 mg, 0.09 mmol) following the general procedure **GP1**. Purification by column chromatography (silica gel, 40–63 µm, Hexanes/EtOAc 90:10 to 60:40 v/v) provided **2f** (14.2 mg, 55% yield) as a colourless oil.

MW ($C_{26}H_{46}N_2O_4S_2Si_2$): 570.95 g/mol; **Rf**: 0.46 (Hexane/EtOAc 8:2); **IR (ATR) *ν* (cm^{-1})**: 2949, 1335, 1142. **^{1}H NMR (CDCl$_3$, 400 MHz):** δ 6.49 (d, 1H, $^3J_{cis}$ = 10.2 Hz), 6.29 (d, 1H, $^3J_{cis}$ = 9.4 Hz), 4.91 (s, 1H), 4.83 (d, 1H, $^3J_{cis}$ = 10.2 Hz), 4.79 (s, 1H), 4.75 (d, 1H, $^3J_{cis}$ = 9.4 Hz), 3.80 – 3.63 (m, 4H), 3.01 – 2.89 (m, 4H), 2.70 – 2.54 (m, 2H), 2.49 – 2.33 (m, 3H), 2.27 – 2.07 (m, 3H), 1.85 – 1.67 (m, 2H), 1.06 – 0.98 (m, 4H), 0.05 (s, 9H), 0.04 (s, 9H). **^{13}C{^{1}H} NMR (CDCl$_3$, 101 MHz):** δ 149.6, 132.2, 126.6, 125.9, 125.5, 121.4, 112.3, 109.4, 49.9, 49.1, 49.0, 47.2, 44.7, 42.7, 38.1, 35.4, 33.0, 29.2, 10.4 (x2), -1.8, -1.9. **HRMS (ESI) m/z:** [M+Na]$^+$ Calcd. for $C_{26}H_{46}N_2O_4S_2Si_2Na$ 593.2330; Found 593.2340.

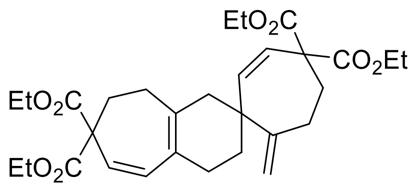

2g

Compound **2g** was obtained from bisallene **1g** (23.8 mg, 0.09 mmol) following the general procedure **GP1**. Purification by column chromatography (silica gel, 40–63 µm, Hexanes/EtOAc 90:10 to 60:40 v/v) provided **2g** (19.0 mg, 80% yield) as a colourless oil.

MW ($C_{30}H_{40}O_8$): 528.64 g/mol; **Rf**: 0.44 (Hexane/EtOAc 8:2); **IR (ATR) *ν* (cm^{-1})**: 2929, 1725, 1224. **^{1}H NMR (CDCl$_3$, 400 MHz):** δ 5.85 (d, 1H, $^3J_{cis}$ = 12.4 Hz), 5.80 (d, 1H, $^3J_{cis}$ = 12.4 Hz), 5.70 (d, 1H, $^3J_{cis}$ = 12.1 Hz), 5.57 (d, 1H, $^3J_{cis}$ = 12.1 Hz), 4.81 (s, 1H), 4.69 (s, 1H), 4.23 – 4.11 (m, 8H), 2.62 – 2.44 (m, 2H), 2.44 – 2.25 (m, 5H), 2.25 – 1.98 (m, 5H), 1.77 – 1.59 (m, 2H), 1.30 – 1.20 (m, 12H). **^{13}C{^{1}H} NMR (CDCl$_3$, 101 MHz):** δ 171.5, 171.2, 171.1, 171.0, 149.9, 139.8, 138.0, 132.8, 125.8, 125.7, 125.5, 112.5, 61.8 (x2), 61.7 (x2), 61.5, 58.6, 43.9, 43.8, 33.5, 32.7, 32.0, 31.7, 30.8, 28.8, 14.2 (x2), 14.1 (x2). **HRMS (ESI) m/z:** [M+Na]$^+$ Calcd. for $C_{30}H_{40}O_8Na$ 551.2615; Found 551.2615.

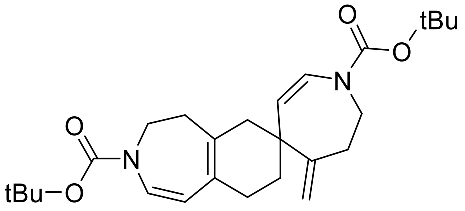

2h

Compound **2h** was obtained from bisallene **1h** (19.7 mg, 0.09 mmol) following the general procedure **GP1**. Purification by column chromatography (silica gel, 40–63 μm, Hexanes/EtOAc 90:10 to 60:40 v/v) provided **2h** (8.3 mg, 42% yield) as a colourless oil.

MW ($C_{26}H_{38}N_2O_4$): 442.60 g/mol; **Rf**: 0.63 (Hexane/EtOAc 8:2); **IR (ATR) ν (cm^{-1})**: 2973, 1697, 1649, 1451, 1431; **^{1}H NMR ($CDCl_3$, 400 MHz):** δ 6.89 – 6.56 (bs,[a] 1H), 6.55 – 6.24 (bs, 1H), 4.86 (s, 1H), 4.84 – 4.71 (bs, 1H), 4.73 (s, 1H), 4.72 – 4.59 (bs, 1H), 3.81 – 3.59 (m, 4H), 2.55 (bs, 2H), 2.42 – 2.29 (m, 3H), 2.25 – 2.05 (m, 3H), 1.85 – 1.71 (bs, 1H), 1.71 – 1.61 (m, 1H), 1.48 (s, 9H), 1.46 (s, 9H). **^{13}C{^{1}H} NMR ($CDCl_3$, 101 MHz):** δ 153.7, 152.5, 150.4, 132.9, 127.6, 126.1, 125.4, 120.5, 111.3, 109.6, 81.3, 80.7, 47.6, 44.7, 44.4, 42.5, 37.7, 34.7, 33.0, 29.5, 28.4 (x2). **HRMS (ESI) m/z:** $[M+Na]^+$ Calcd. for $C_{26}H_{38}N_2O_4Na$ 465.2724; Found 465.2718.

[a] bs stands for broad signal.

Identification of the single regioisomer 2A (product 2a)

Figure S1. All possible chemo- and regioisomers of **2a**

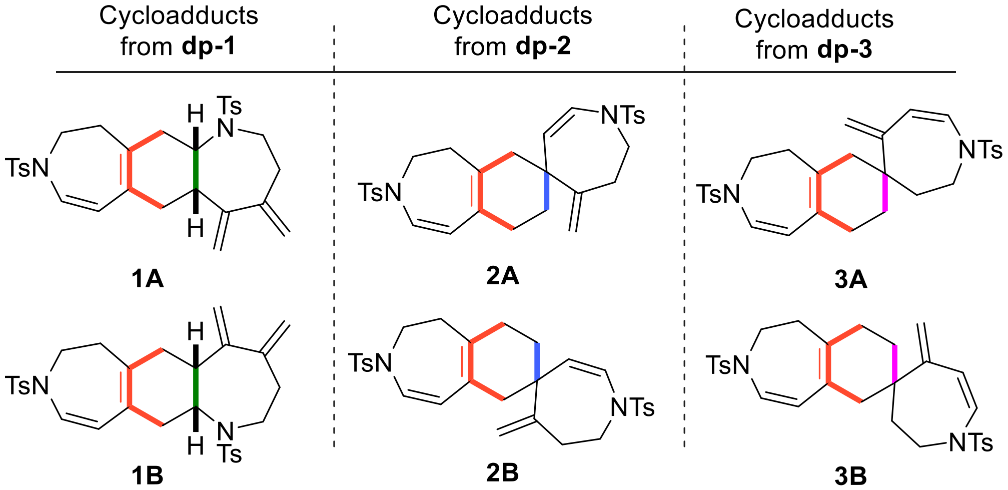

Figure S2. COSY and HMBC crosspeaks, and NOE contacts observed in **2a** confirming the formation of cycloadduct **2A**

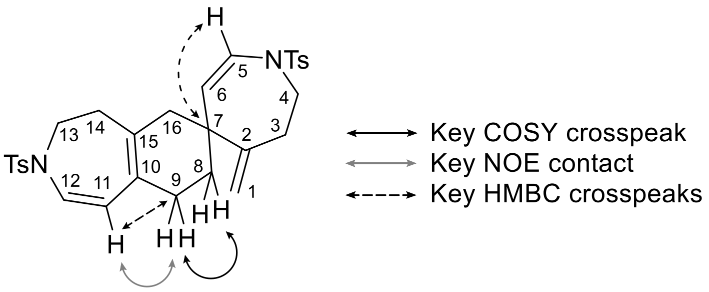

2a (cycloadduct **2A**)

The shifts of the six olefinic protons and their multiplicity in the 1H-NMR spectrum were first analyzed. Two pair of doublets, at δ = 4.71 and 6.27 ppm, and δ = 4.84 and 6.64 ppm, are characteristic of a structure that has two *cis* endocyclic double bonds. In addition, only two singlets at δ = 4.52 and 4.63 ppm are observed, corresponding to the two geminal protons of a single exocyclic double bond. This allowed us to discard cycloadducts **1A** and **1B**. To distinguish between cycloadducts formed by reaction of **dp-2** and **dp-3**, 2D NMR experiments were conducted. The HMBC spectrum displays a three-bond correlation between the spiro carbon atom and the proton closest to the nitrogen of one of the *cis* endocyclic olefin (**C7**-**H5**). This is, in fact, a clear demonstration that it is the exocyclic double bond conjugated to the endocyclic double bond that is involved in the Diels-Alder reaction (**dp-2**) and thus cycloadducts **3A** and **3B** can be discarded. Finally, the analysis of the HMBC, COSY and NOESY experiments allowed us to distinguish between regioisomers **2A** and **2B**. NOE contacts between **H9** (**H8** and **H9** identified as the two contiguous methylenic groups by COSY) and **H11** protons led us to assign **2A** as the single cycloadduct formed. The HMBC crosspeak between **H11** and **C9** also supports the formation of the cycloadduct **2A.**

Figure S3. HSQC (blue/red) + HMBC (green) combined spectra

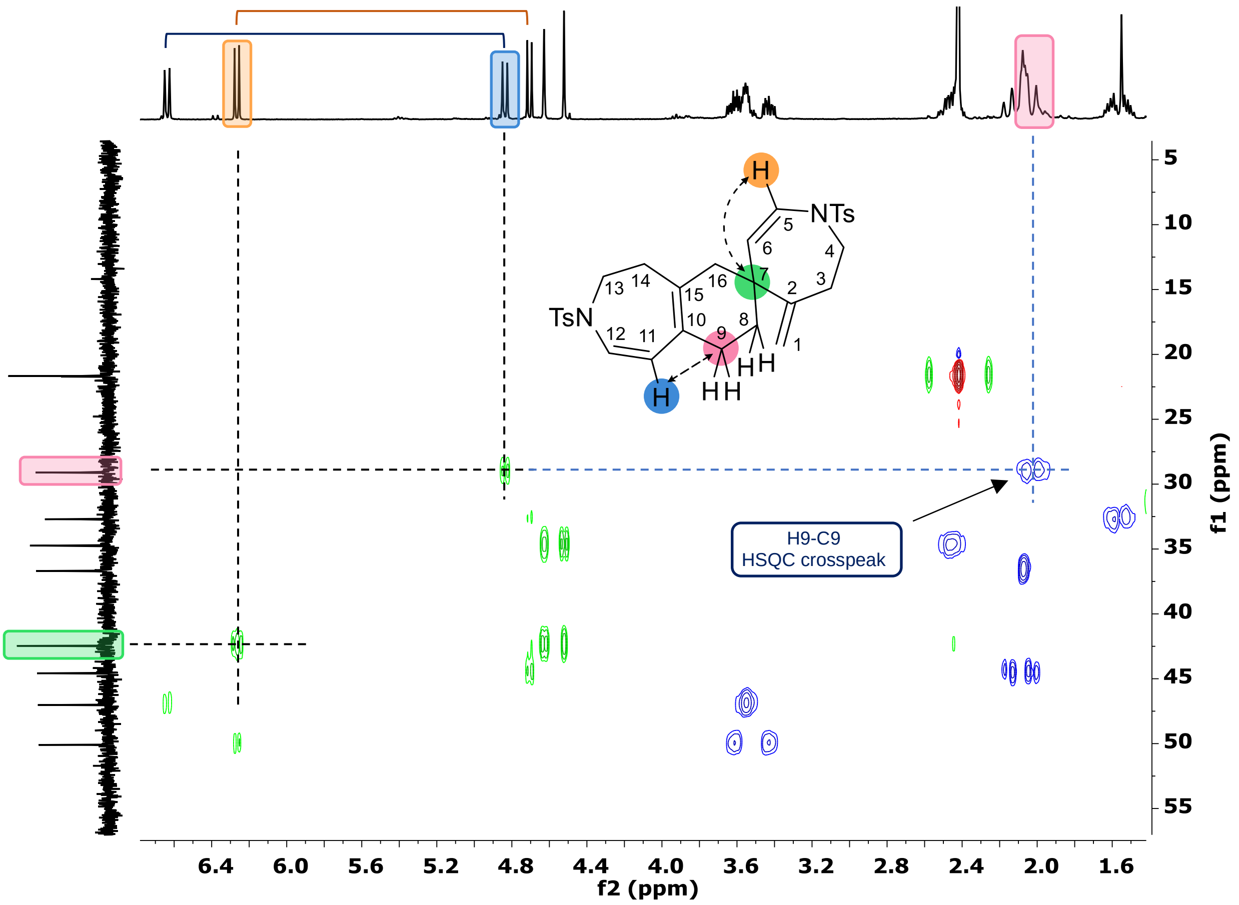

Figure S4. COSY (red) + NOESY (blue) combined spectra

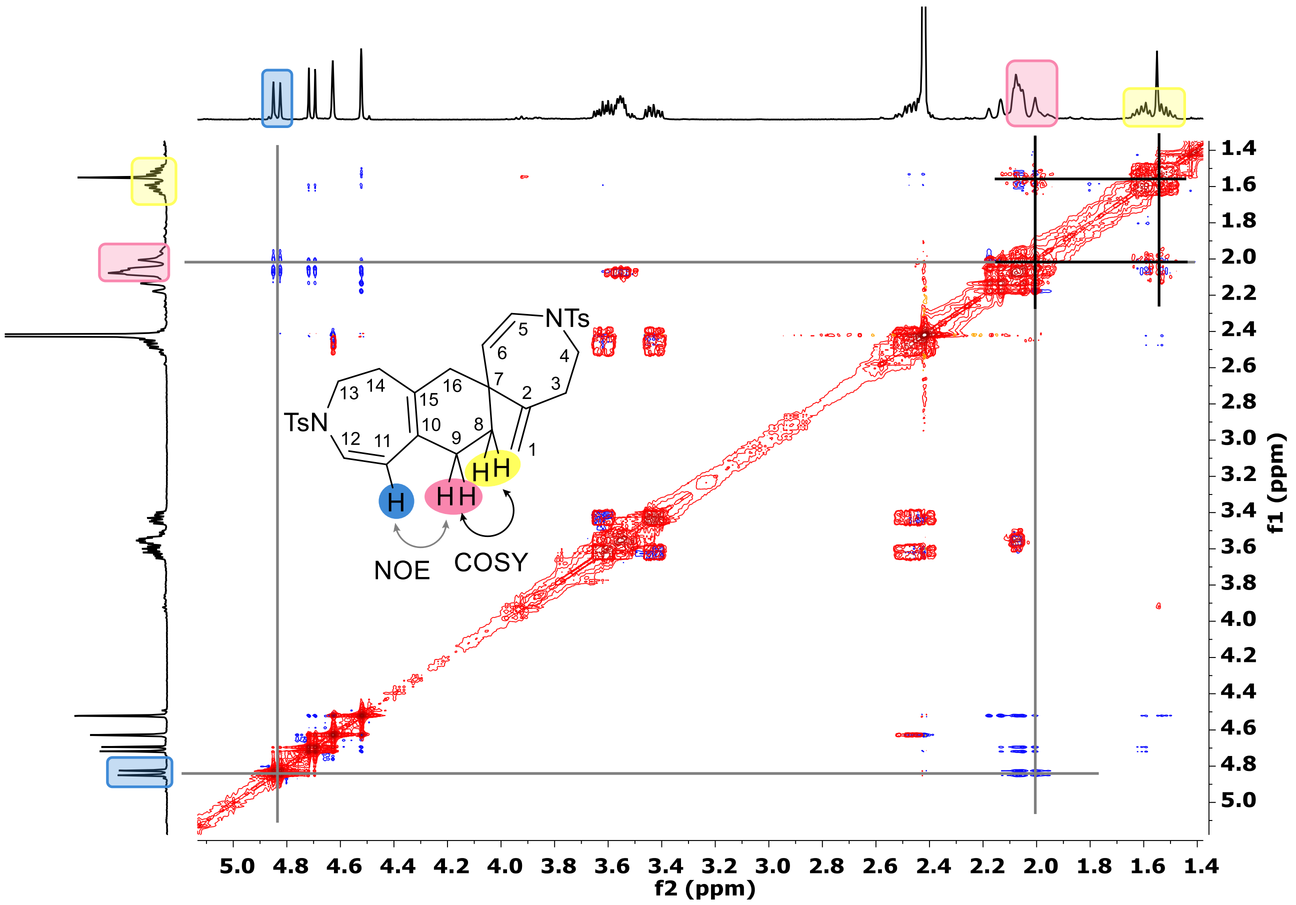

Detection of the biradical intermediate using ESI(+)-MS

Scheme S3. Paths leading to the detected species containing **TEMPO**

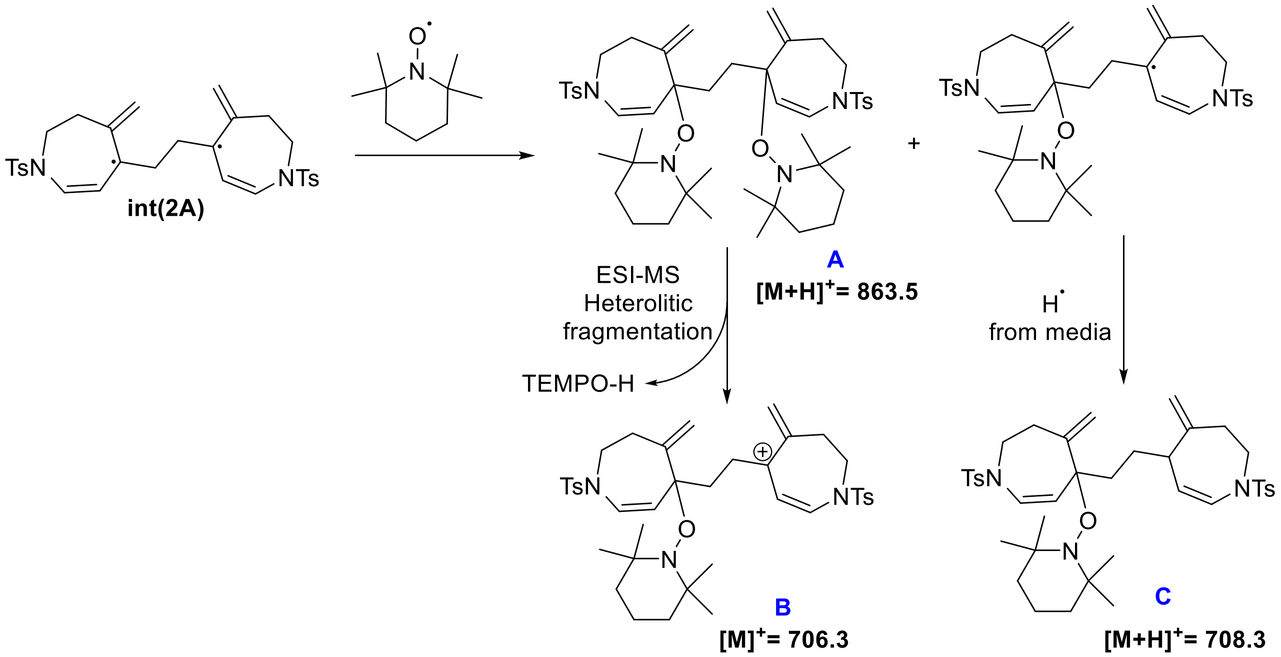

Figure S5. ESI-MS detection of **[int(2A)+2TEMPO+H]**+ (**A**)

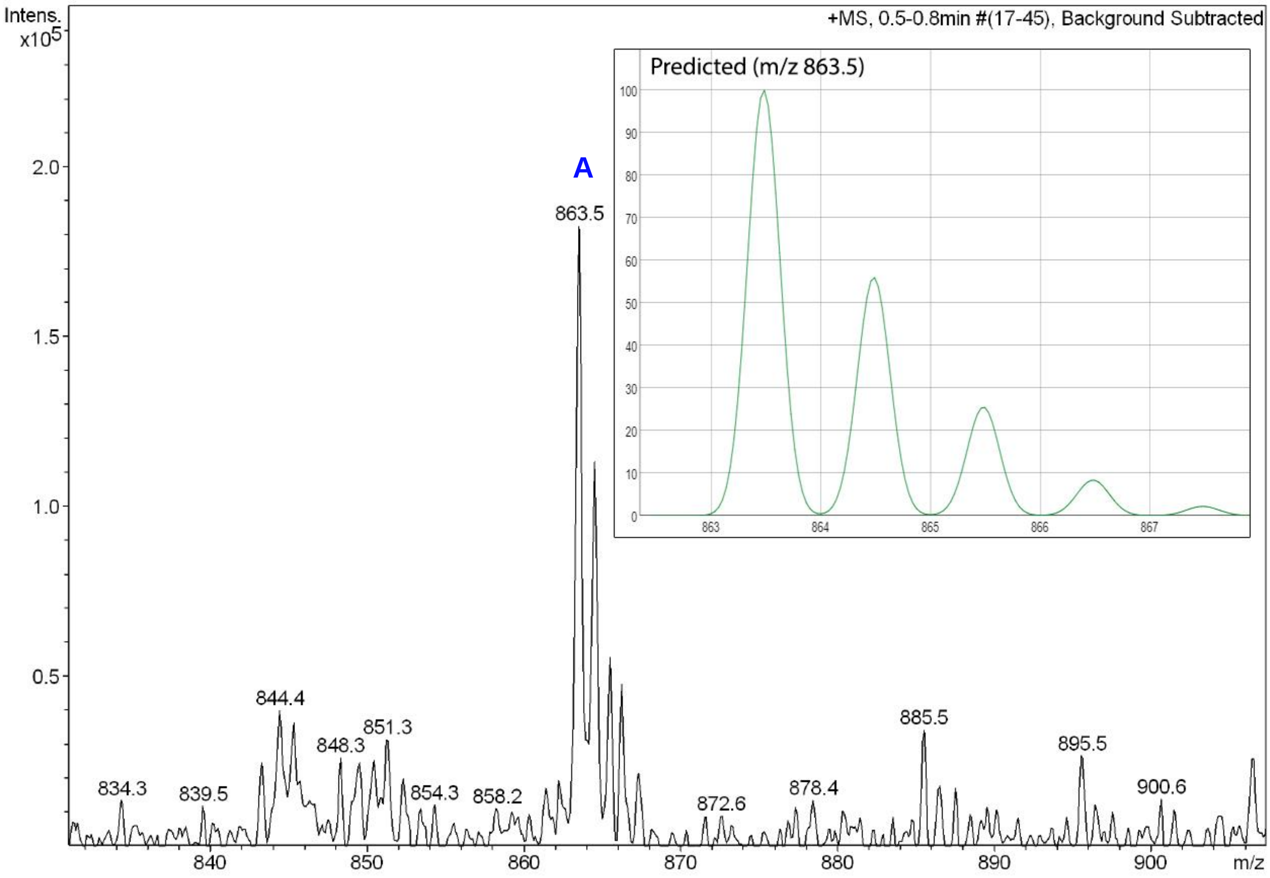

Figure S6. ESI-MS detection of **[int(2A)+TEMPO]$^+$** (**B**) and **[int(2A)H+TEMPO+H]$^+$** (**C**)

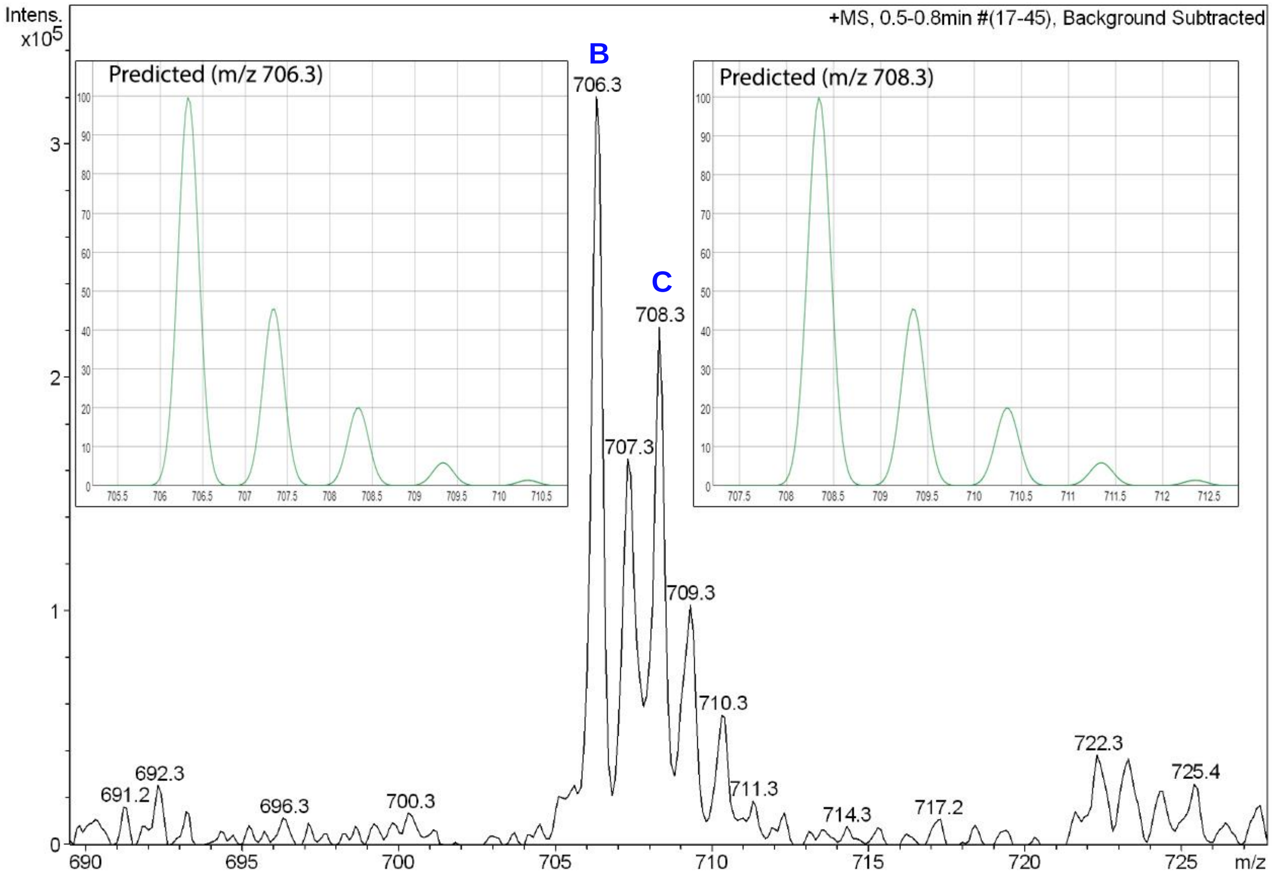

To confirm that **B** is generated from **A** during the ionization process, an MS/MS analysis was conducted. The peak at m/z = 863.5 was fragmented by MS/MS resulting in a peak at m/z = 706.3 corresponding to **B**.

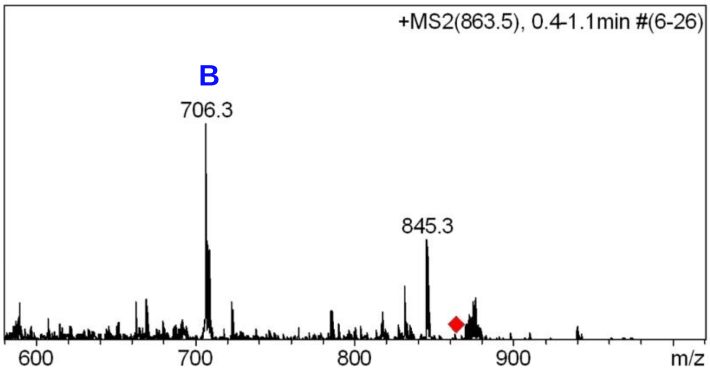

Computational methods

Geometries of all stationary points were optimized without symmetry constraint with the Gaussian 16 program[3] using the DFT B3LYP hybrid exchange-correlation functional[4-6] employing the all-electron cc-pVDZ basis set,[7] **TS^{os}(2A$_{exo}$)** and **TS^{os}(2A$_{endo}$)** were optimized again using UB3LYP/cc-pVDZ. The electronic energies were improved (singlet and triplet states) by performing single point energy calculations with the cc-pVTZ basis set and the (U)B3LYP hybrid exchange-correlation functional including solvent effects corrections computed with the solvent model based on density (SMD) continuum solvation,[8] To mimic the experimental solvent mixture with a molar fraction ratio of 76:24 of THF:CH_2Cl_2, the values of the solvent descriptors used in the SMD solvation model were re-defined on the basis of a linear behaviour with the molar fraction. Using the "Solvent=(Generic,Read)" options of the Gaussian16 SCRF keyword, the solvent mixture was defined employing the following solvent descriptors: Dynamic Dielectric Constant=1.410; Static Dielectric Constant=1.4085; Abraham's hydrogen bond acidity=0.02424; Abraham's hydrogen bond basicity=0.3758; Surface Tension=39.3697; Carbon Aromaticity=0; Electronegativity Halogenicity=0.1617. The D3 Grimme energy corrections for dispersion[9] with the original damping function were added in all (U)B3LYP/cc-pVDZ and (U)B3LYP/cc-pVTZ calculations. Analytical Hessians were computed to determine the nature of stationary points (one and zero imaginary frequencies for TSs and minima, respectively) and to calculate unscaled zero-point energies (ZPEs) as well as thermal corrections and entropy effects using the standard statistical-mechanics relationships for an ideal gas.[9] These two latter terms were computed at 313.15 K and 1 atm to provide the reported relative Gibbs energies. As a summary, the reported Gibbs energies contain electronic energies including solvent effects calculated at the (U)B3LYP-D3/cc-pVTZ//(U)B3LYP-D3/cc-pVDZ level together with gas phase thermal and entropic contributions computed at 313.15 K and 1 atm with the (U)B3LYP-D3/cc-pVDZ method. All stationary points were unambiguously confirmed by IRC calculations. For the Condensed Fukui functions, the natural charges were obtained performing the natural population analysis of the neutral, cationic and anionic **cHT** at (U)B3LYP-D3/cc-pVTZ//(U)B3LYP-D3/cc-pVDZ theory level.

Figure S7. Transition state Gibbs energy barrier of all reaction paths computed at 313.15K and 1 atm with the (U)B3LYP-D3/cc-pVTZ/SMD(76% THF, 24% CH_2Cl_2)//(U)B3LYP-D3/cc-pVDZ.

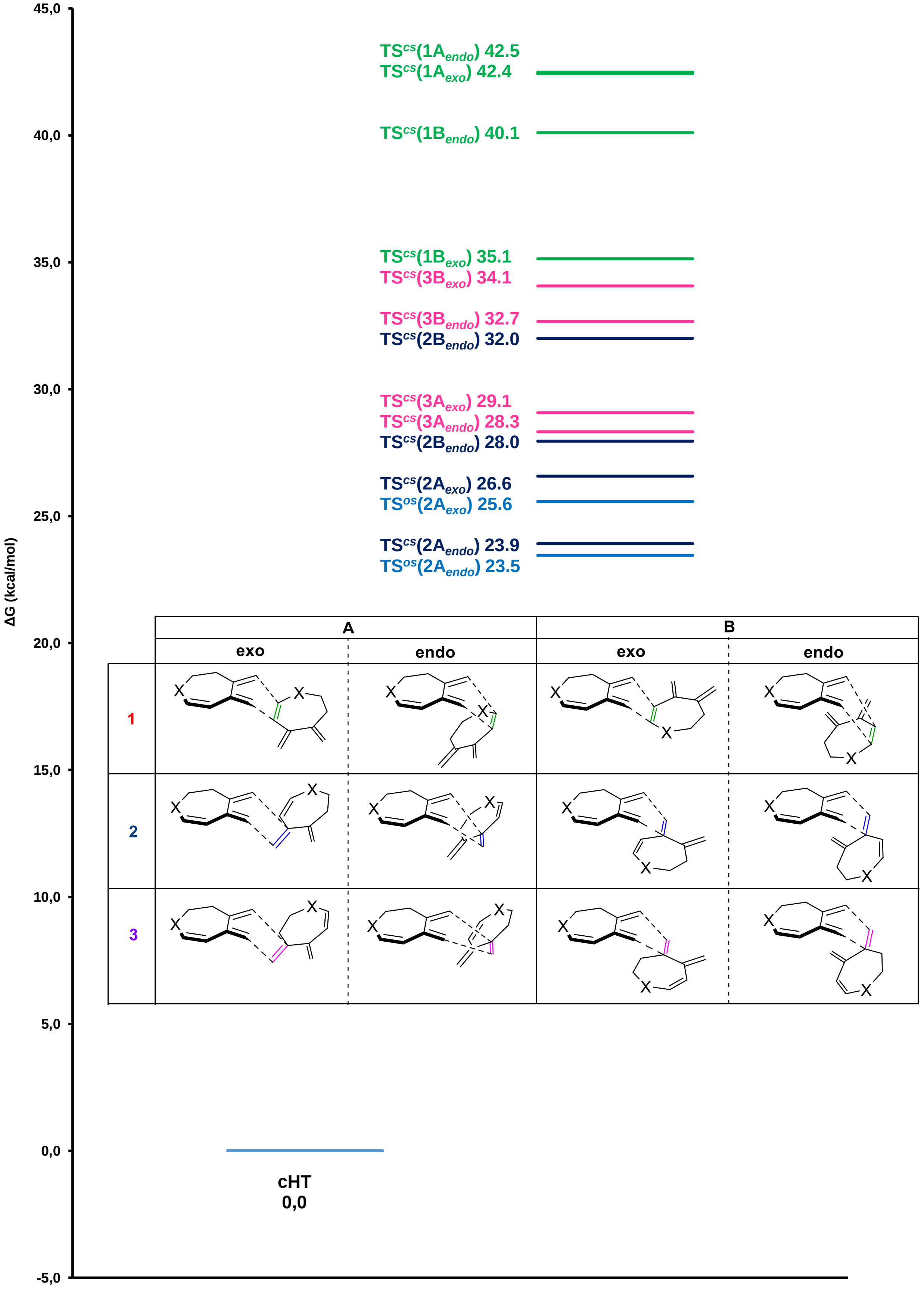

Figure S8. Molecular structures of (a) **TS^{cs}(1A$_{exo}$)**, (b) **TS^{cs}(1A$_{endo}$)**, (c) **TS^{cs}(1B$_{exo}$)**, (d) **TS^{cs}(1B$_{endo}$)**, (e) **TS^{cs}(2A$_{exo}$)**, (f) **TS^{cs}(2A$_{endo}$)**, (g) **TS^{cs}(2B$_{exo}$)**, (h) **TS^{cs}(2B$_{endo}$)**, (i) **TS^{cs}(3A$_{exo}$)**, (j) **TS^{cs}(3A$_{endo}$)**, (k) **TS^{cs}(3B$_{exo}$)**, (l) **TS^{cs}(3B$_{endo}$)**, (m) **TS^{os}(2A$_{exo}$)**, (n) **TS^{os}(2A$_{endo}$)**, (o) **TS^{os}(int(2A$_{exo}$)**, (p) **TS^{os}(int(2A$_{endo}$))**. Distances are given in Angstroms (Å).

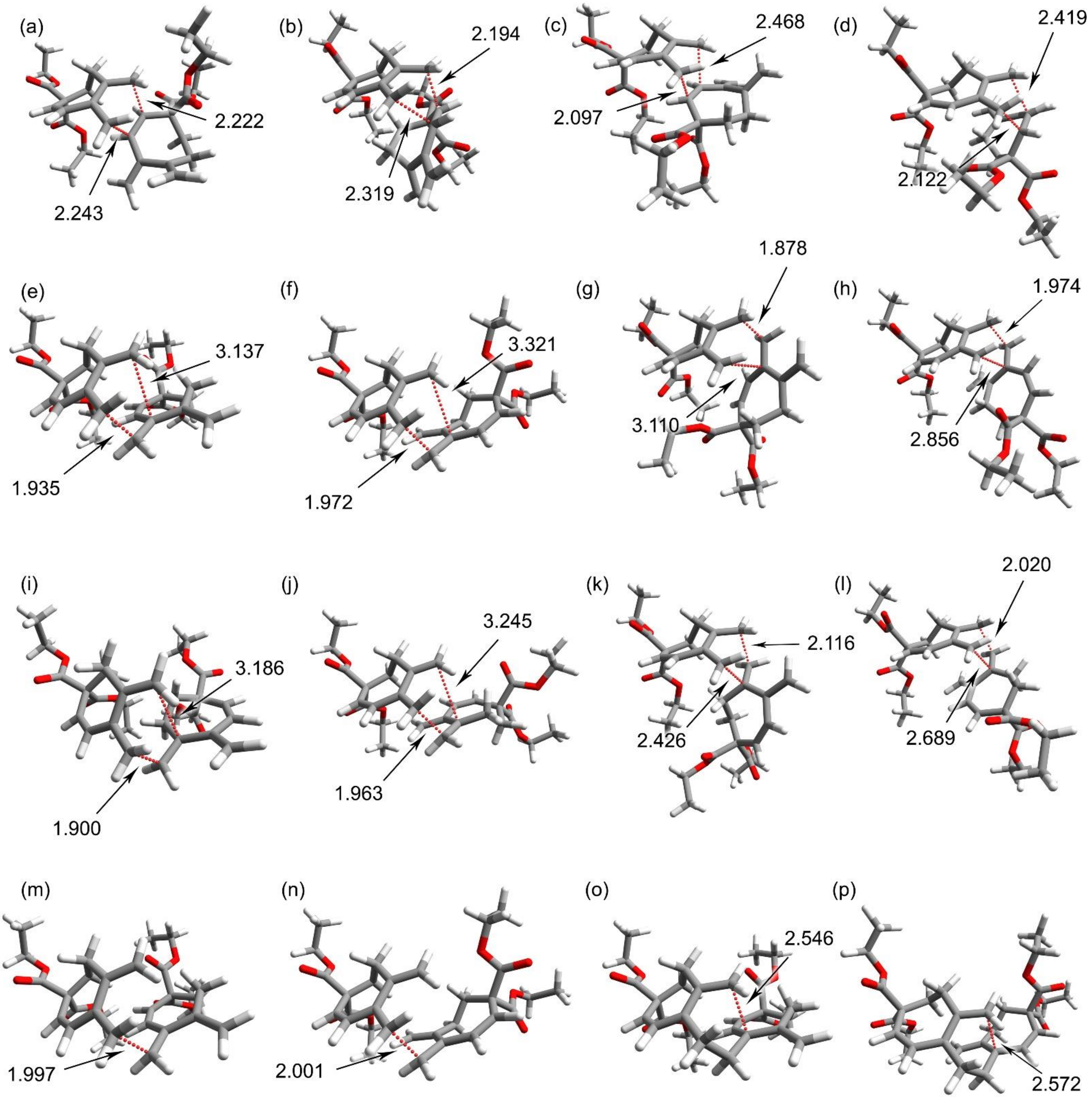

Table S1. Closed-shell singlet, Open-shell singlet and Open-shell triplet energy comparisson of all reaction pathways computed at 313.15K and 1 atm with the (U)B3LYP-D3/cc-pVTZ/SMD(76% THF, 24% CH_2Cl_2)//(U)B3LYP-D3/cc-pVDZ.

	**Closed-shell E (Hartrees)**	**Open-shell singlet (oss) E (Hartrees)**	**S^2 (oss)**	**Open-shell triplet (ost) E (Hartrees)**	**S^2 (ost)**	**ΔE_{oss-cs} (kcal/mol)**	**ΔE_{ost-cs} (kcal/mol)**
**TS(1A$_{exo}$)**	-1769,71922097	-1769,71922097	0,000	-1769,60318767	2,070	0,00	72,8
**TS(1A$_{endo}$)**	-1769,72084150	-1769,72084150	0,000	-1769,62617458	2,034	0,00	59,4
**TS(1B$_{exo}$)**	-1769,73199710	-1769,73199710	0,000	-1769,66383335	2,046	0,00	42,8
**TS(1B$_{endo}$)**	-1769,72526752	-1769,72526752	0,000	-1769,65122395	2,049	0,00	46,5
**TS(2A$_{exo}$)**	-1769,74405463	-1769,74446238	0,187	-1769,72033707	2,055	-0,26	14,9
**TS(2A$_{endo}$)**	-1769,74691542	-1769,74703649	0,046	-1769,71978683	2,054	-0,08	17,0
**TS(2B$_{exo}$)**	-1769,73657120	-1769,73789462	0,399	-1769,71849134	2,058	-0,83	11,3
**TS(2B$_{endo}$)**	-1769,73915198	-1769,73915198	0,000	-1769,69684649	2,061	0,00	26,5
**TS(3A$_{exo}$)**	-1769,74014645	-1769,74078180	0,286	-1769,71718532	2,055	-0,40	14,4
**TS(3A$_{endo}$)**	-1769,73851304	-1769,73851304	0,000	-1769,15419778	2,060	0,00	366,7
**TS(3B$_{exo}$)**	-1769,73339295	-1769,73339295	0,000	-1769,65003738	2,043	0,00	52,3
**TS(3B$_{endo}$)**	-1769,73081215	-1769,73081215	0,000	-1769,66846766	2,062	0,00	39,1

Open-shell singlet and Open-shell triplet of all reaction paths were also evaluated to confirm that **TS(2A$_{exo}$)** and **TS(2A$_{endo}$)** are the lowest energy barrier transition states.

Figure S9. Frontier orbitals HOMO for unpaired electrons *α* and *β* of (a) **int(2A$_{exo}$)**, and (b) **int(2A$_{endo}$)**. Rendered at 0.026 isosurface contour values.

(a)

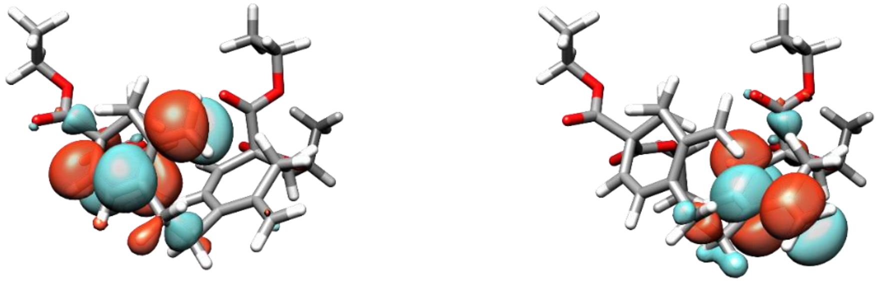

α *β*

(b)

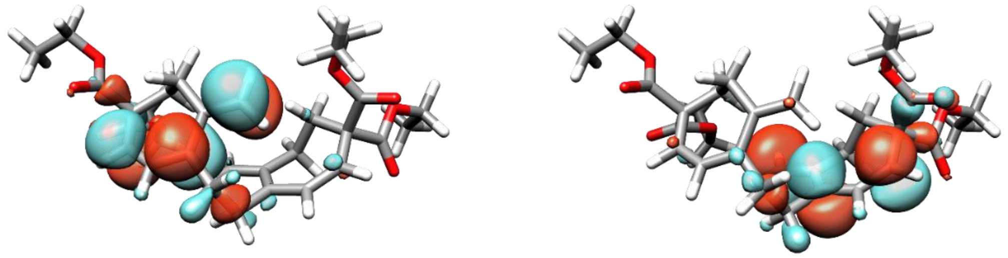

α *β*

Computational data

All DFT data obtained within this study are provided through the following link:

http://dx.doi.org/10.19061/iochem-bd-4-37

^{1}H and ^{13}C NMR spectra

Bisallene 1h

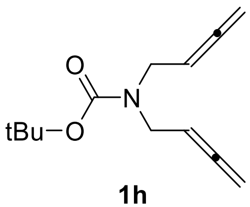

1h

^{1}H NMR (400 MHz, $CDCl_3$)

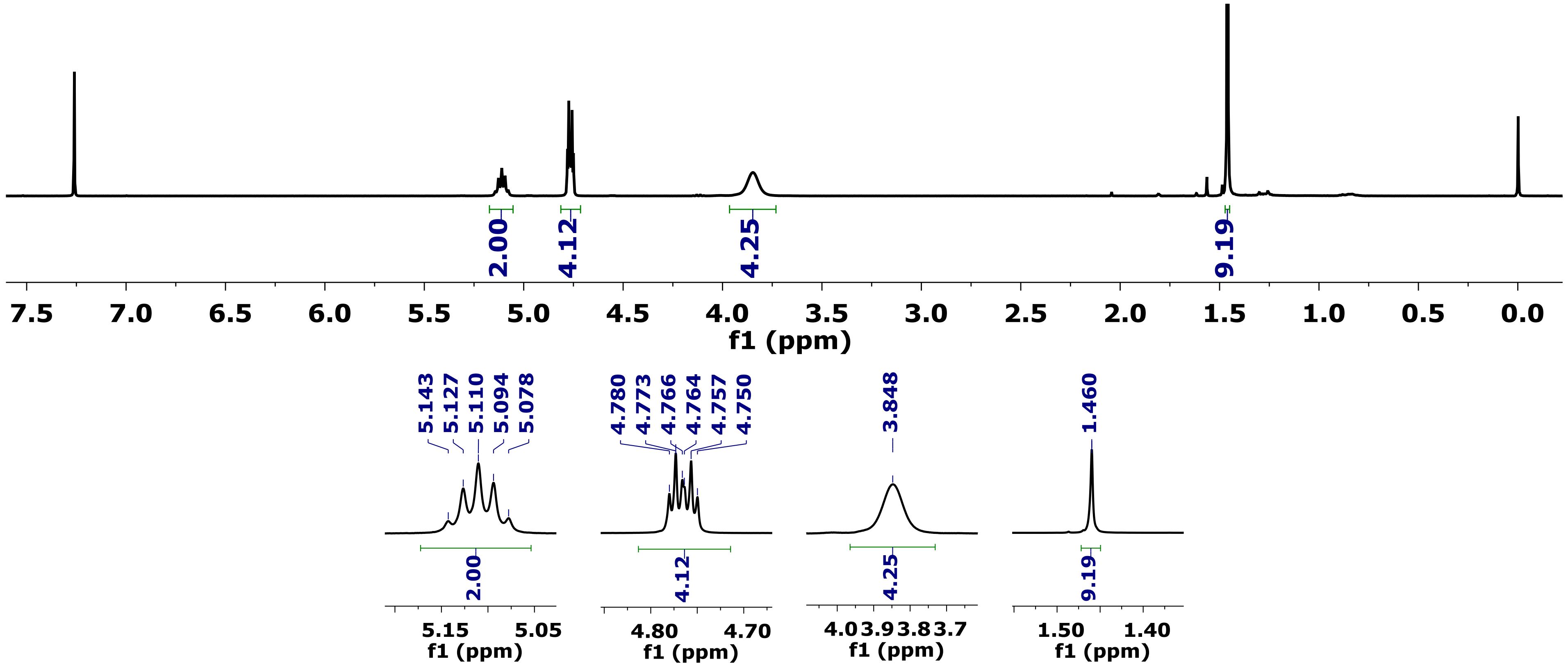

$^{13}C\{^{1}H\}$ NMR (101 MHz, $CDCl_3$)

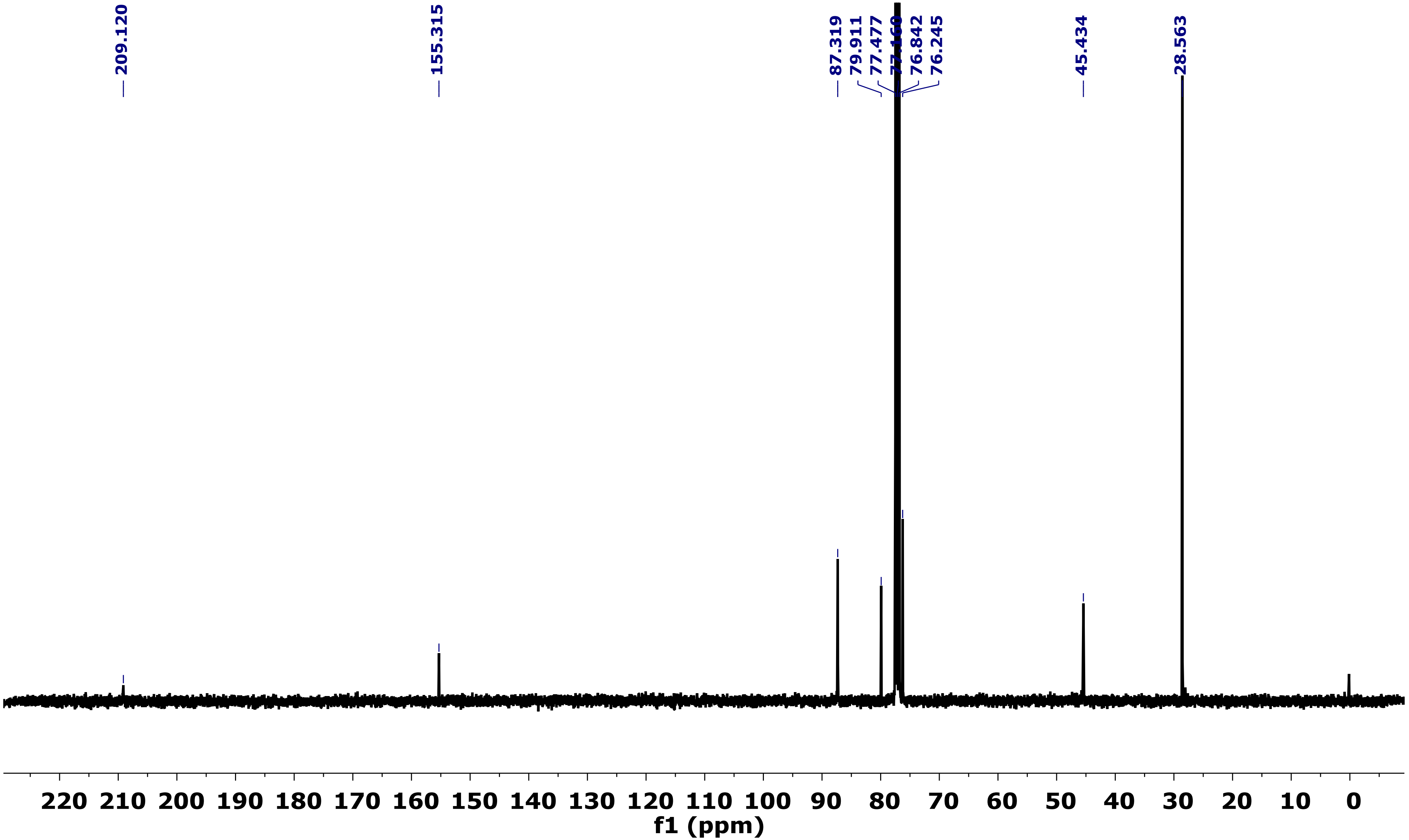

Product 2a

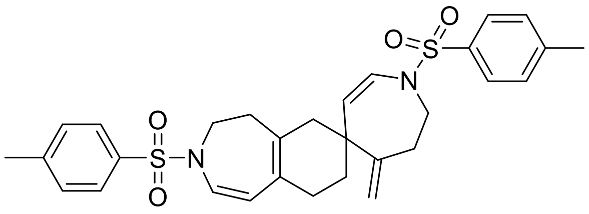

^{1}H NMR (400 MHz, $CDCl_3$)

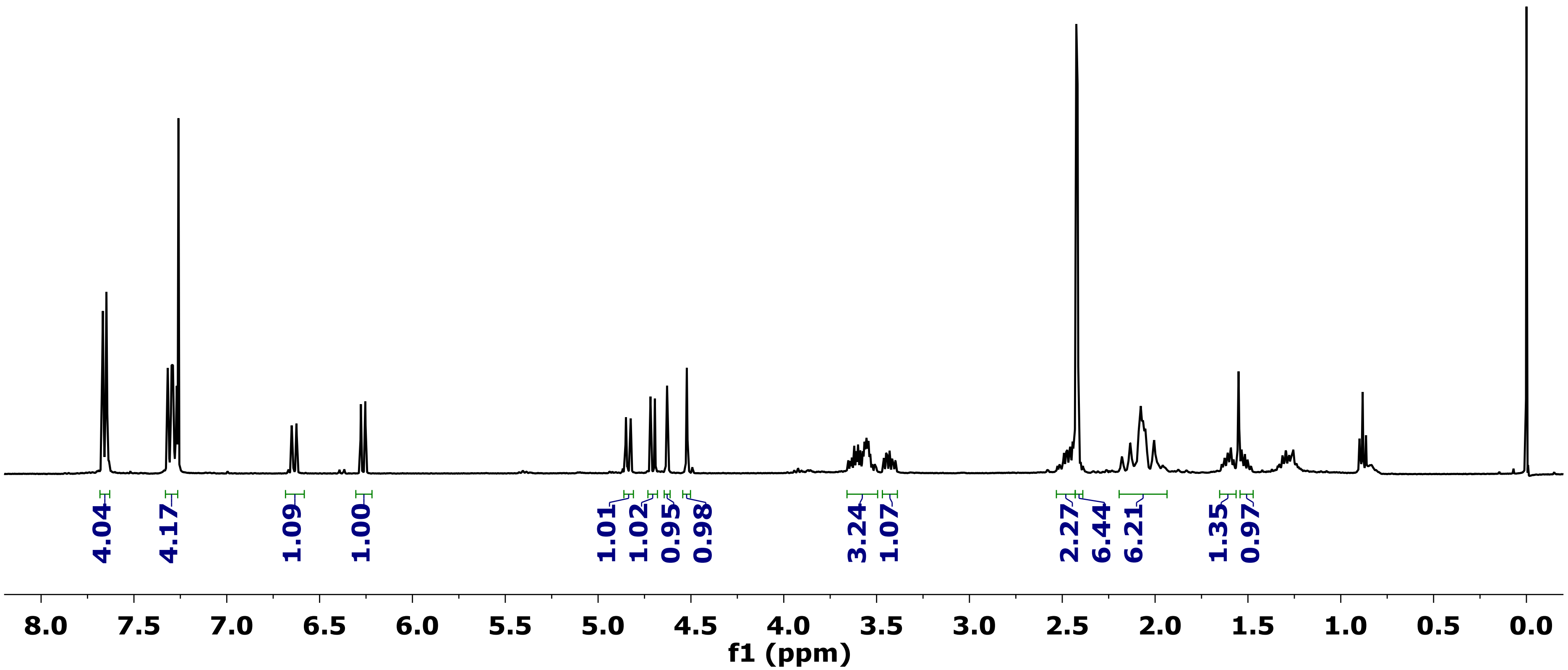

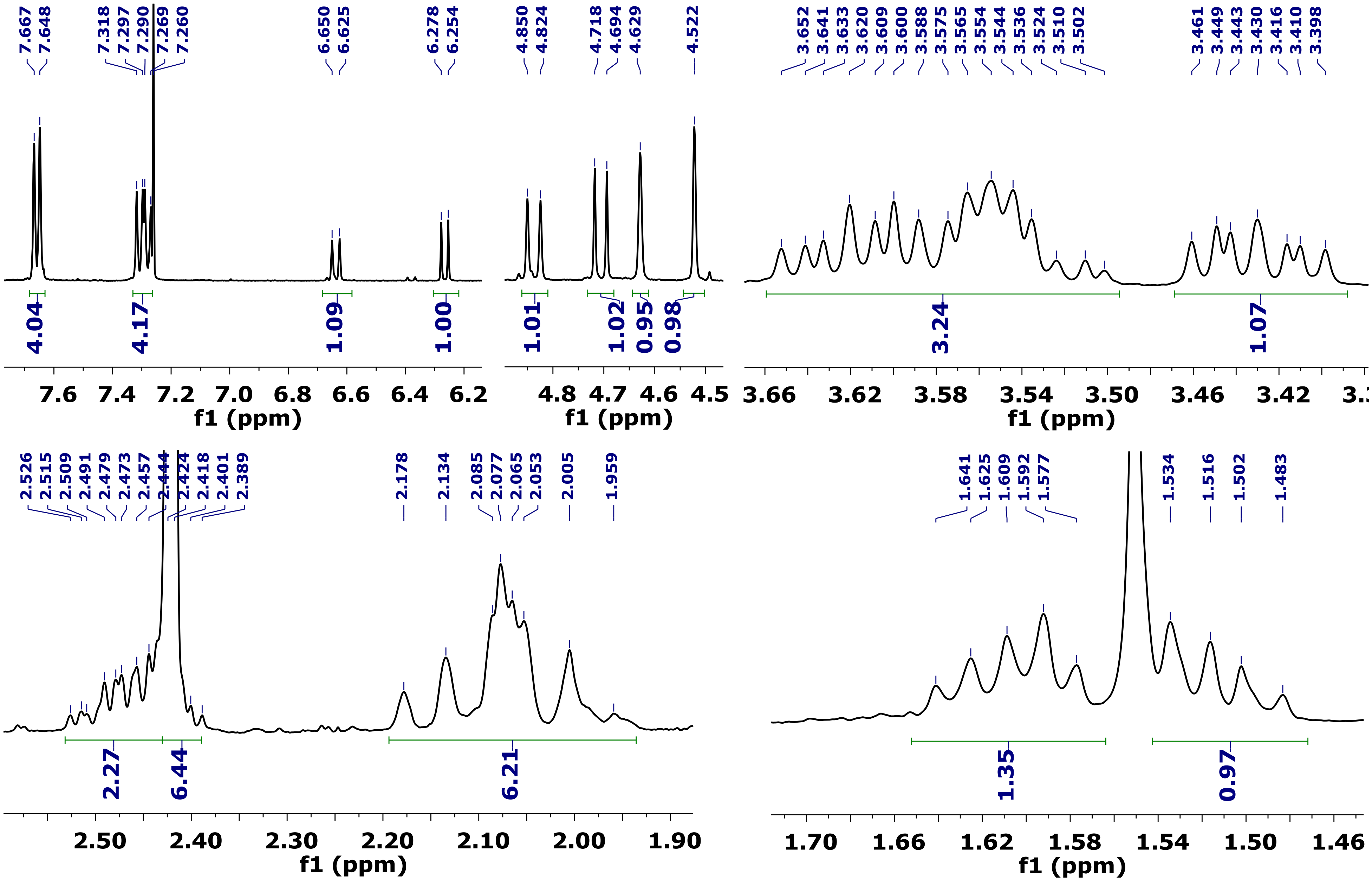

7.667
7.648
7.318
7.297
7.290
7.269
7.260
6.650
6.625
6.278
6.254
4.850
4.824
4.718
4.694
4.629
4.522
3.652
3.641
3.633
3.620
3.609
3.600
3.588
3.575
3.565
3.554
3.544
3.536
3.524
3.510
3.502
3.461
3.449
3.443
3.430
3.416
3.410
3.398
4.04
4.17
1.09
1.00
1.01
1.02
0.95
0.98
3.24
1.07
7.6 7.4 7.2 7.0 6.8 6.6 6.4 6.2
4.8 4.7 4.6 4.5
3.66 3.62 3.58 3.54 3.50 3.46 3.42
f1 (ppm)
2.526
2.515
2.509
2.491
2.479
2.473
2.457
2.444
2.424
2.418
2.401
2.389
2.178
2.134
2.085
2.077
2.065
2.053
2.005
1.959
1.641
1.625
1.609
1.592
1.577
1.534
1.516
1.502
1.483
2.27
6.44
6.21
1.35
0.97
2.50 2.40 2.30 2.20 2.10 2.00 1.90
1.70 1.66 1.62 1.58 1.54 1.50 1.46
f1 (ppm)

$^{13}C\{^{1}H\}$ NMR (101 MHz, $CDCl_3$)

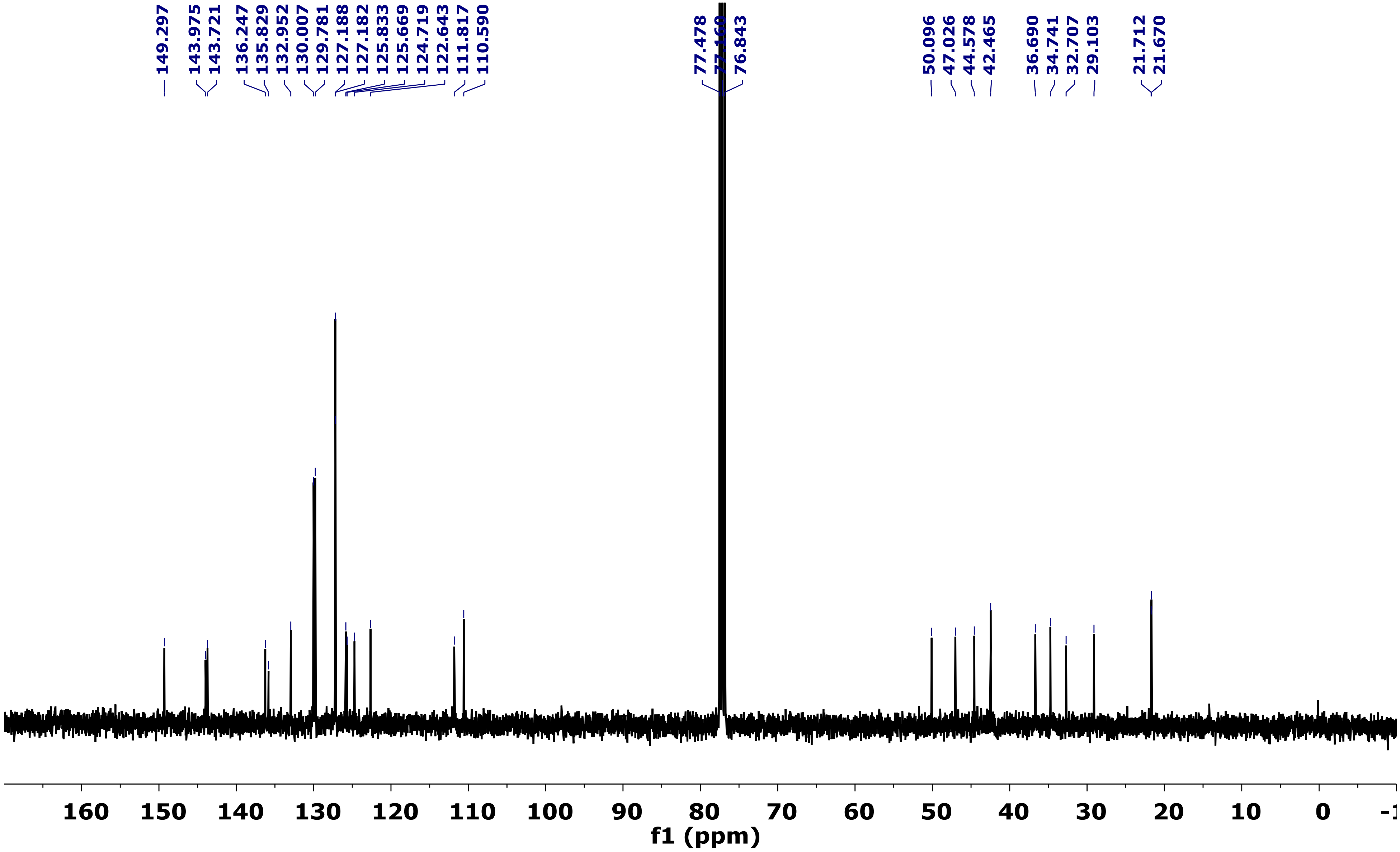

2D NMR HSQC

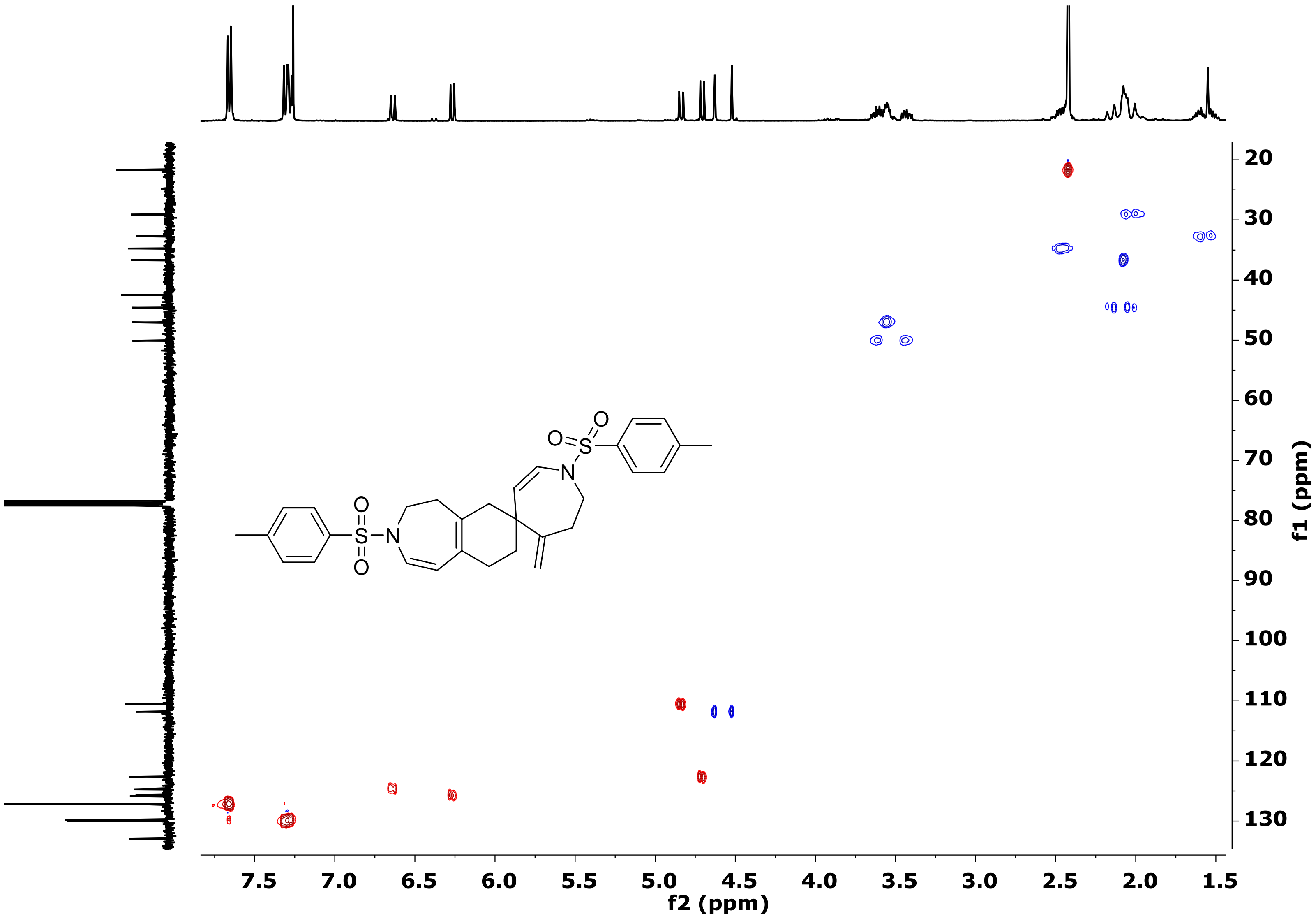

20
30
40
50
60
70
80
90
100
110
120
130
f1 (ppm)
7.5
7.0
6.5
6.0
5.5
5.0
4.5
4.0
3.5
3.0
2.5
2.0
1.5
f2 (ppm)

2D NMR HMBC

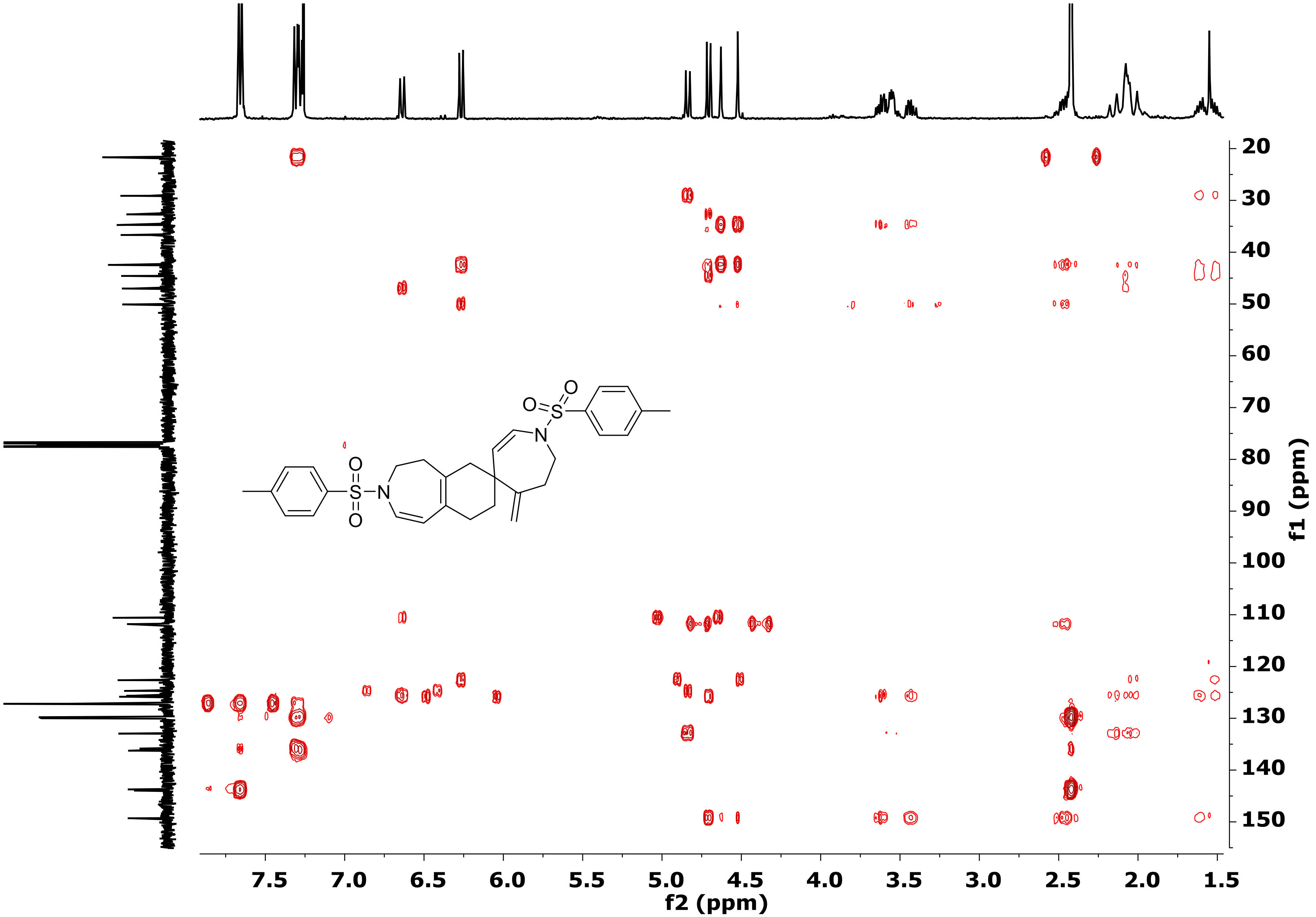

20
30
40
50
60
70
80
90
100
110
120
130
140
150
f1 (ppm)
7.5
7.0
6.5
6.0
5.5
5.0
4.5
4.0
3.5
3.0
2.5
2.0
1.5
f2 (ppm)

2D NMR COSY

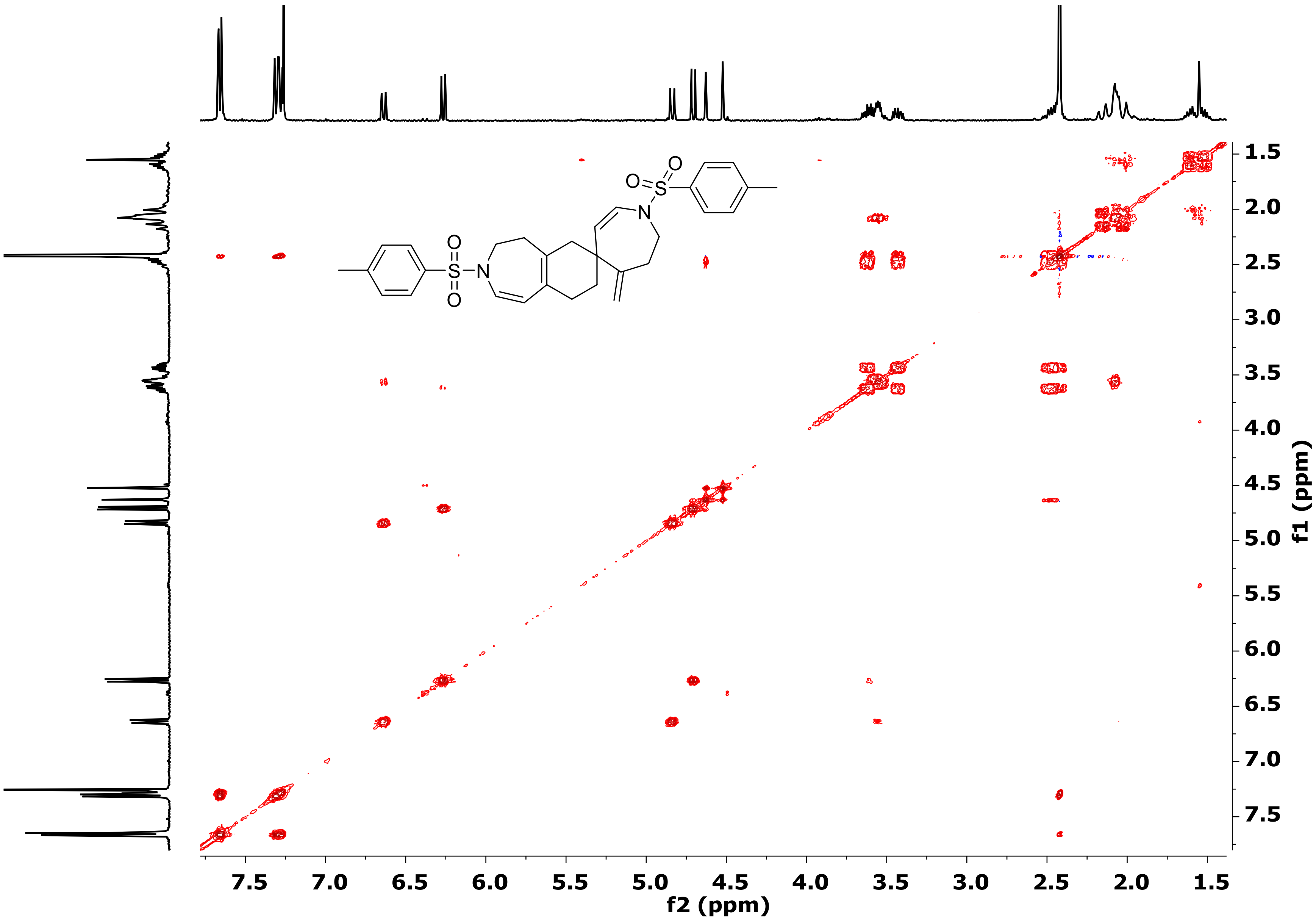

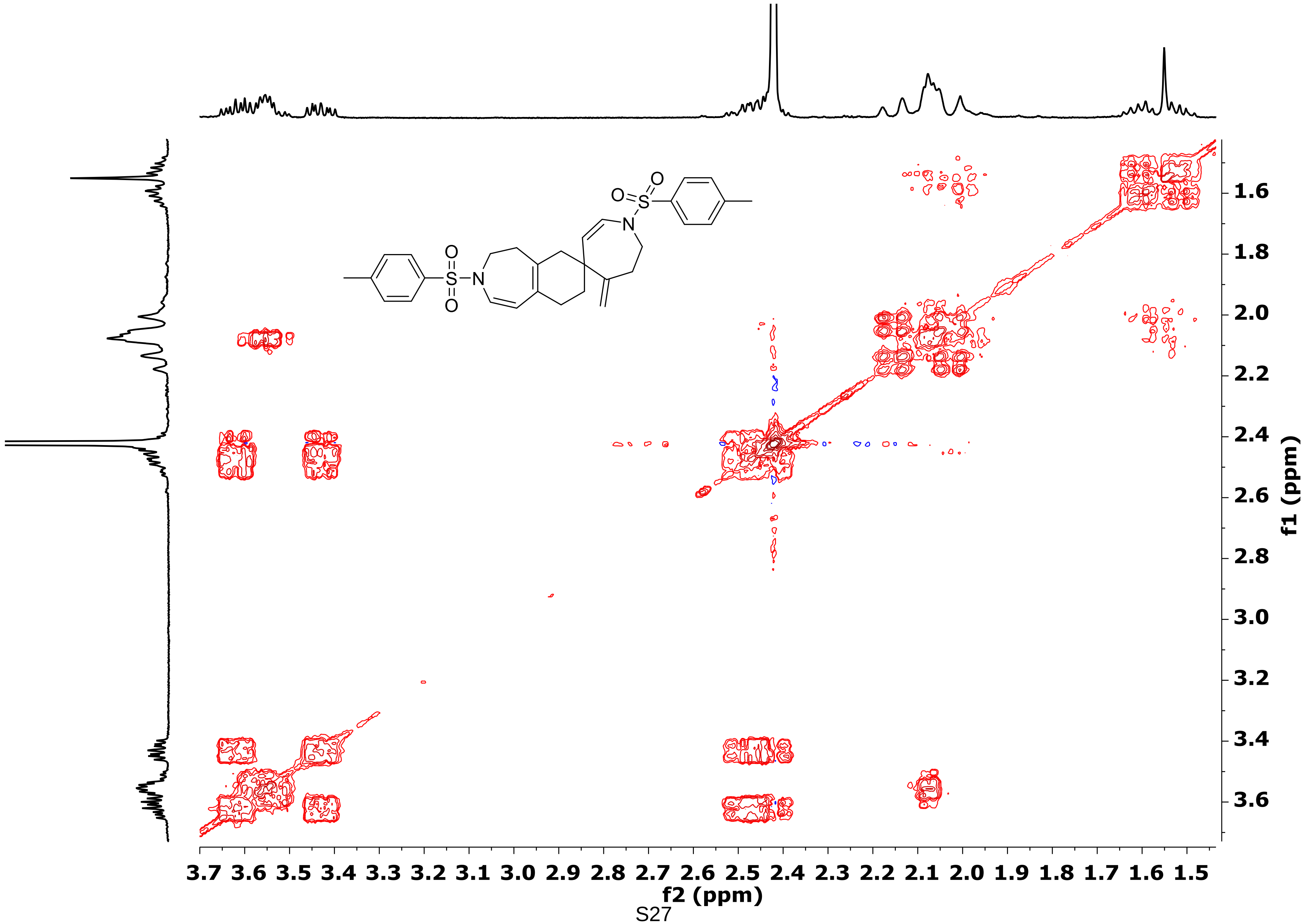
3.7 3.6 3.5 3.4 3.3 3.2 3.1 3.0 2.9 2.8 2.7 2.6 2.5 2.4 2.3 2.2 2.1 2.0 1.9 1.8 1.7 1.6 1.5
f2 (ppm)
1.6 1.8 2.0 2.2 2.4 2.6 2.8 3.0 3.2 3.4 3.6
f1 (ppm)

2D NMR NOESY

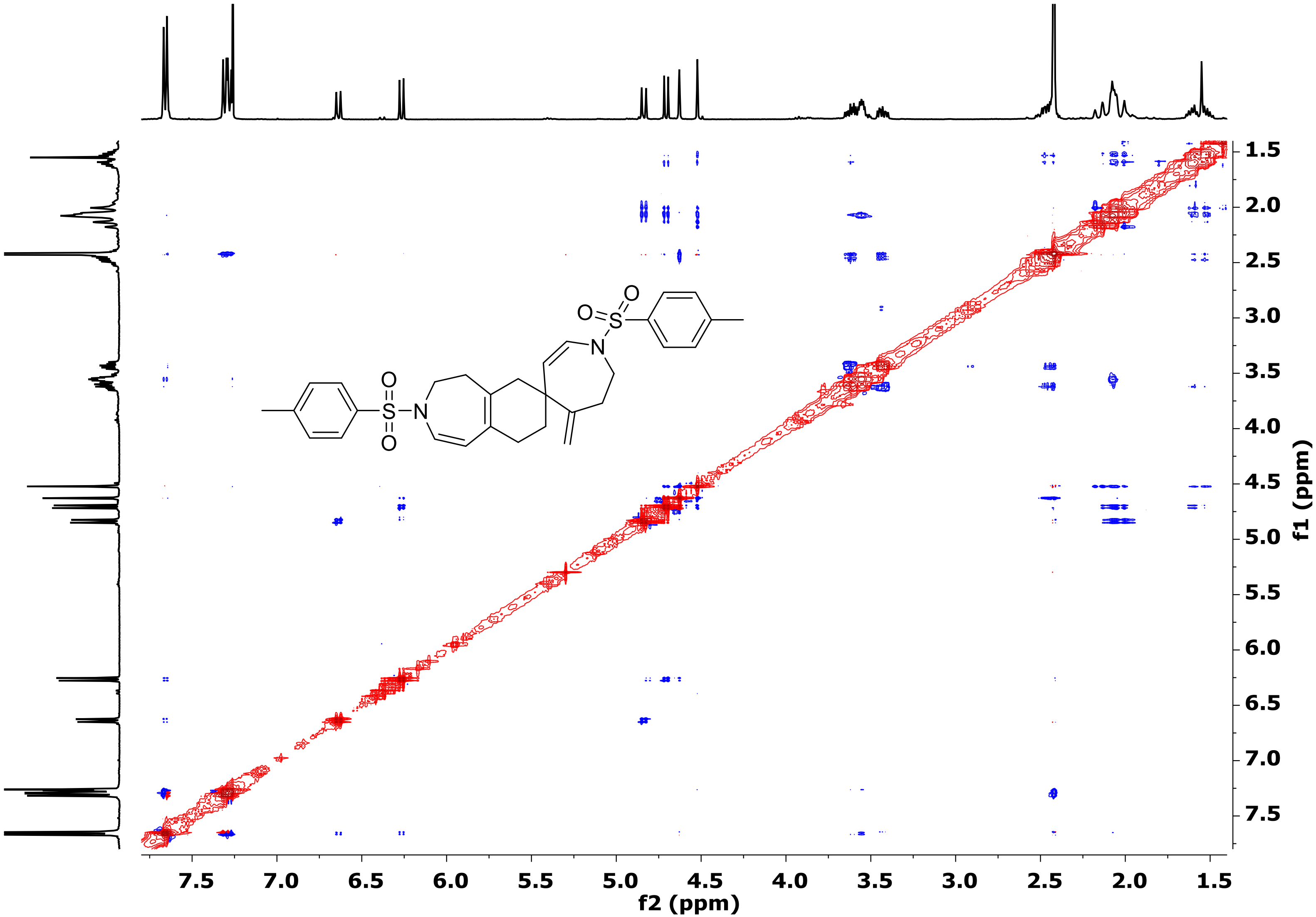

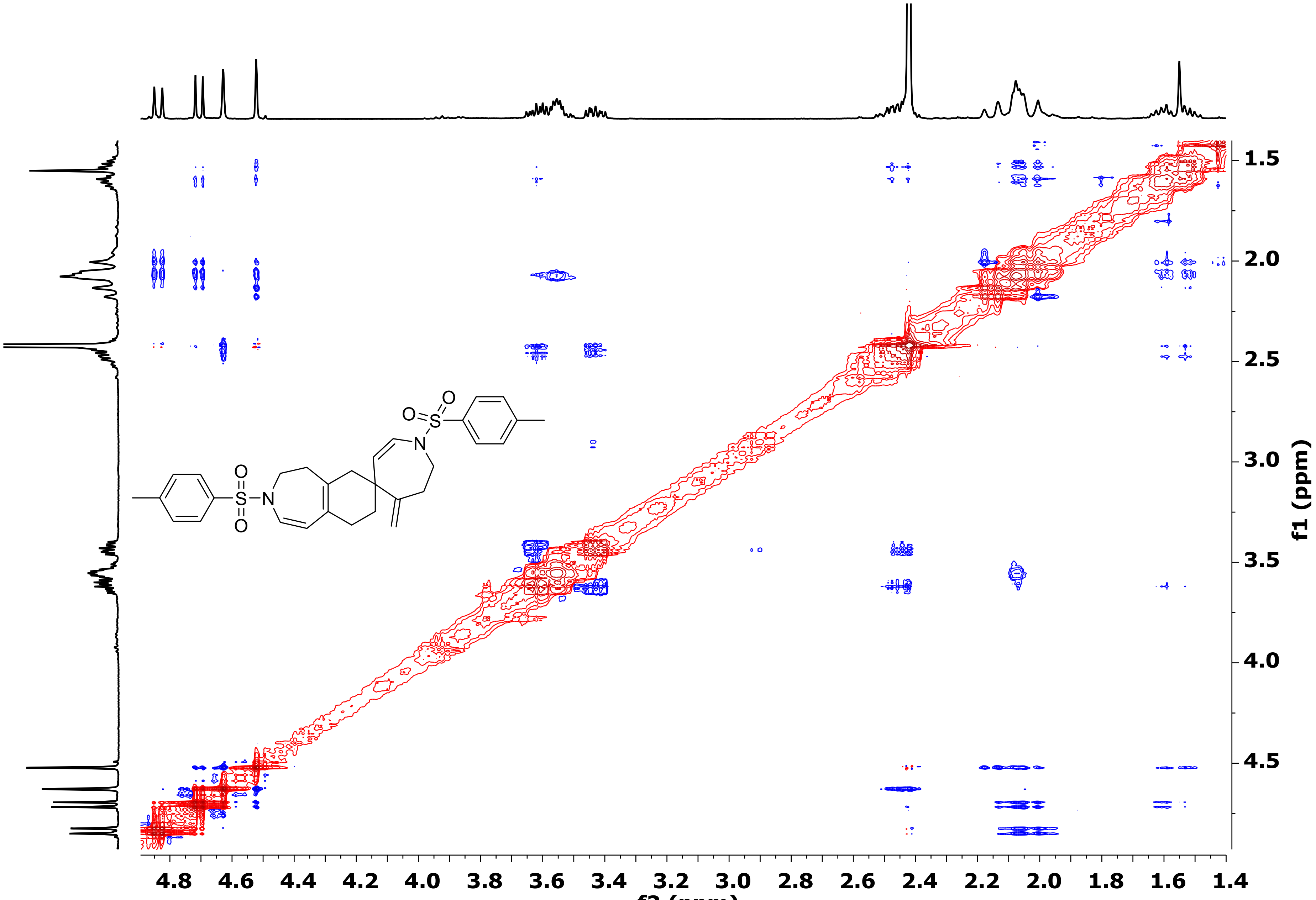

4.8
4.6
4.4
4.2
4.0
3.8
3.6
3.4
3.2
3.0
2.8
2.6
2.4
2.2
2.0
1.8
1.6
1.4
f2 (ppm)
1.5
2.0
2.5
3.0
3.5
4.0
4.5
f1 (ppm)

Product 2b

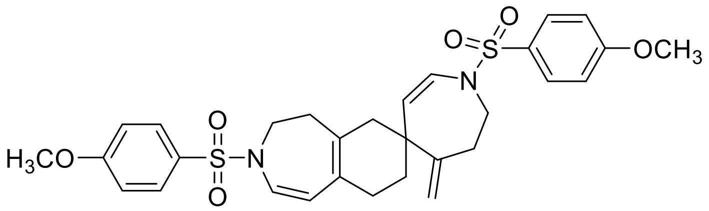

^{1}H NMR (400 MHz, $CDCl_3$)

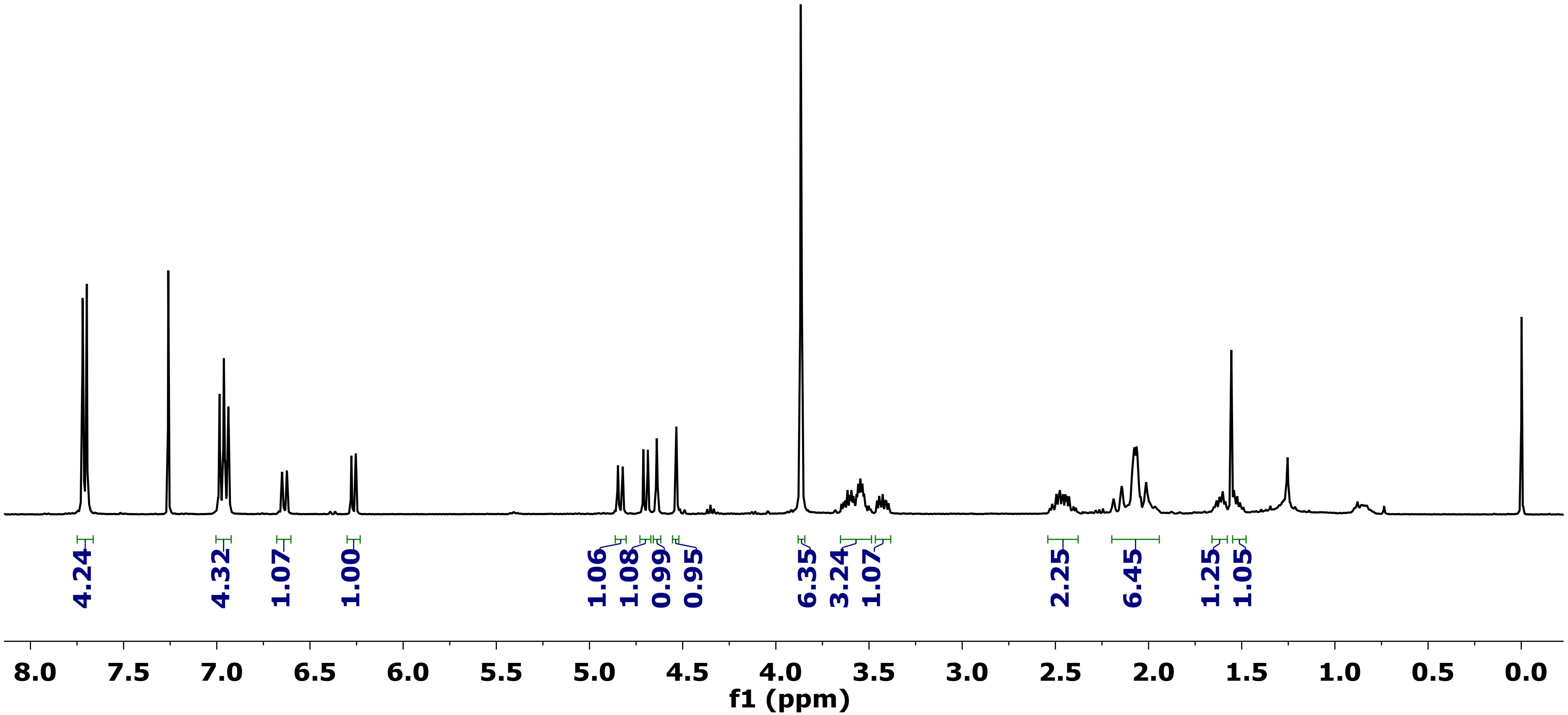

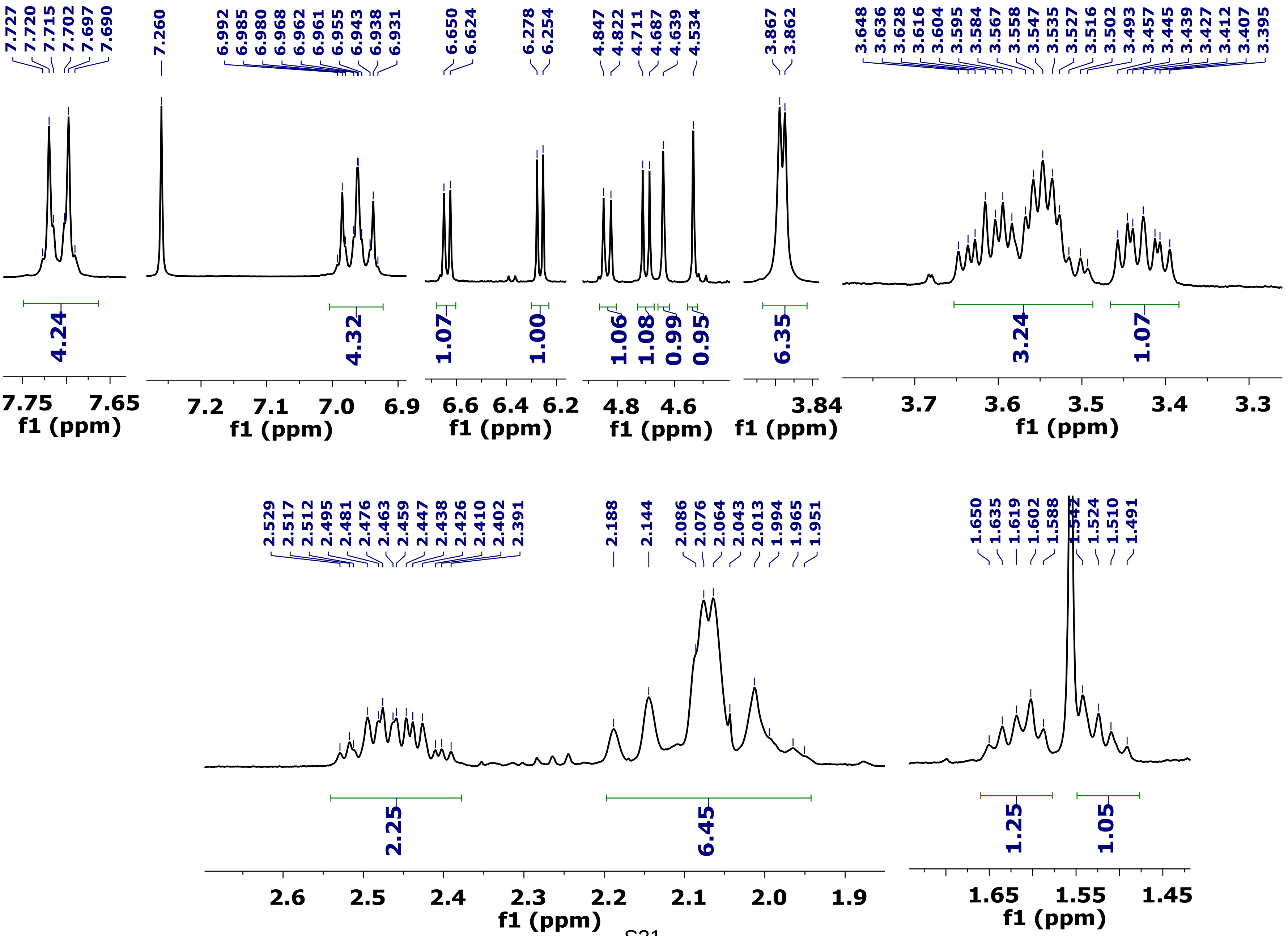
7.727
7.720
7.715
7.702
7.697
7.690
7.260
6.992
6.985
6.980
6.968
6.962
6.961
6.955
6.943
6.938
6.931
6.650
6.624
6.278
6.254
4.847
4.822
4.711
4.687
4.639
4.534
3.867
3.862
3.648
3.636
3.628
3.616
3.604
3.595
3.584
3.567
3.558
3.547
3.535
3.527
3.516
3.502
3.493
3.457
3.445
3.439
3.427
3.412
3.407
3.395
4.24
4.32
1.07
1.00
1.06
1.08
0.99
0.95
6.35
3.24
1.07
7.75
7.65
f1 (ppm)
7.2
7.1
7.0
6.9
f1 (ppm)
6.6
6.4
6.2
f1 (ppm)
4.8
4.6
f1 (ppm)
3.84
f1 (ppm)
3.7
3.6
3.5
3.4
3.3
f1 (ppm)
2.529
2.517
2.512
2.495
2.481
2.476
2.463
2.459
2.447
2.438
2.426
2.410
2.402
2.391
2.188
2.144
2.086
2.076
2.064
2.043
2.013
1.994
1.965
1.951
1.650
1.635
1.619
1.602
1.588
1.542
1.524
1.510
1.491
2.25
6.45
1.25
1.05
2.6
2.5
2.4
2.3
2.2
2.1
2.0
1.9
f1 (ppm)
1.65
1.55
1.45
f1 (ppm)

$^{13}C\{^1H\}$ NMR (101 MHz, $CDCl_3$)

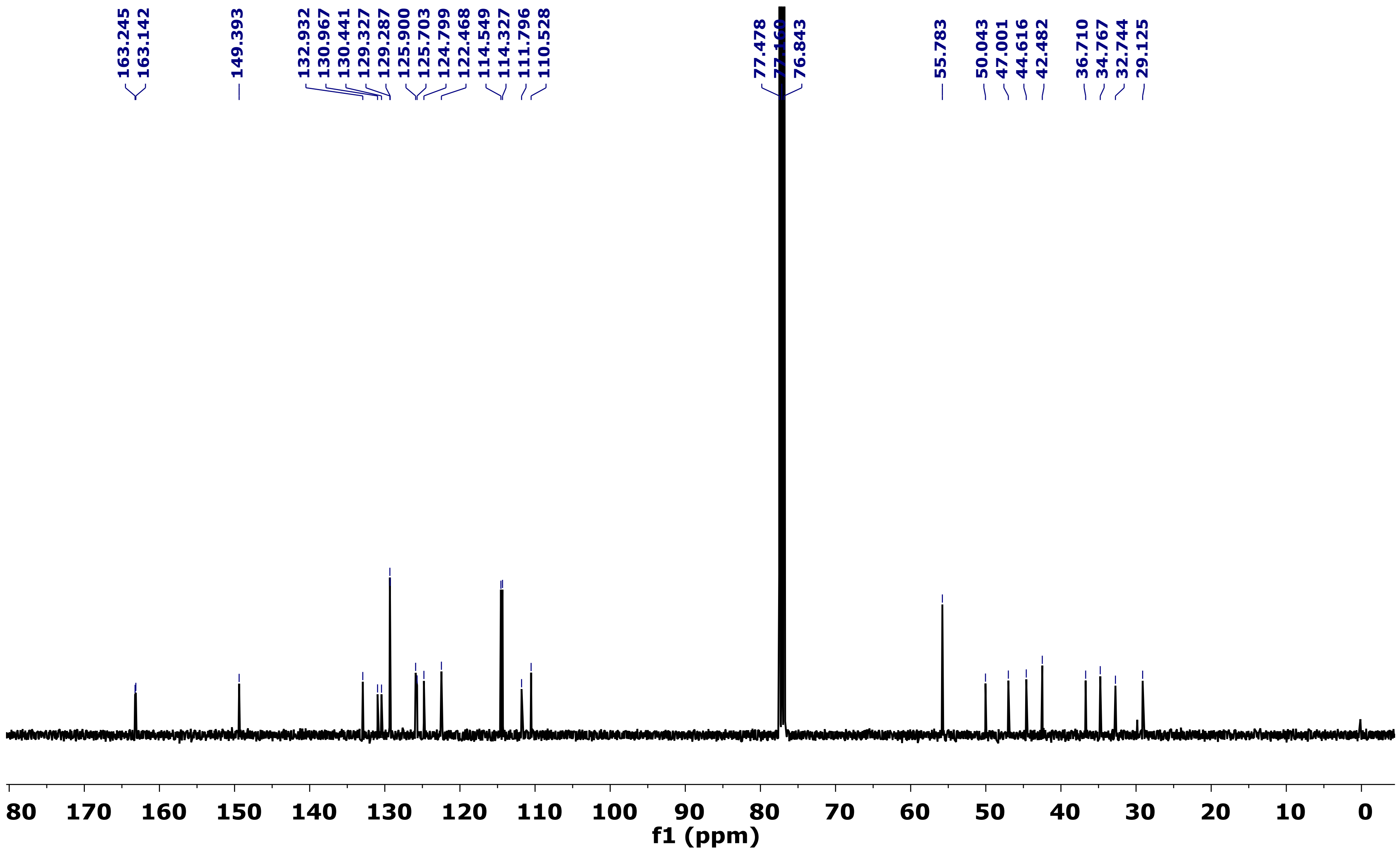

Product 2c

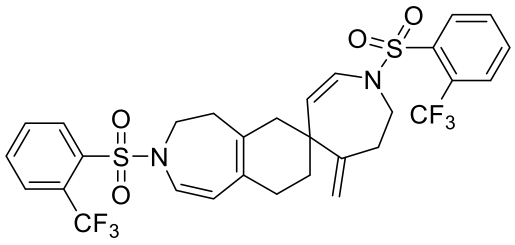

^{1}H NMR (400 MHz, $CDCl_3$)

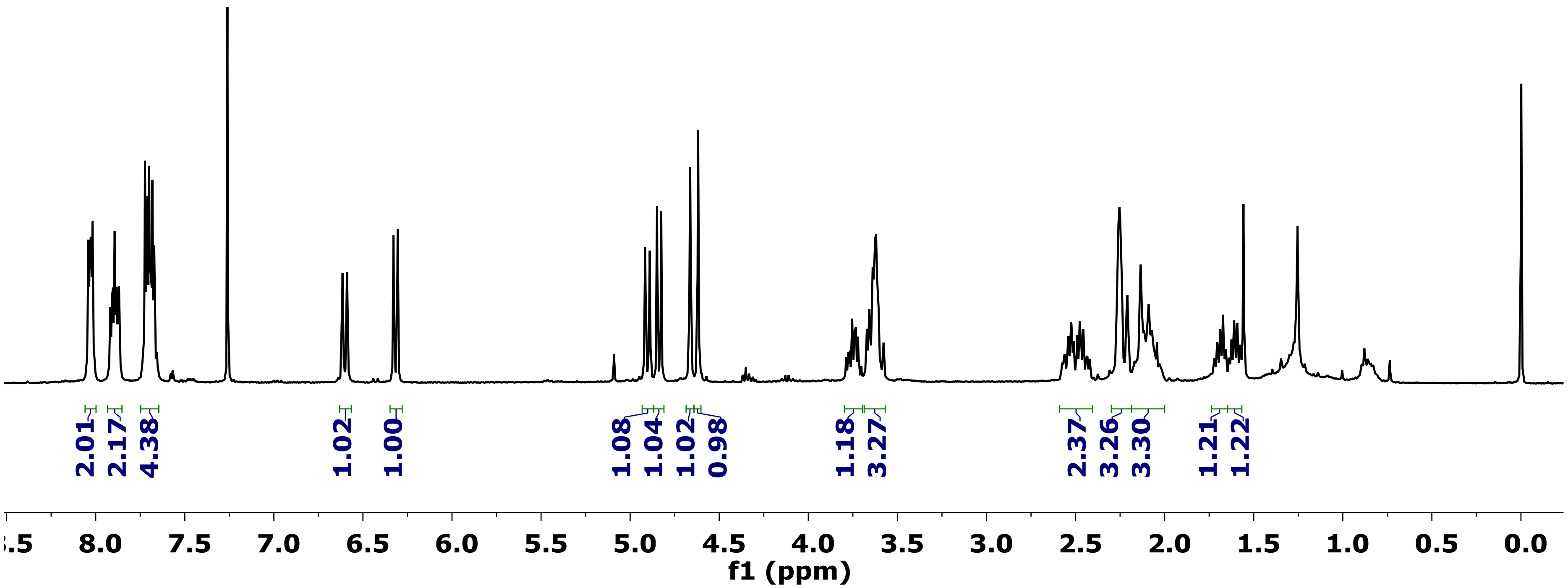

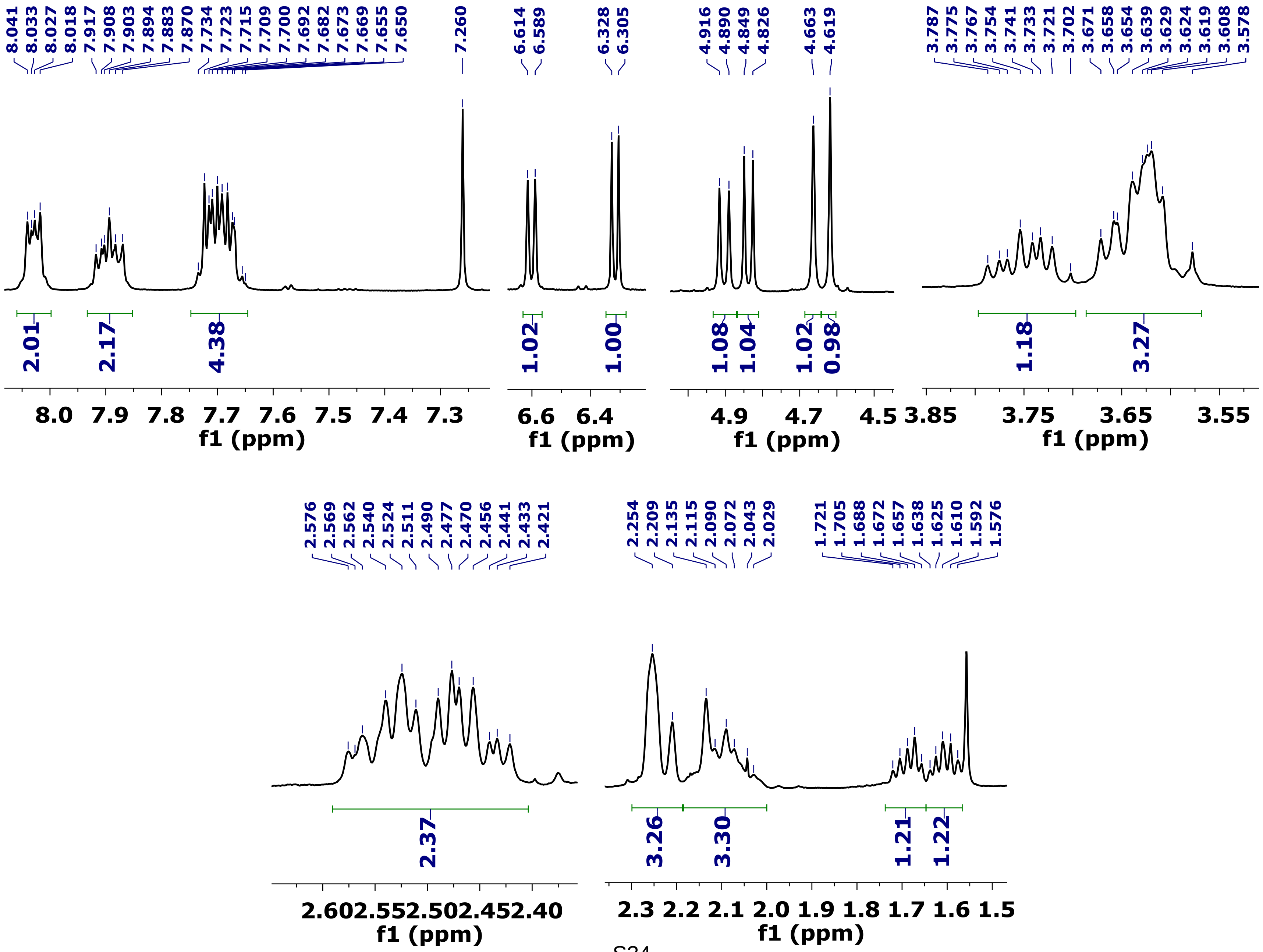
8.041 8.033 8.027 8.018 7.917 7.908 7.903 7.894 7.883 7.870 7.734 7.723 7.715 7.709 7.700 7.692 7.682 7.673 7.669 7.655 7.650 7.260
2.01 2.17 4.38
8.0 7.9 7.8 7.7 7.6 7.5 7.4 7.3
f1 (ppm)
6.614 6.589 6.328 6.305
1.02 1.00
6.6 6.4
f1 (ppm)
4.916 4.890 4.849 4.826 4.663 4.619
1.08 1.04 1.02 0.98
4.9 4.7 4.5
f1 (ppm)
3.787 3.775 3.767 3.754 3.741 3.733 3.721 3.702 3.671 3.658 3.654 3.639 3.629 3.624 3.619 3.608 3.578
1.18 3.27
3.85 3.75 3.65 3.55
f1 (ppm)
2.576 2.569 2.562 2.540 2.524 2.511 2.490 2.477 2.470 2.456 2.441 2.433 2.421
2.37
2.60 2.55 2.50 2.45 2.40
f1 (ppm)
2.254 2.209 2.135 2.115 2.090 2.072 2.043 2.029 1.721 1.705 1.688 1.672 1.657 1.638 1.625 1.610 1.592 1.576
3.26 3.30 1.21 1.22
2.3 2.2 2.1 2.0 1.9 1.8 1.7 1.6 1.5
f1 (ppm)

$^{13}C\{^{1}H\}$ NMR (101 MHz, $CDCl_3$)

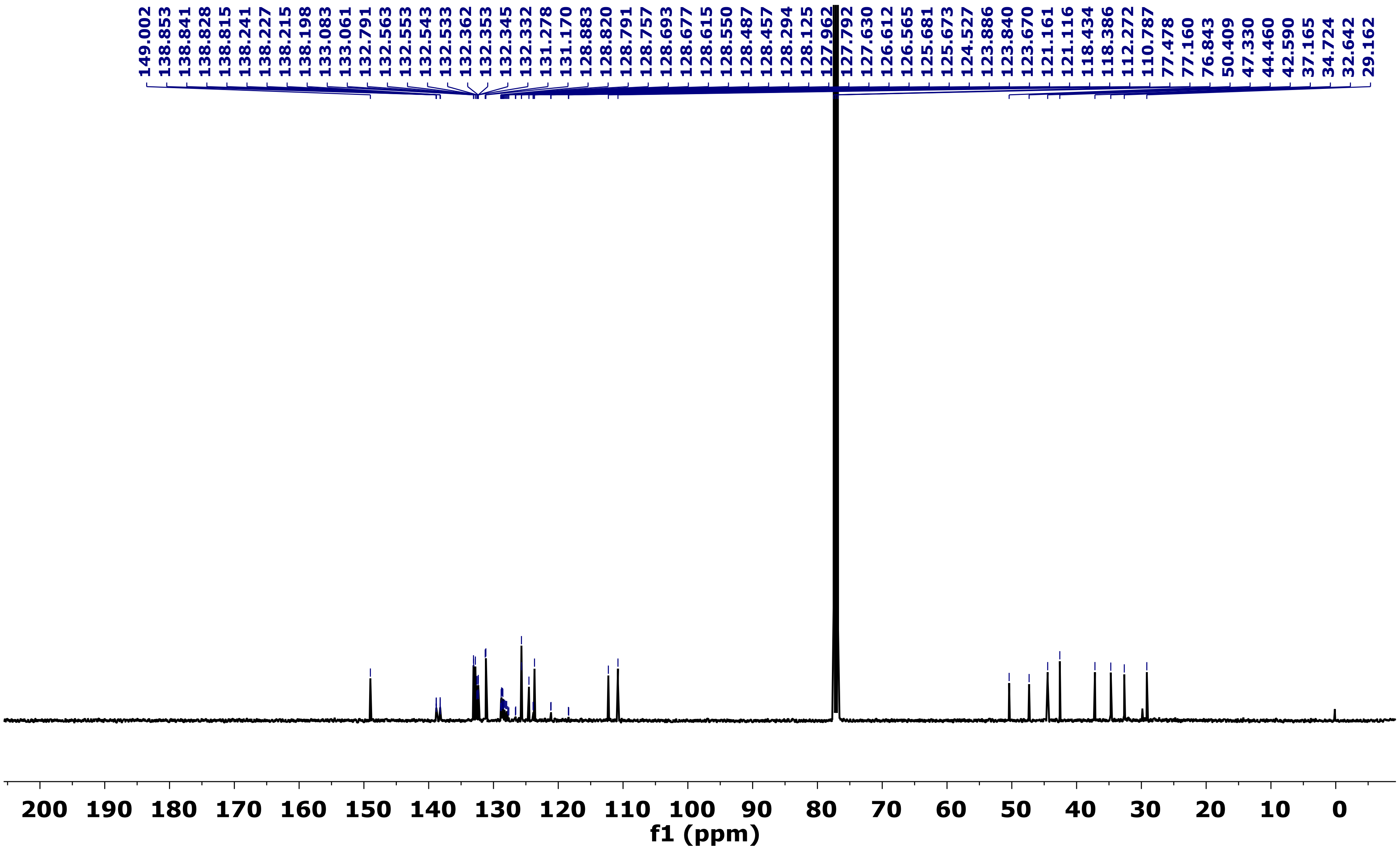

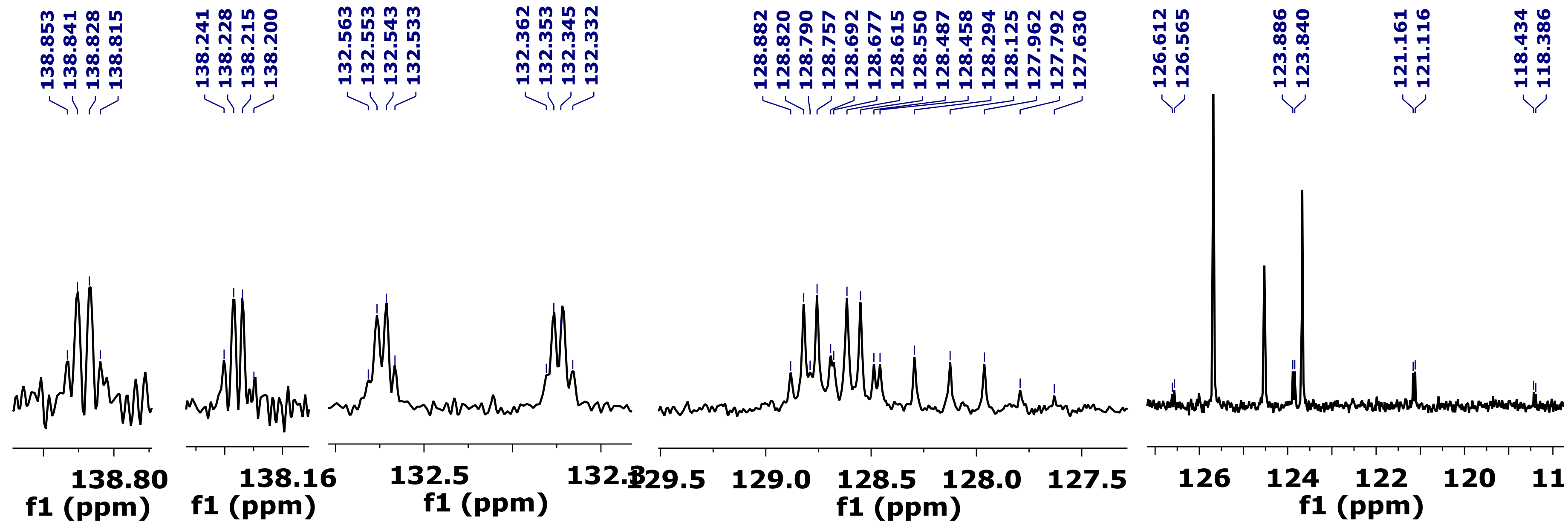
138.853
138.841
138.828
138.815
138.241
138.228
138.215
138.200
132.563
132.553
132.543
132.533
132.362
132.353
132.345
132.332
128.882
128.820
128.790
128.757
128.692
128.677
128.615
128.550
128.487
128.458
128.294
128.125
127.962
127.792
127.630
126.612
126.565
123.886
123.840
121.161
121.116
118.434
118.386
138.80
f1 (ppm)
138.16
f1 (ppm)
132.5
132.3
f1 (ppm)
129.5
129.0
128.5
128.0
127.5
f1 (ppm)
126
124
122
120
11
f1 (ppm)

Product 2d

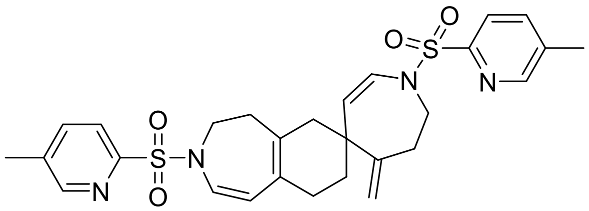

^{1}H NMR (400 MHz, $CDCl_3$)

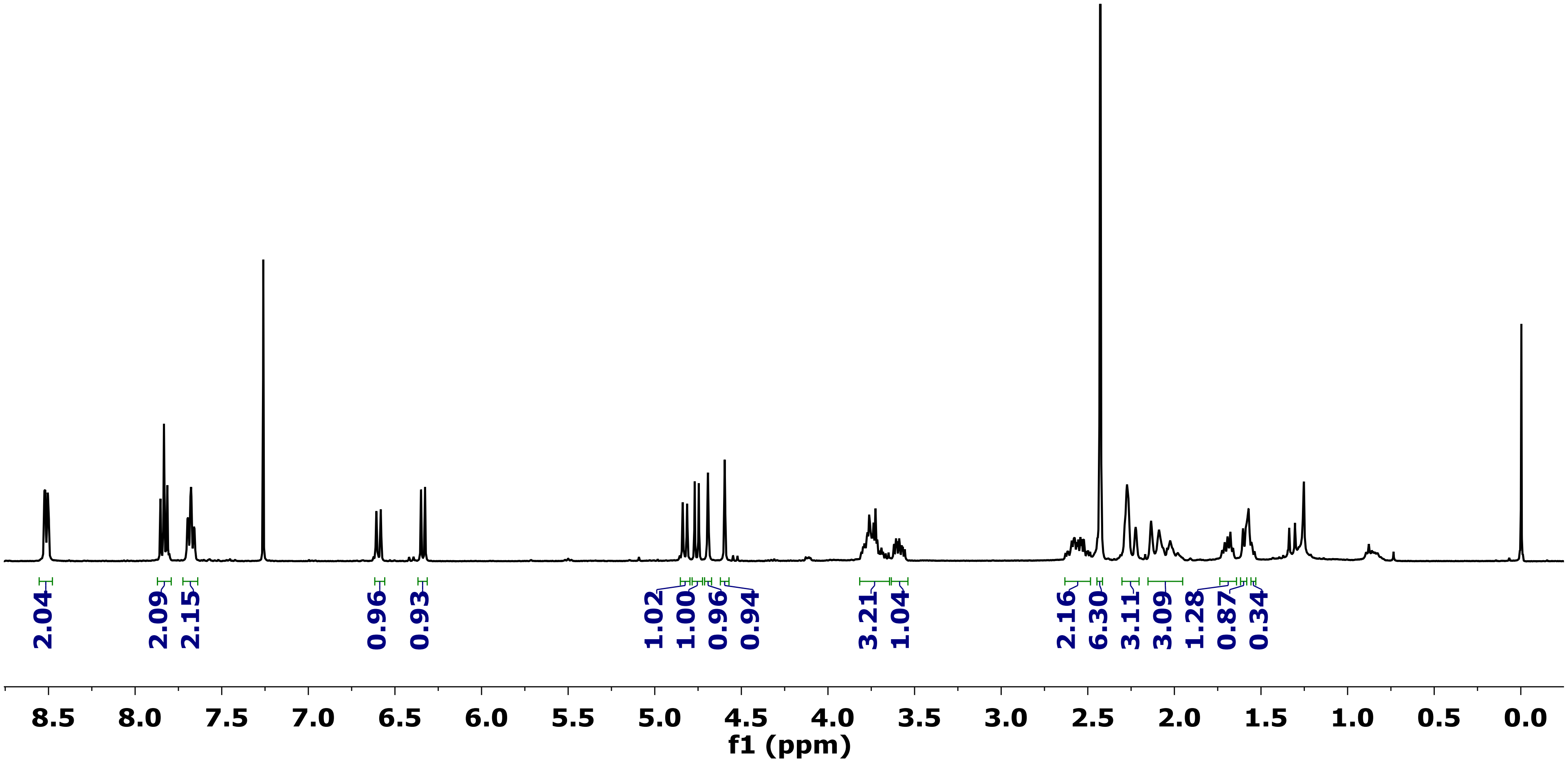

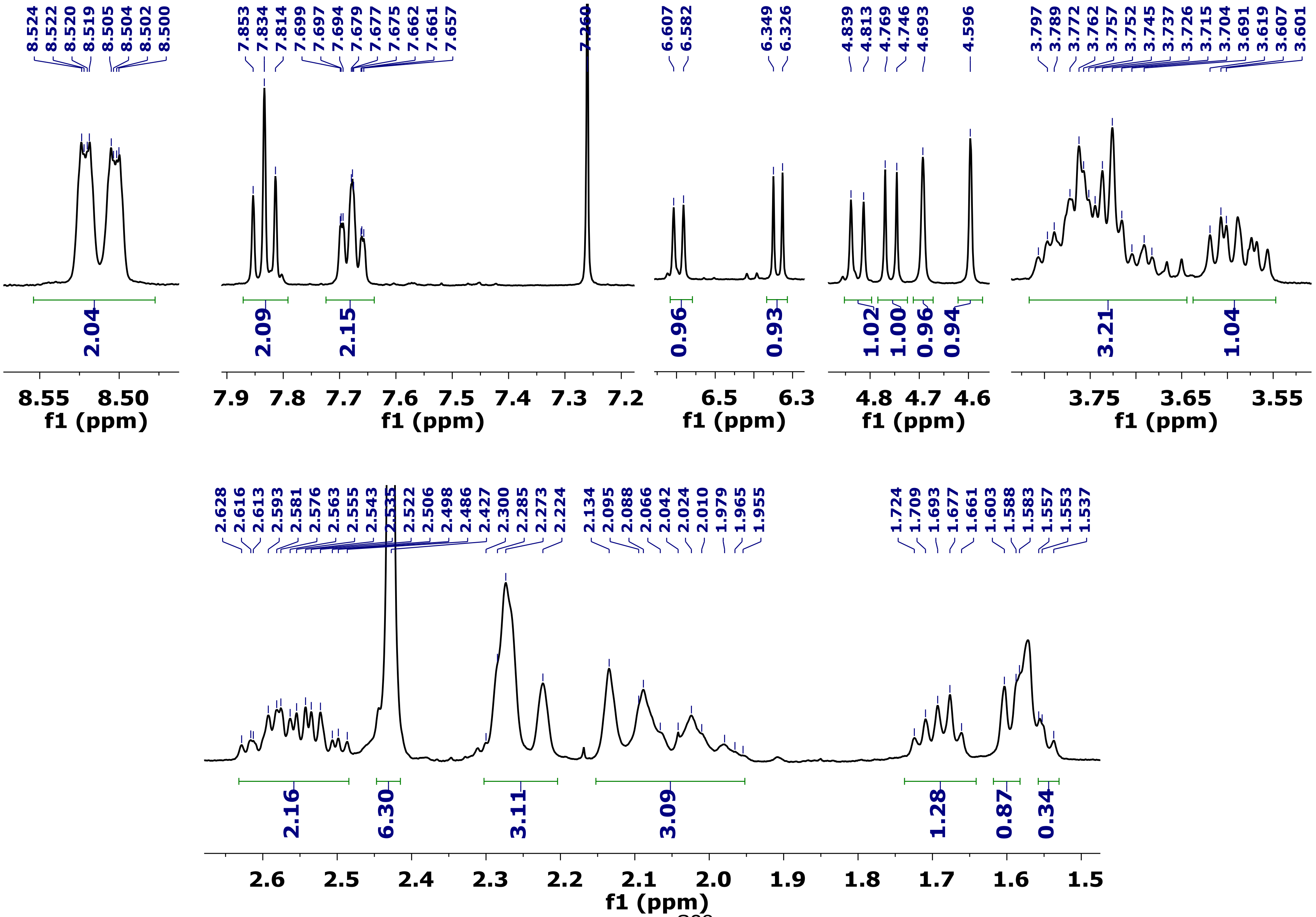
8.524 8.522 8.520 8.519 8.505 8.504 8.502 8.500
7.853 7.834 7.814 7.699 7.697 7.694 7.679 7.677 7.675 7.662 7.661 7.657
7.260
6.607 6.582 6.349 6.326
4.839 4.813 4.769 4.746 4.693 4.596
3.797 3.789 3.772 3.762 3.757 3.752 3.745 3.737 3.726 3.715 3.704 3.691 3.619 3.607 3.601
2.04 2.09 2.15 0.96 0.93 1.02 1.00 0.96 0.94 3.21 1.04
f1 (ppm)
2.628 2.616 2.613 2.593 2.581 2.576 2.563 2.555 2.543 2.535 2.522 2.506 2.498 2.486 2.427 2.300 2.285 2.273 2.224 2.134 2.095 2.088 2.066 2.042 2.024 2.010 1.979 1.965 1.955 1.724 1.709 1.693 1.677 1.661 1.603 1.588 1.583 1.557 1.553 1.537
2.16 6.30 3.11 3.09 1.28 0.87 0.34
f1 (ppm)

$^{13}C\{^1H\}$ NMR (101 MHz, $CDCl_3$)

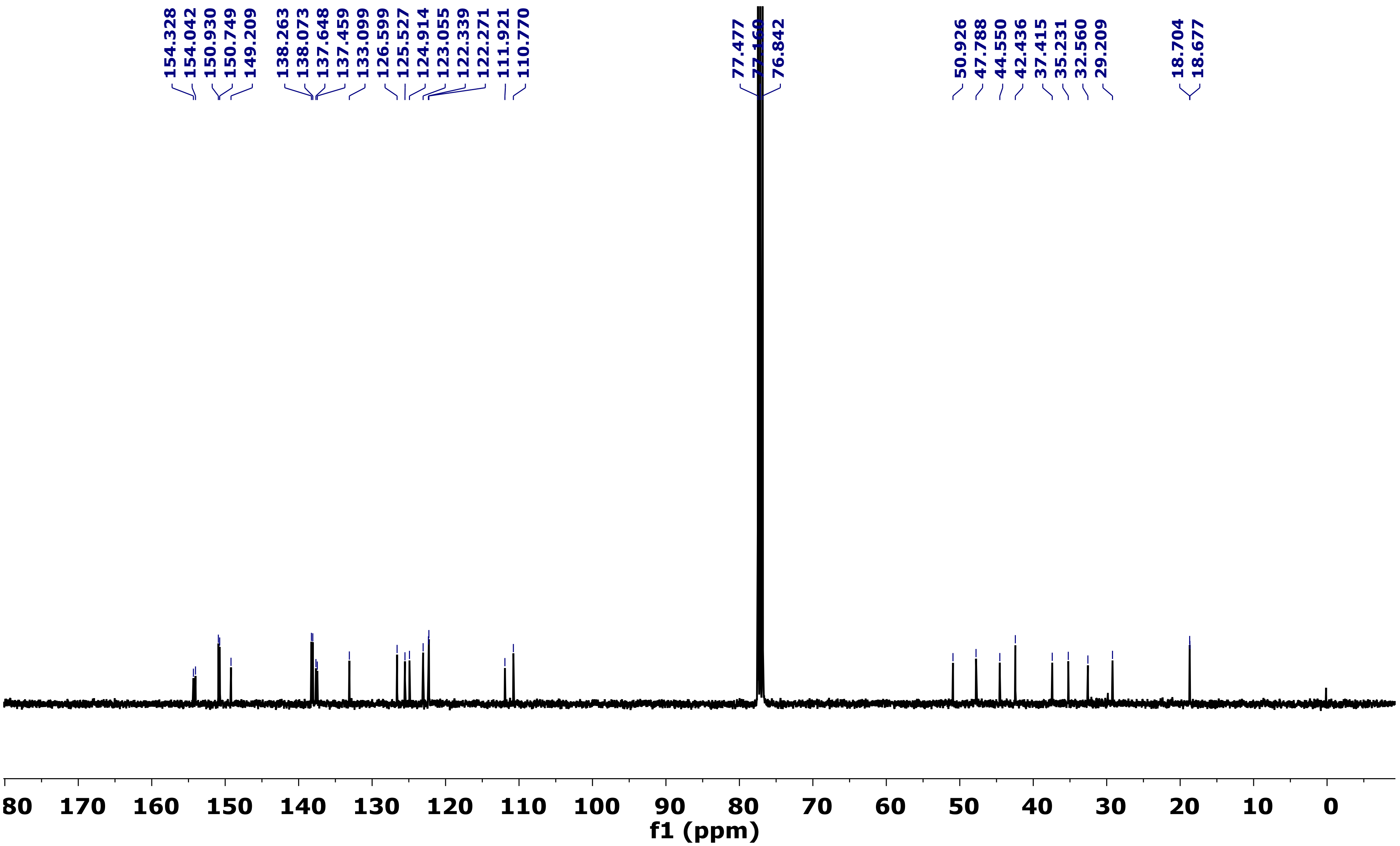

Product 2e

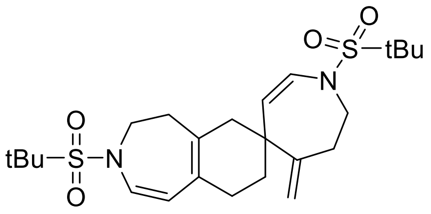

^{1}H NMR (400 MHz, $CDCl_3$)

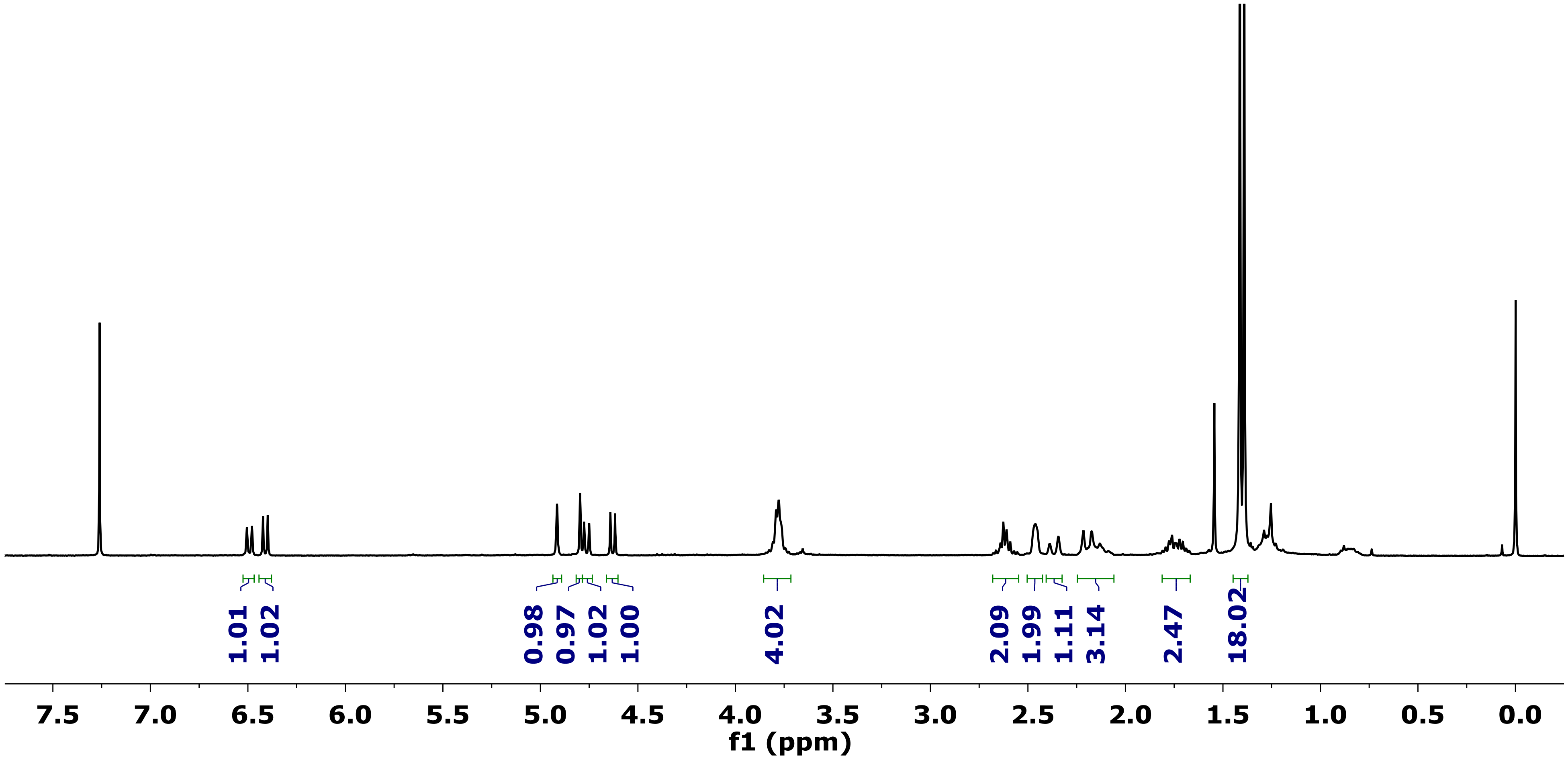

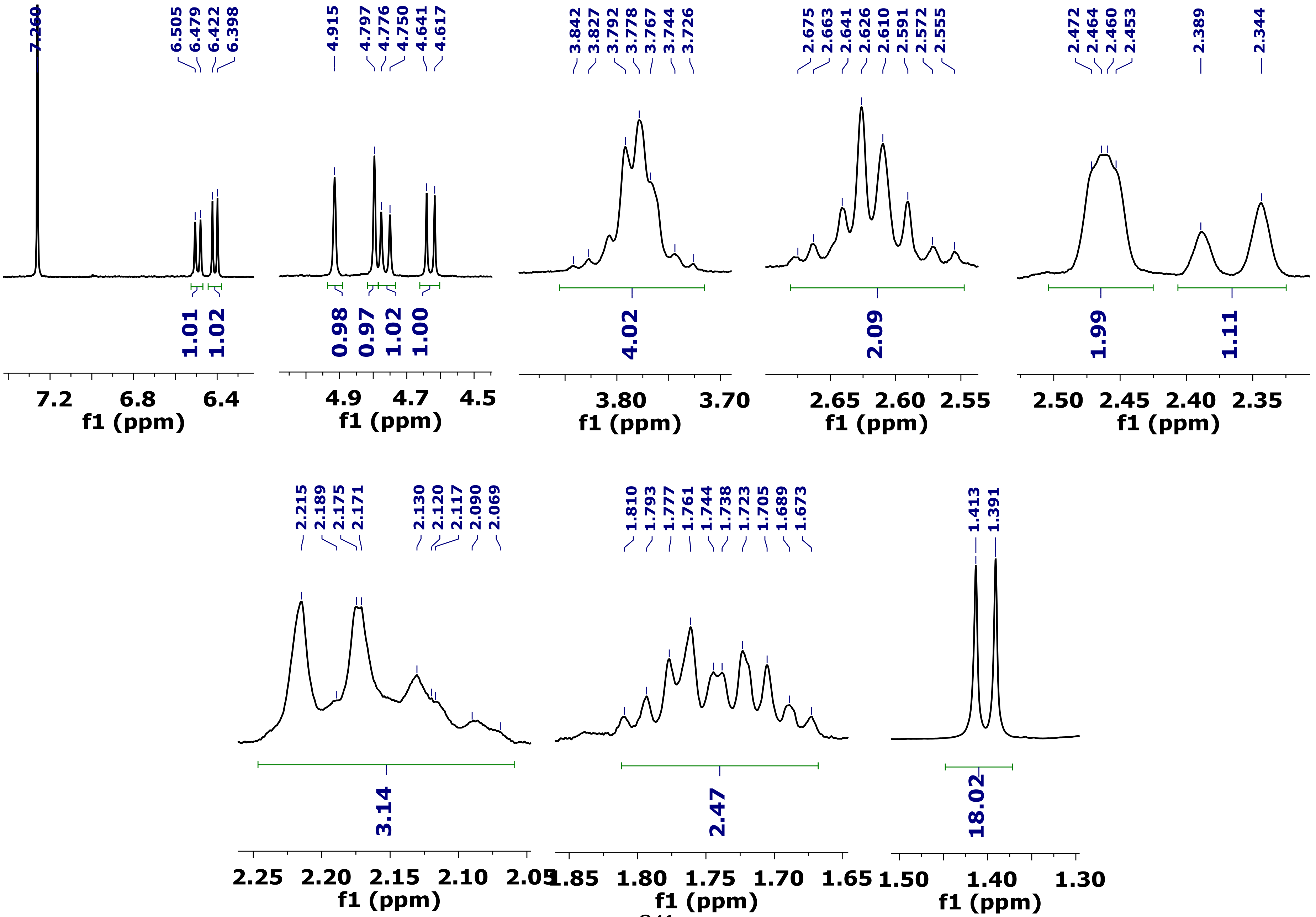
7.260
6.505
6.479
6.422
6.398
1.01
1.02
7.2
6.8
6.4
f1 (ppm)
4.915
4.797
4.776
4.750
4.641
4.617
0.98
0.97
1.02
1.00
4.9
4.7
4.5
f1 (ppm)
3.842
3.827
3.792
3.778
3.767
3.744
3.726
4.02
3.80
3.70
f1 (ppm)
2.675
2.663
2.641
2.626
2.610
2.591
2.572
2.555
2.09
2.65
2.60
2.55
f1 (ppm)
2.472
2.464
2.460
2.453
2.389
2.344
1.99
1.11
2.50
2.45
2.40
2.35
f1 (ppm)
2.215
2.189
2.175
2.171
2.130
2.120
2.117
2.090
2.069
3.14
2.25
2.20
2.15
2.10
2.05
f1 (ppm)
1.810
1.793
1.777
1.761
1.744
1.738
1.723
1.705
1.689
1.673
2.47
1.85
1.80
1.75
1.70
1.65
f1 (ppm)
1.413
1.391
18.02
1.50
1.40
1.30
f1 (ppm)

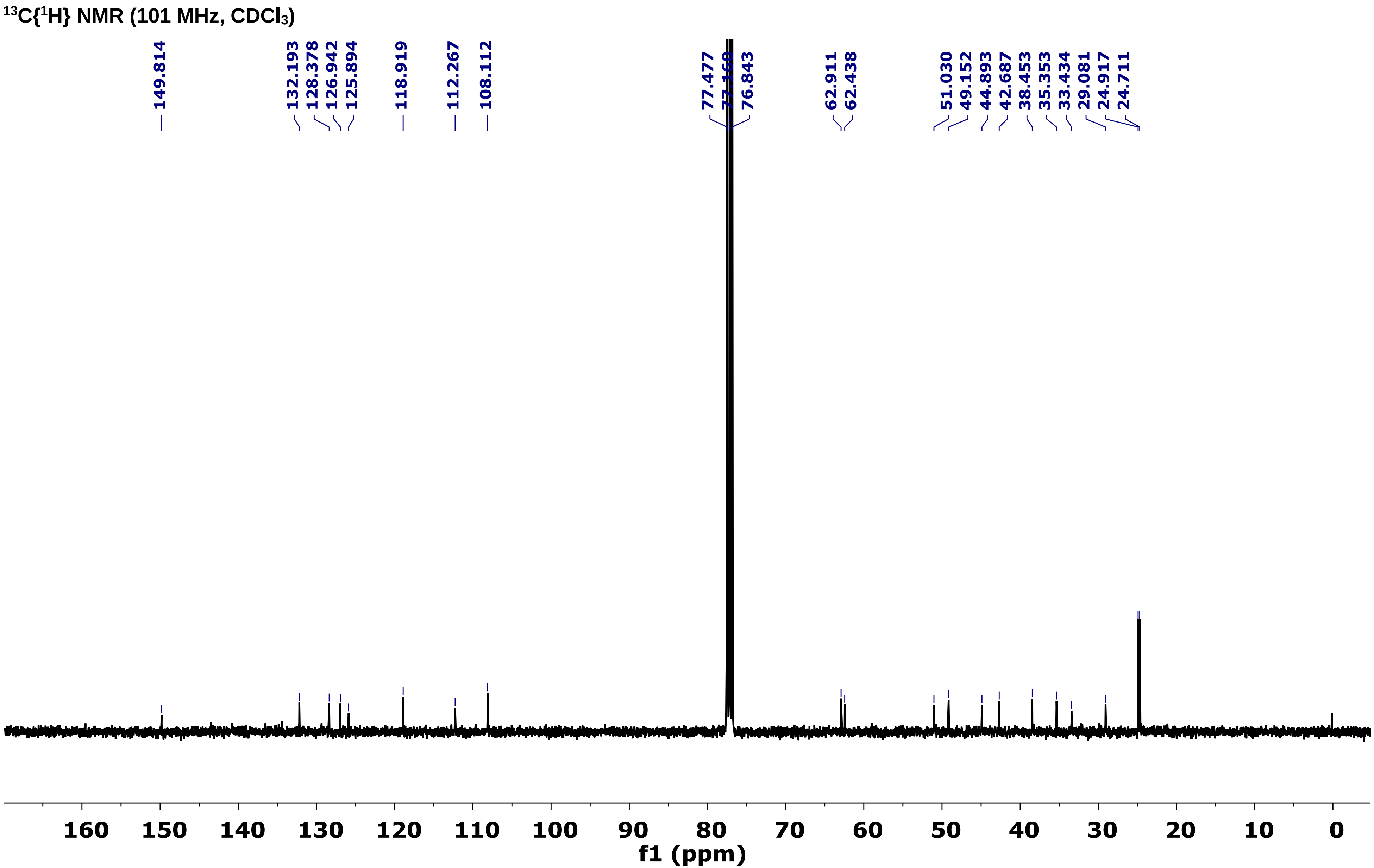

13C{1H} NMR (101 MHz, CDCl3)
149.814
132.193
128.378
126.942
125.894
118.919
112.267
108.112
77.477
77.160
76.843
62.911
62.438
51.030
49.152
44.893
42.687
38.453
35.353
33.434
29.081
24.917
24.711
160 150 140 130 120 110 100 90 80 70 60 50 40 30 20 10 0
f1 (ppm)

Product 2f

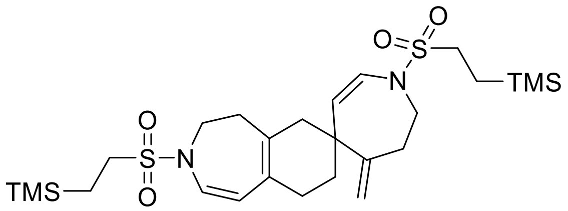

^{1}H NMR (400 MHz, $CDCl_3$)

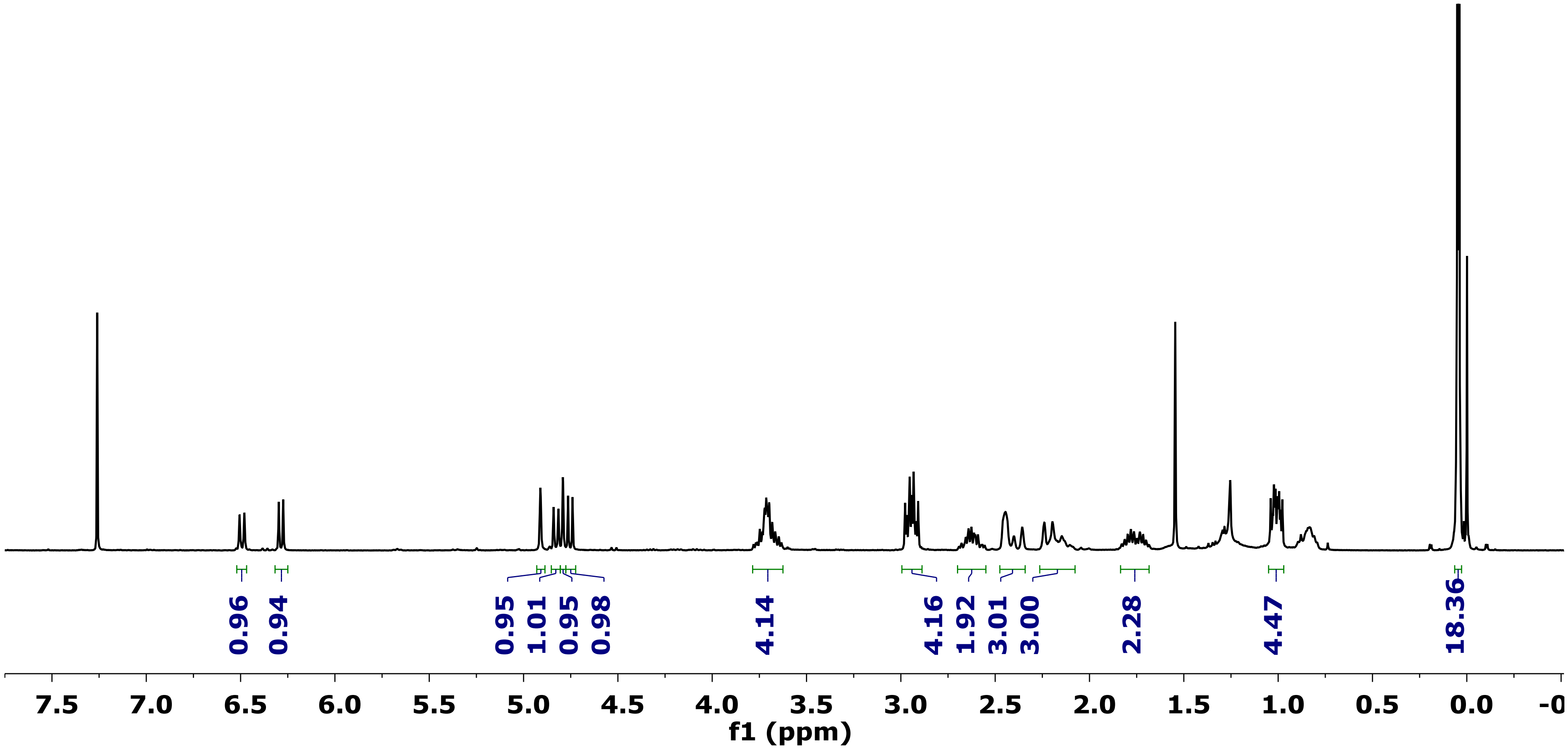

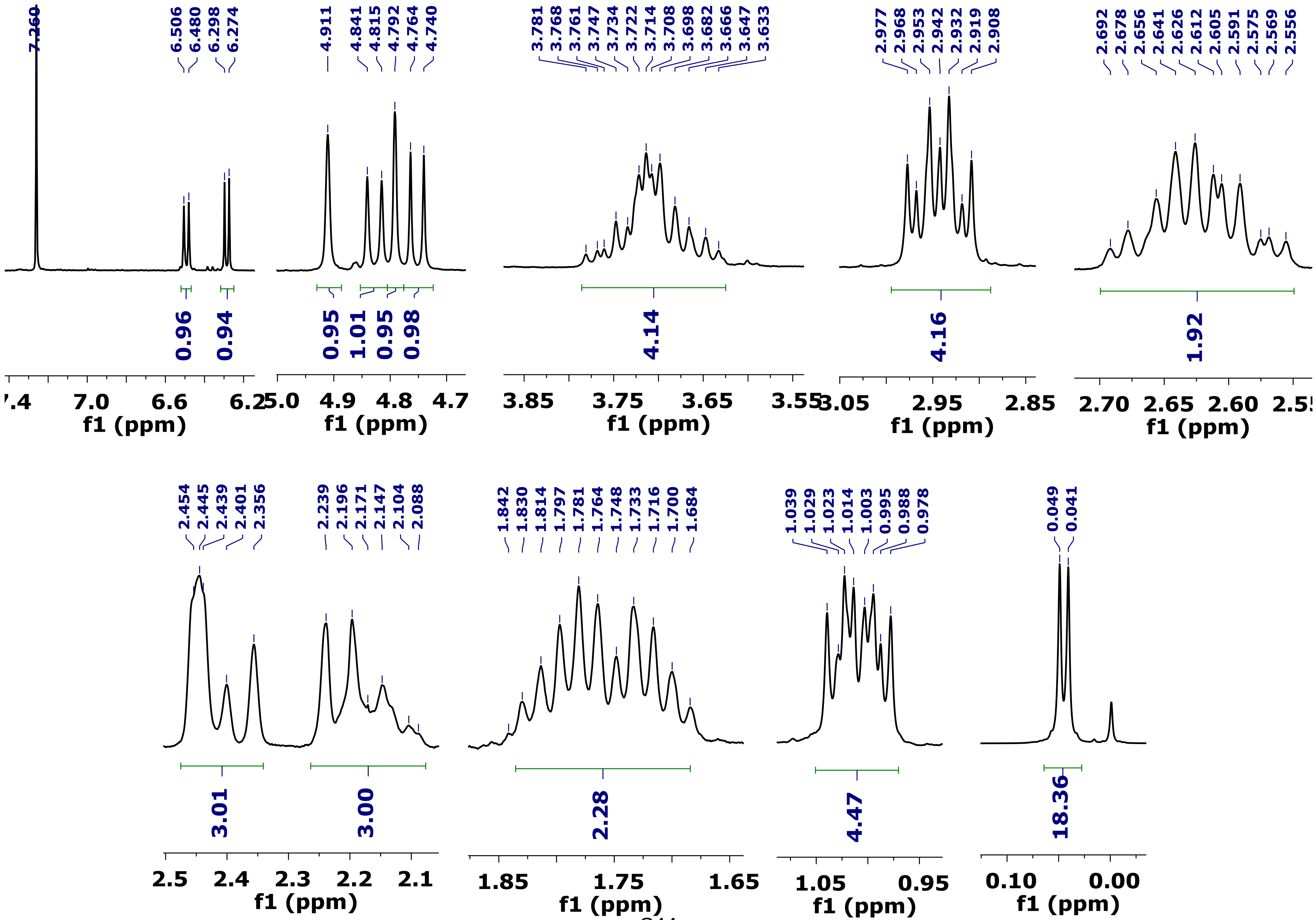
7.260
6.506 6.480 6.298 6.274
4.911 4.841 4.815 4.792 4.764 4.740
3.781 3.768 3.761 3.747 3.734 3.722 3.714 3.708 3.698 3.682 3.666 3.647 3.633
2.977 2.968 2.953 2.942 2.932 2.919 2.908
2.692 2.678 2.656 2.641 2.626 2.612 2.605 2.591 2.575 2.569 2.556
0.96 0.94 0.95 1.01 0.95 0.98 4.14 4.16 1.92
f1 (ppm)
2.454 2.445 2.439 2.401 2.356
2.239 2.196 2.171 2.147 2.104 2.088
1.842 1.830 1.814 1.797 1.781 1.764 1.748 1.733 1.716 1.700 1.684
1.039 1.029 1.023 1.014 1.003 0.995 0.988 0.978
0.049 0.041
3.01 3.00 2.28 4.47 18.36
f1 (ppm)

$^{13}C\{^1H\}$ NMR (101 MHz, $CDCl_3$)

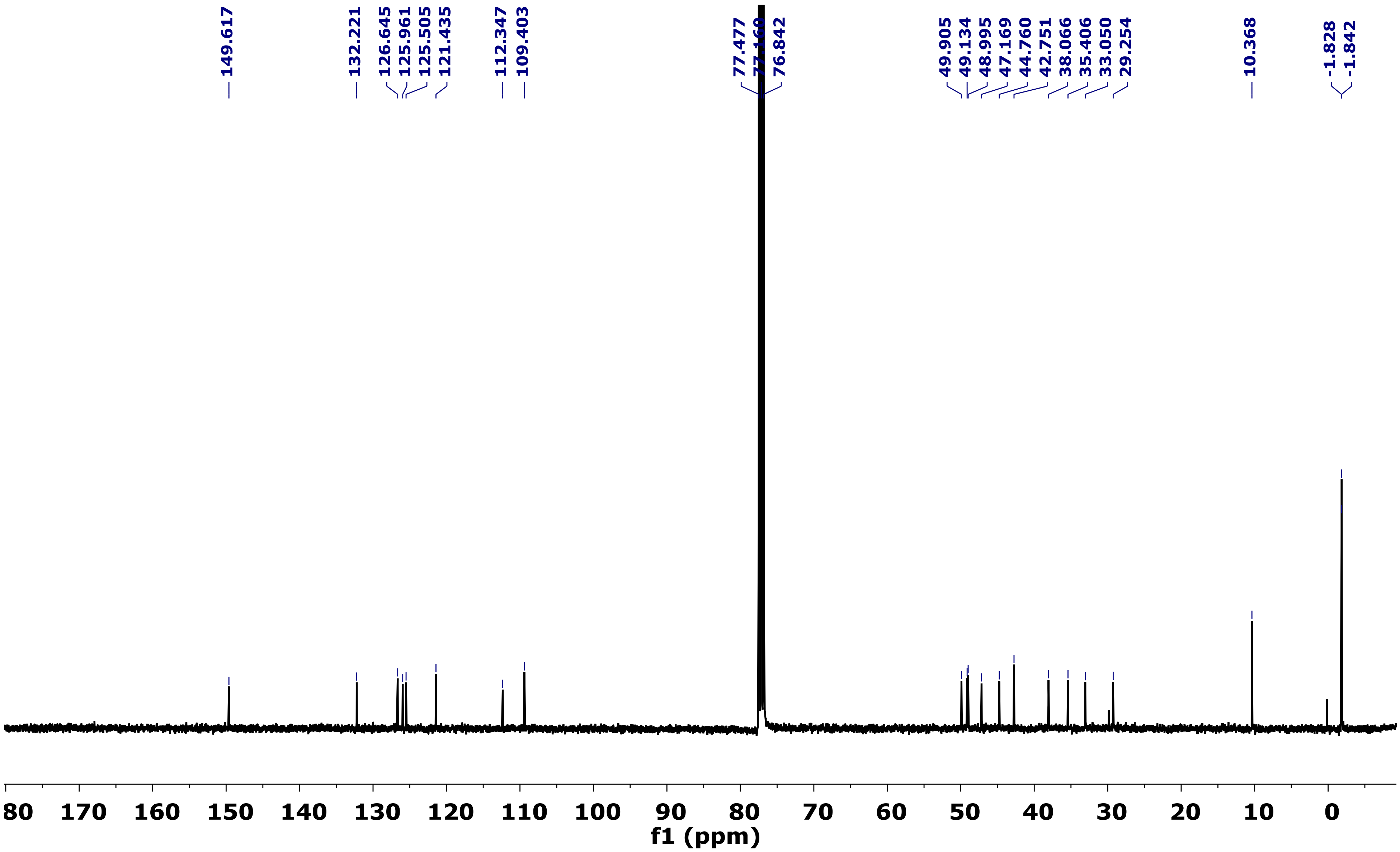

Product 2g

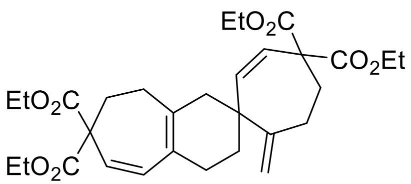

^{1}H NMR (400 MHz, $CDCl_3$)

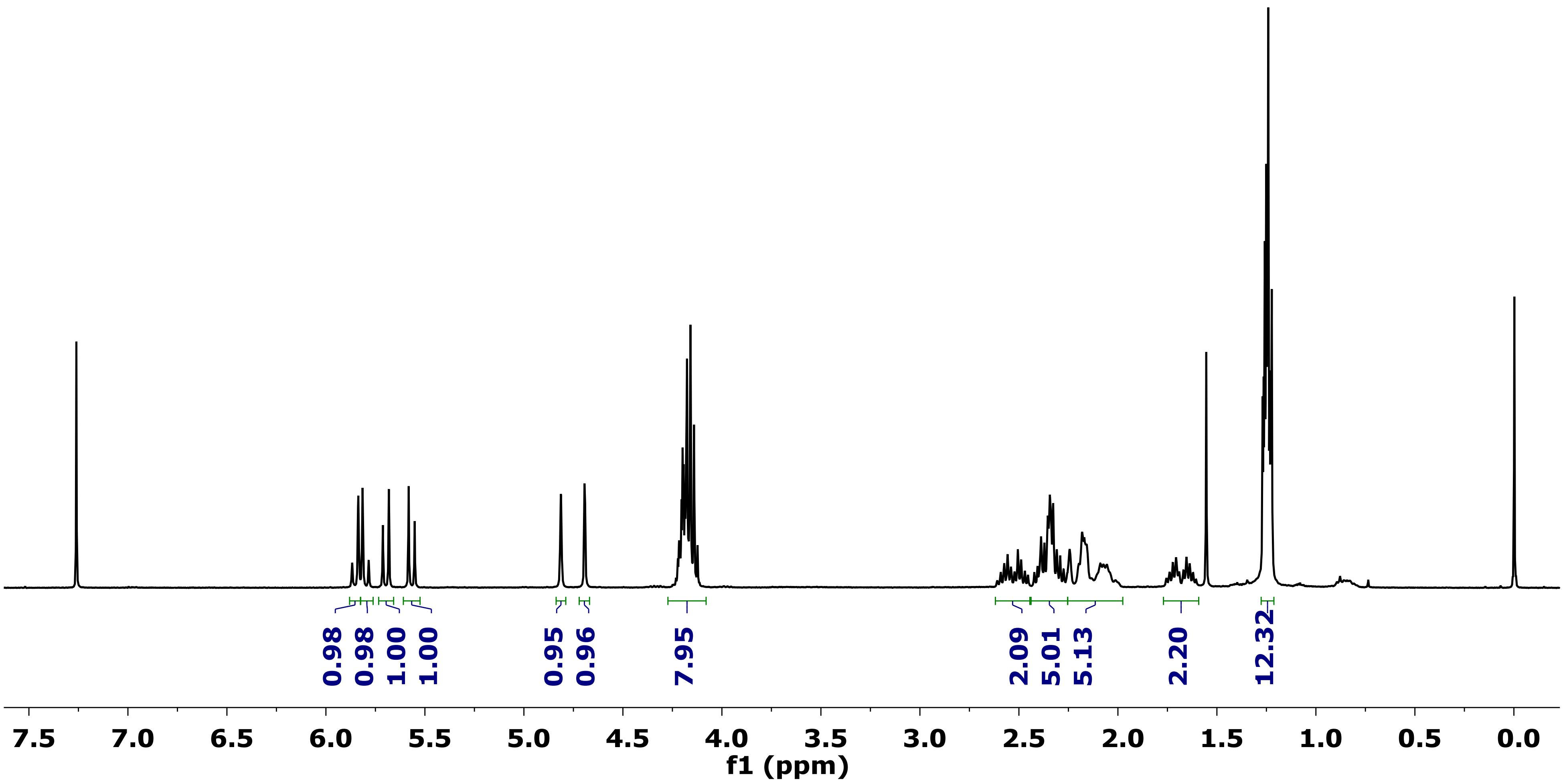

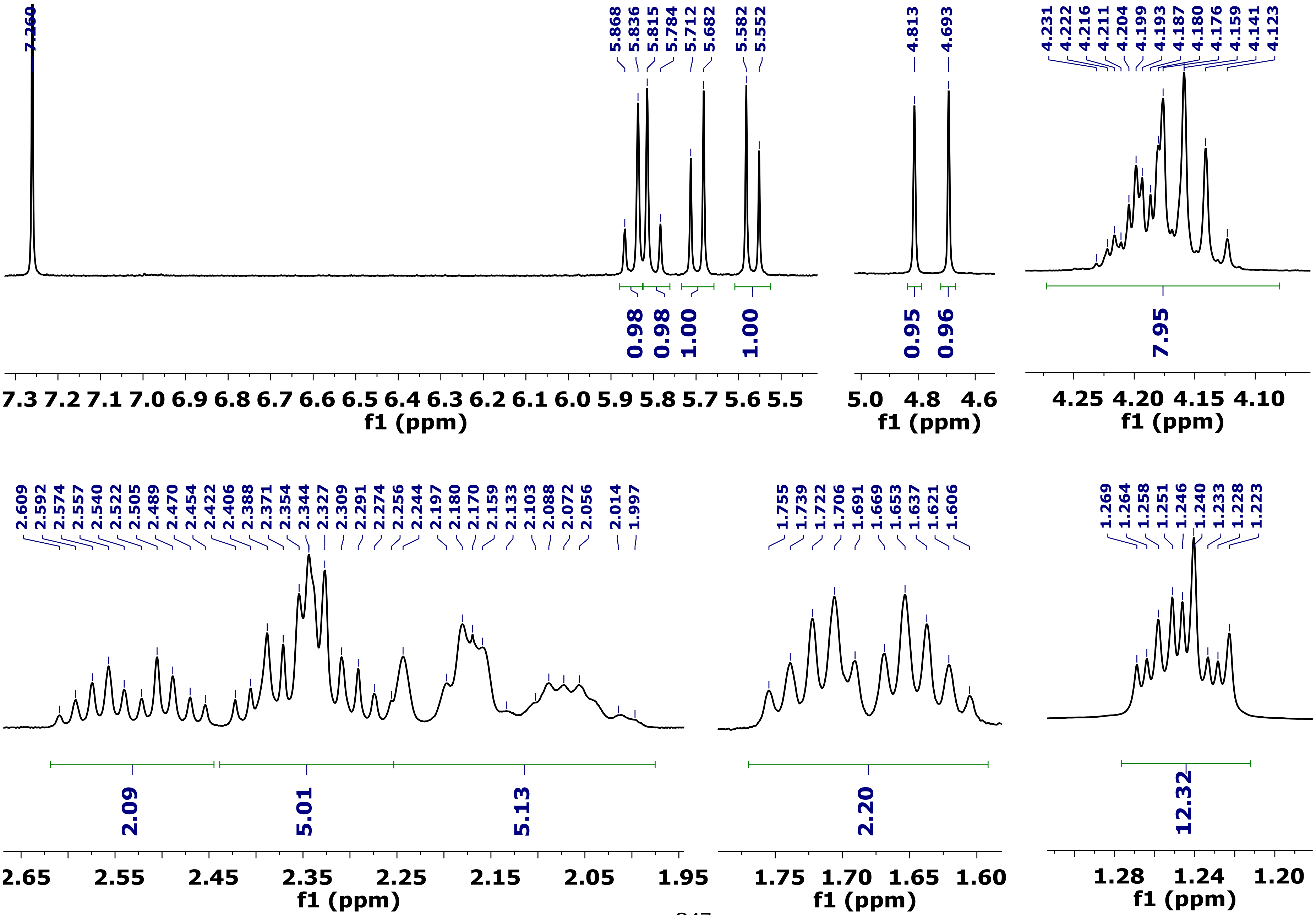
7.260
5.868 5.836 5.815 5.784 5.712 5.682 5.582 5.552
0.98 0.98 1.00 1.00
7.3 7.2 7.1 7.0 6.9 6.8 6.7 6.6 6.5 6.4 6.3 6.2 6.1 6.0 5.9 5.8 5.7 5.6 5.5
f1 (ppm)
4.813 4.693
0.95 0.96
5.0 4.8 4.6
f1 (ppm)
4.231 4.222 4.216 4.211 4.204 4.199 4.193 4.187 4.180 4.176 4.159 4.141 4.123
7.95
4.25 4.20 4.15 4.10
f1 (ppm)
2.609 2.592 2.574 2.557 2.540 2.522 2.505 2.489 2.470 2.454 2.422 2.406 2.388 2.371 2.354 2.344 2.327 2.309 2.291 2.274 2.256 2.244 2.197 2.180 2.170 2.159 2.133 2.103 2.088 2.072 2.056 2.014 1.997
2.09 5.01 5.13
2.65 2.55 2.45 2.35 2.25 2.15 2.05 1.95
f1 (ppm)
1.755 1.739 1.722 1.706 1.691 1.669 1.653 1.637 1.621 1.606
2.20
1.75 1.70 1.65 1.60
f1 (ppm)
1.269 1.264 1.258 1.251 1.246 1.240 1.233 1.228 1.223
12.32
1.28 1.24 1.20
f1 (ppm)

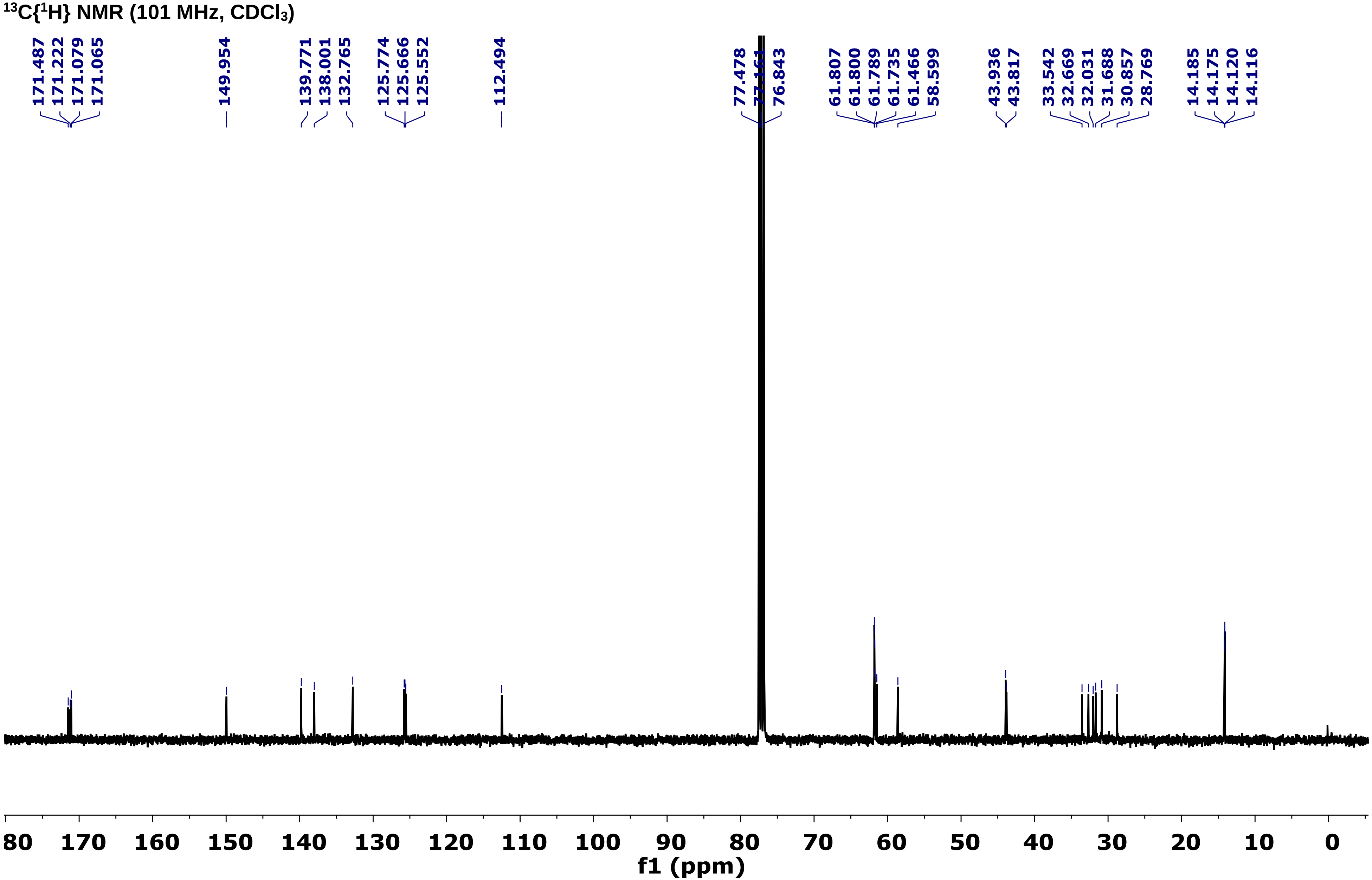

$^{13}C\{^1H\}$ NMR (101 MHz, $CDCl_3$)
171.487
171.222
171.079
171.065
149.954
139.771
138.001
132.765
125.774
125.666
125.552
112.494
77.478
77.161
76.843
61.807
61.800
61.789
61.735
61.466
58.599
43.936
43.817
33.542
32.669
32.031
31.688
30.857
28.769
14.185
14.175
14.120
14.116
80
170
160
150
140
130
120
110
100
90
80
70
60
50
40
30
20
10
0
f1 (ppm)

Product 2h

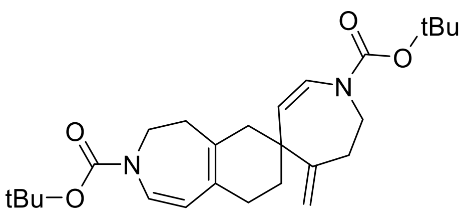

^{1}H NMR (400 MHz, $CDCl_3$)

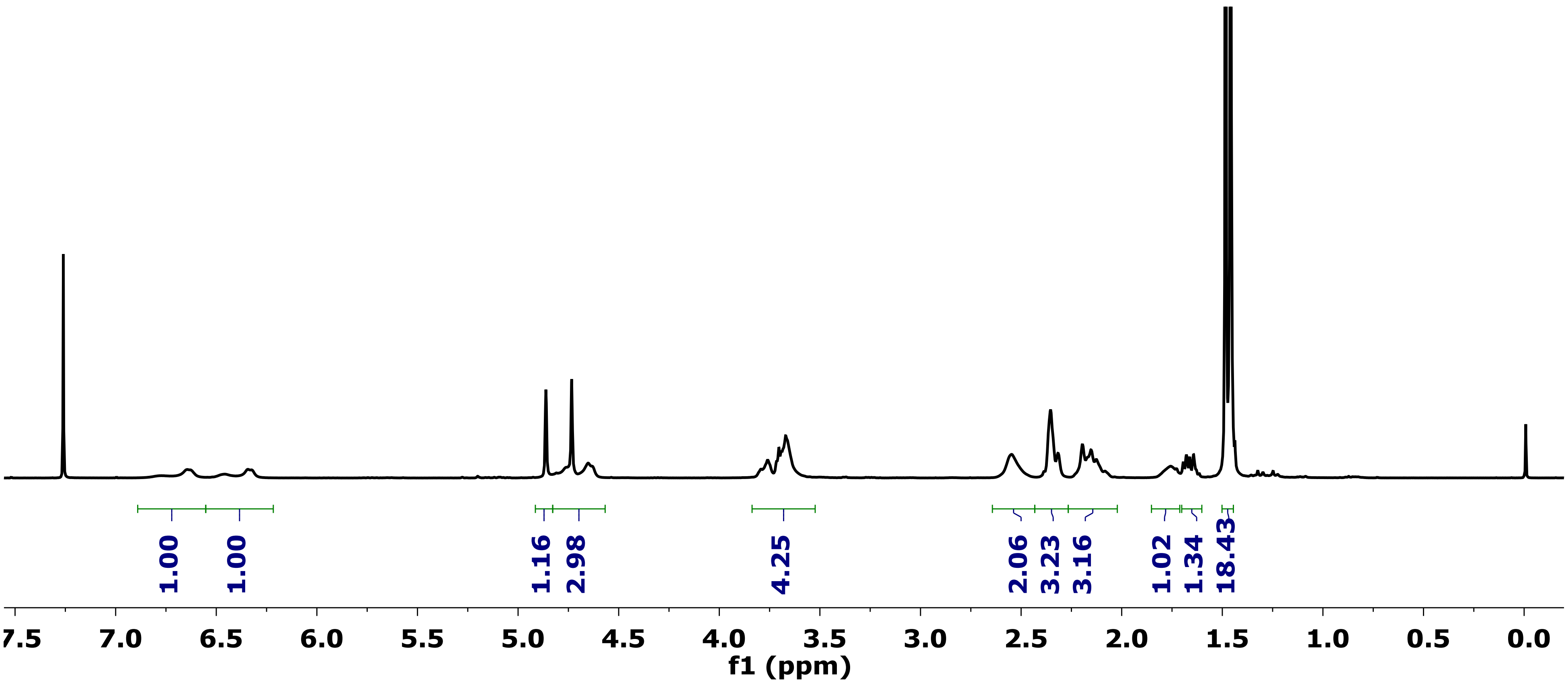

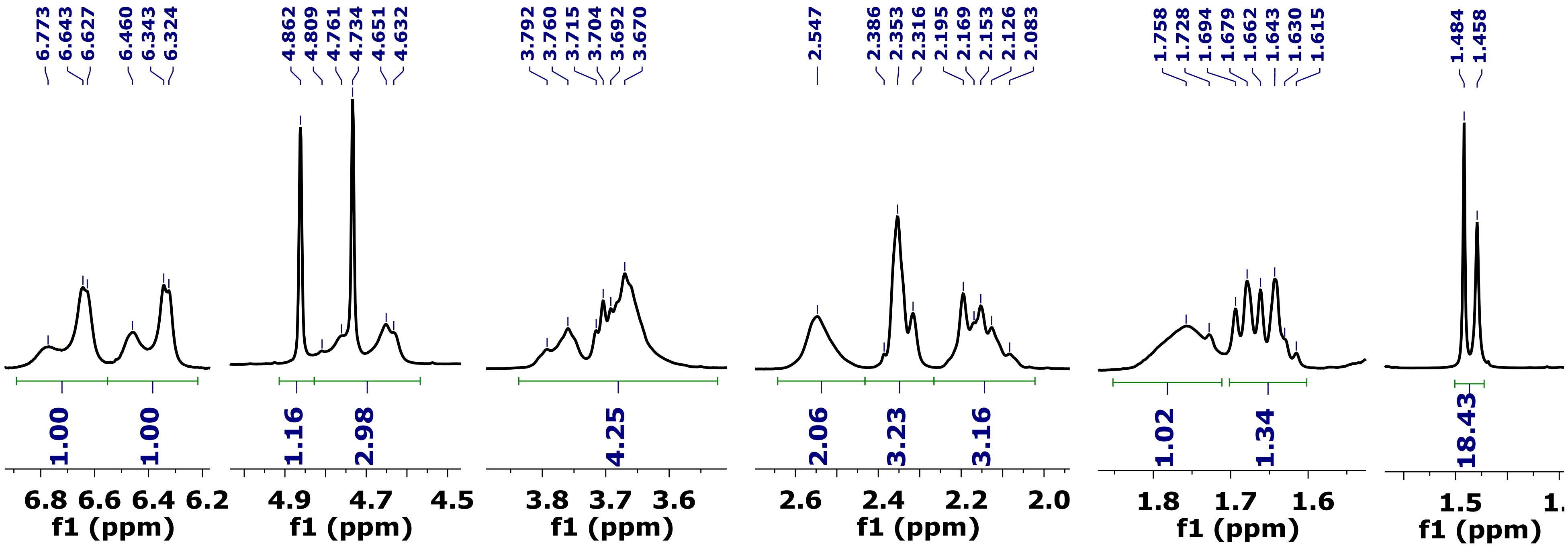
6.773
6.643
6.627
6.460
6.343
6.324
4.862
4.809
4.761
4.734
4.651
4.632
3.792
3.760
3.715
3.704
3.692
3.670
2.547
2.386
2.353
2.316
2.195
2.169
2.153
2.126
2.083
1.758
1.728
1.694
1.679
1.662
1.643
1.630
1.615
1.484
1.458
1.00
1.00
1.16
2.98
4.25
2.06
3.23
3.16
1.02
1.34
18.43
f1 (ppm)

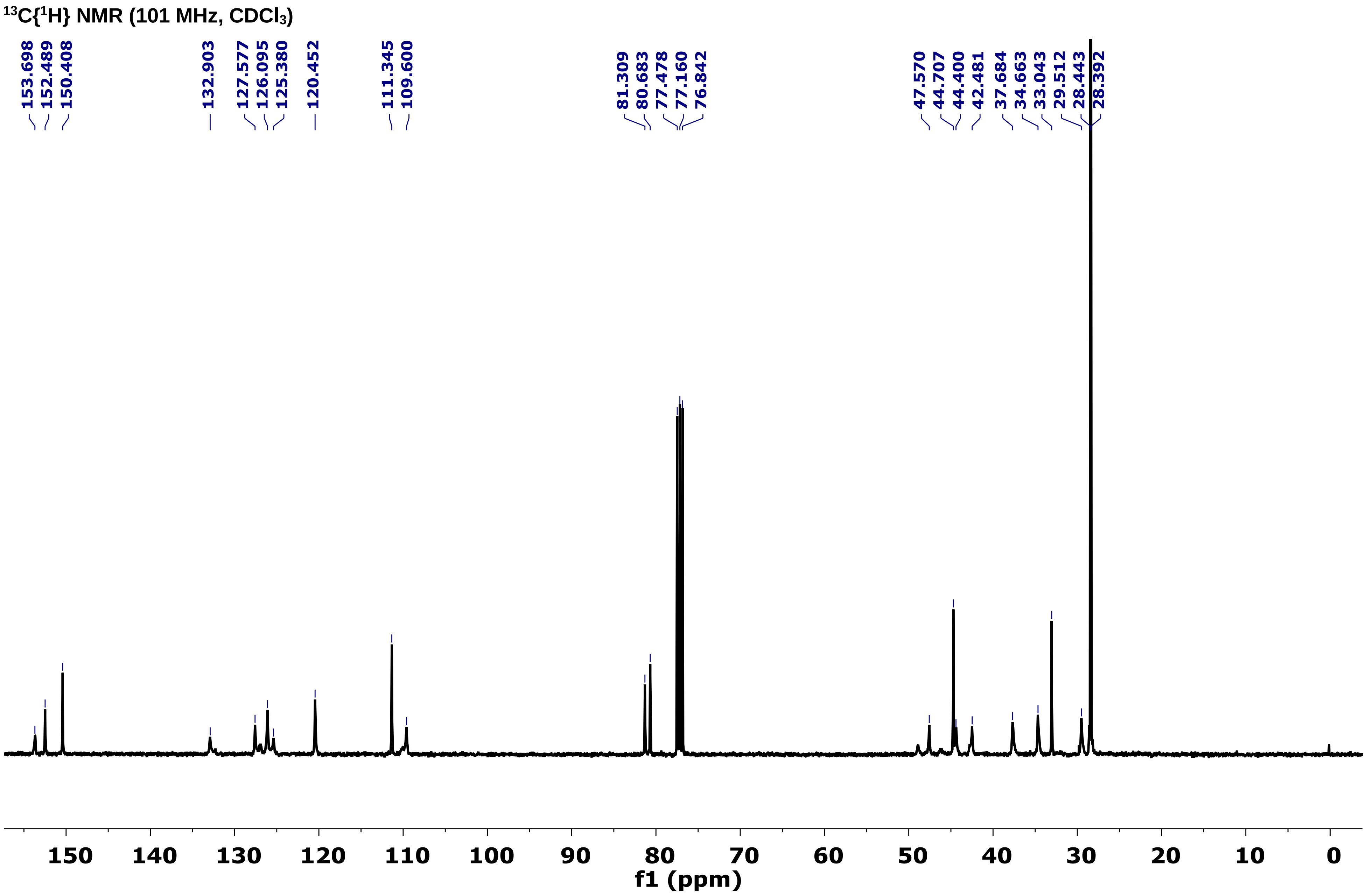

$^{13}C\{^1H\}$ NMR (101 MHz, $CDCl_3$)
153.698
152.489
150.408
132.903
127.577
126.095
125.380
120.452
111.345
109.600
81.309
80.683
77.478
77.160
76.842
47.570
44.707
44.400
42.481
37.684
34.663
33.043
29.512
28.443
28.392
150
140
130
120
110
100
90
80
70
60
50
40
30
20
10
0
f1 (ppm)

2D NMR HSQC

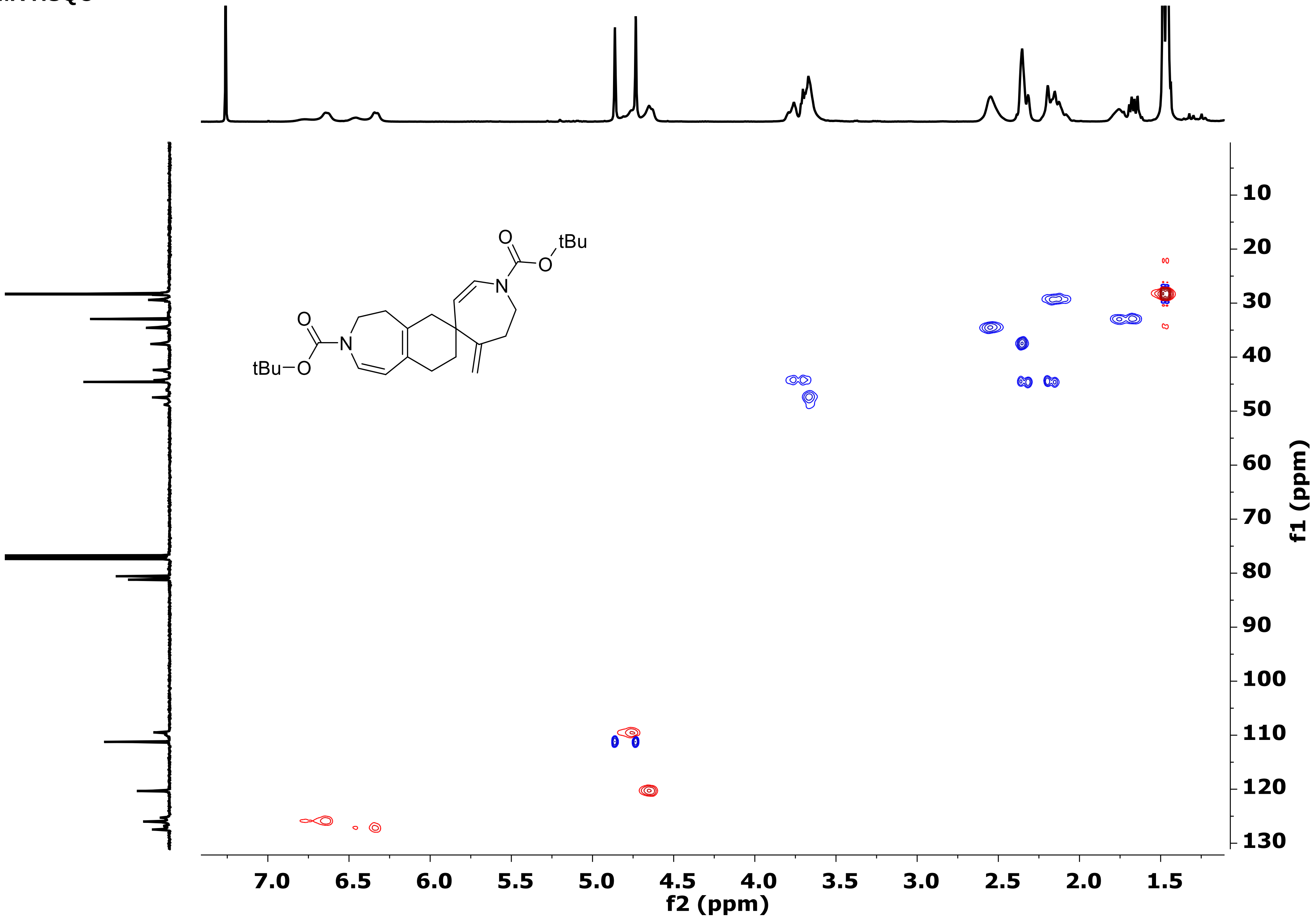
tBu
O
O
N
N
O
tBu–O
O
7.0
6.5
6.0
5.5
5.0
4.5
4.0
3.5
3.0
2.5
2.0
1.5
f2 (ppm)
10
20
30
40
50
60
70
80
90
100
110
120
130
f1 (ppm)

2D NMR COSY

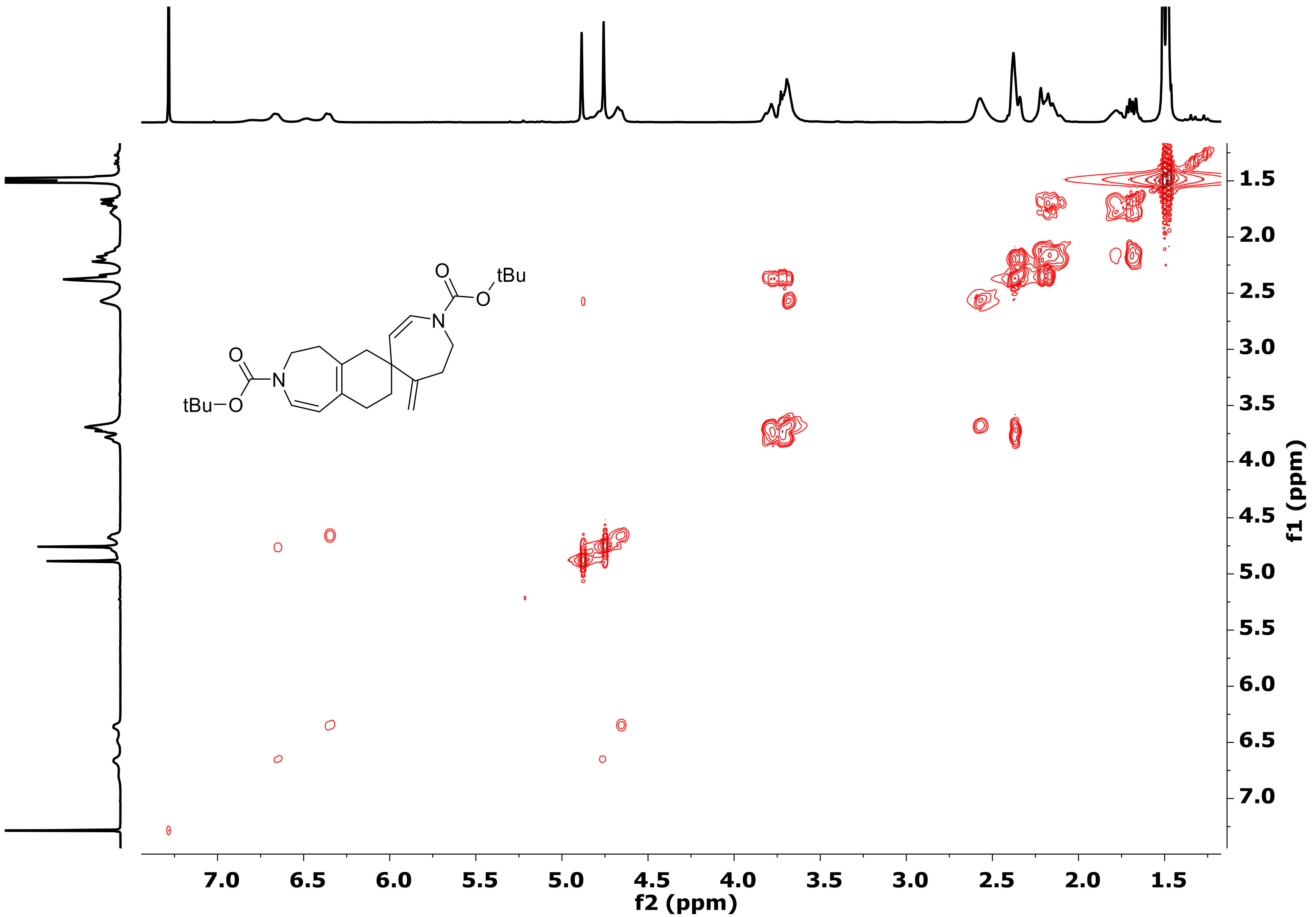

^{1}H NMR (400 MHz, CDCl$_3$) comparison of experiments at 298K (1) and 263K (2)

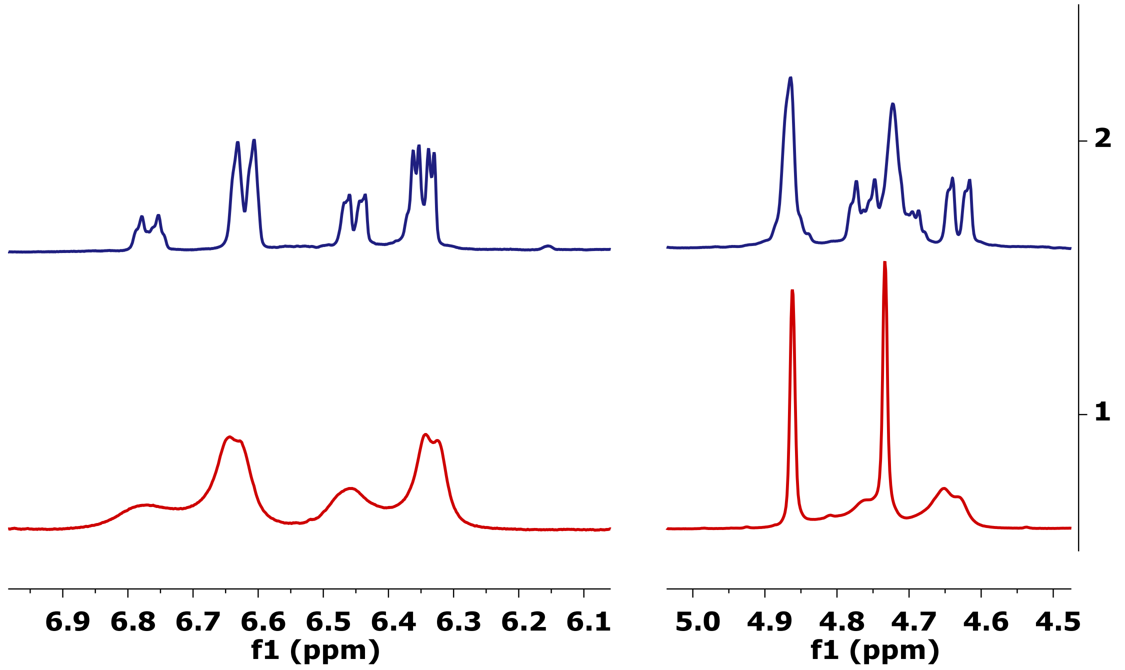

References

(1) Artigas, A.; Castanyer, C.; Roig, N.; Lledó, A.; Solà, M.; Pla-Quintana, A.; Roglans, A. Synthesis of fused dihydroazepine derivatives of fullerenes by a Rh-catalyzed cascade process. *Adv. Synth. Catal.* **2021**, *363*, 3835-3844.

(2) Horiuchi, T.; Nagata, M.; Kitagawa, M.; Akahane, K.; Uoto, K. Discovery of novel thieno[2,3-d]pyrimidin-4-yl hydrazone-based inhibitors of cyclin D1-CDK4: synthesis, biological evaluation and structure-activity relationships. Part 2. *Bioorg. Med. Chem.* **2009**, *17*, 7850-7860.

(3) Gaussian 16, Revision A.03, Frisch, M. J.; Trucks, G. W.; Schlegel, H. B.; Scuseria, G. E.; Robb, M. A.; Cheeseman, J. R.; Scalmani, G.; Barone, V.; Petersson, G. A.; Nakatsuji, H.; Li, X.; Caricato, M.; Marenich, A. V.; Bloino, J.; Janesko, B. G.; Gomperts, R.; Mennucci, B.; Hratchian, H. P.; Ortiz, J. V.; Izmaylov, A. F.; Sonnenberg, J. L.; Williams-Young, D.; Ding, F.; Lipparini, F.; Egidi, F.; Goings, J.; Peng, B.; Petrone, A.; Henderson, T.; Ranasinghe, D.; Zakrzewski, V. G.; Gao, J.; Rega, N.; Zheng, G.; Liang, W.; Hada, M.; Ehara, M.; Toyota, K.; Fukuda, R.; Hasegawa, J.; Ishida, M.; Nakajima, T.; Honda, Y.; Kitao, O.; Nakai, H.; Vreven, T.; Throssell, K.; Montgomery, J. A., Jr.; Peralta, J. E.; Ogliaro, F.; Bearpark, M. J.; Heyd, J. J.; Brothers, E. N.; Kudin, K. N.; Staroverov, V. N.; Keith, T. A.; Kobayashi, R.; Normand, J.; Raghavachari, K.; Rendell, A. P.; Burant, J. C.; Iyengar, S. S.; Tomasi, J.; Cossi, M.; Millam, J. M.; Klene, M.; Adamo, C.; Cammi, R.; Ochterski, J. W.; Martin, R. L.; Morokuma, K.; Farkas, O.; Foresman, J. B.; Fox, D. J. Gaussian, Inc., Wallingford CT, 2016.

(3) Stephens, P. J.; Devlin, F. J.; Chabalowski, C. F.; Frisch, M. J. Ab Initio calculation of vibrational absorption and circular dichroism spectra using Density Functional Force Fields. *J. Phys. Chem.* **1994**, *98*, 11623–11627.

(4) Becke, A. D. Density-functional thermochemistry. III. The Role of exact exchange. *J. Chem. Phys.* **1993**, *98*, 5648–5652.

(5) Lee, C.; Yang, W.; Parr, R. G. Development of the Colle-Salvetti correlation-energy formula into a functional of the electron density. *Phys. Rev. B* **1988**, *37*, 785–789.

(6) Dunning, T. H. Gaussian Basis sets for use in correlated molecular calculations. I. The atoms boron through neon and hydrogen. *J. Chem. Phys.* **1989**, *90*, 1007–1023.

(7) Marenich, A. V.; Cramer, C. J.; Truhlar, D. G. Universal solvation model based on solute electron density and on a continuum model of the solvent defined by the bulk dielectric constant and atomic surface tensions. *J. Phys. Chem. B* **2009**, *113*, 6378–6396.

(8) Grimme, S.; Antony, J.; Ehrlich, S.; Krieg, H. A Consistent and accurate Ab initio parametrization of density functional dispersion correction (DFT-D) for the 94 elements H-Pu. *J. Chem. Phys.* **2010**, *132*, 154104.

(9) Atkins, P.; De Paula, J. *The Elements of Physical Chemistry*, 3rd ed.; Oxford University Press: Oxford, 2006.
